# Decoupling between activation time and steady-state level in input-output responses

**DOI:** 10.1371/journal.pcbi.1014288

**Published:** 2026-05-15

**Authors:** Giorgio Ravanelli, Kee-Myoung Nam, Jeremy Gunawardena, Rosa Martinez-Corral

**Affiliations:** 1 CRG (Barcelona Collaboratorium for Modelling and Predictive Biology), Barcelona, Spain; 2 Department of Medicine and Life Sciences, Universitat Pompeu Fabra, Barcelona Biomedical Research Park, Barcelona, Spain; 3 Department of Systems Biology, Harvard Medical School, Boston, Massachusetts, United States of America; University of York, UNITED KINGDOM OF GREAT BRITAIN AND NORTHERN IRELAND

## Abstract

Many biological processes, like gene regulation or cell signalling, rely on molecules (inputs) that bind to targets leading to downstream responses. In the gene regulation field, recent data have shown that higher transcription factor (TF) concentrations may increase transcription levels of a gene without affecting the gene activation time. We call this behaviour *output decoupling*. Motivated by these observations, here we investigate mechanisms for output decoupling in Markov process models where a readout molecule is produced downstream of ligand binding. Our focus is on identifying regimes where the steady-state level of the readout changes with input concentration, while the activation time, quantified by mean first-passage times, remains unaffected. Through a combination of analytical and numerical investigations, we find two mechanisms through which output decoupling can arise: i) *rate scale separation*, where the system is comprised of slow and fast transitions that are differentially regulated by the input; and ii) *incoherent regulation*, where the input acts on two transitions with opposing effects on readout production, when all transition rates are similar. Such incoherent regulation has emerged as a plausible regulatory mode of TFs, and we suggest decoupling as a new characteristic feature of this regulatory mode. More broadly, our findings offer a mechanistic and conceptual framework for reasoning about output decoupling in input-output systems.

## Author summary

How biomolecular systems respond to signals often depends not only on the final level of activity they reach, but also on the initial activation dynamics. These two outputs—steady-state level and activation time—are usually coupled: higher input concentration tends to produce both higher activity and faster responses. Yet recent experiments suggest that, in some cases, the strength of the response can change while the speed remains constant. In this work, we explore the conditions under which such “output decoupling” can occur. Using mathematical models of molecular systems, we identify two ways this can happen. In one case, the activation time is determined by slow steps in the system that the input does not control. In the other, the input exerts opposing effects on the system, simultaneously promoting and hindering the response, which balances the timing. By revealing how decoupling arises, our study provides a framework for interpreting puzzling experimental results. More broadly, it shows the value of jointly considering both dynamics and steady-state behaviour when studying how molecular systems process information.

## Introduction

**Fig 1:**
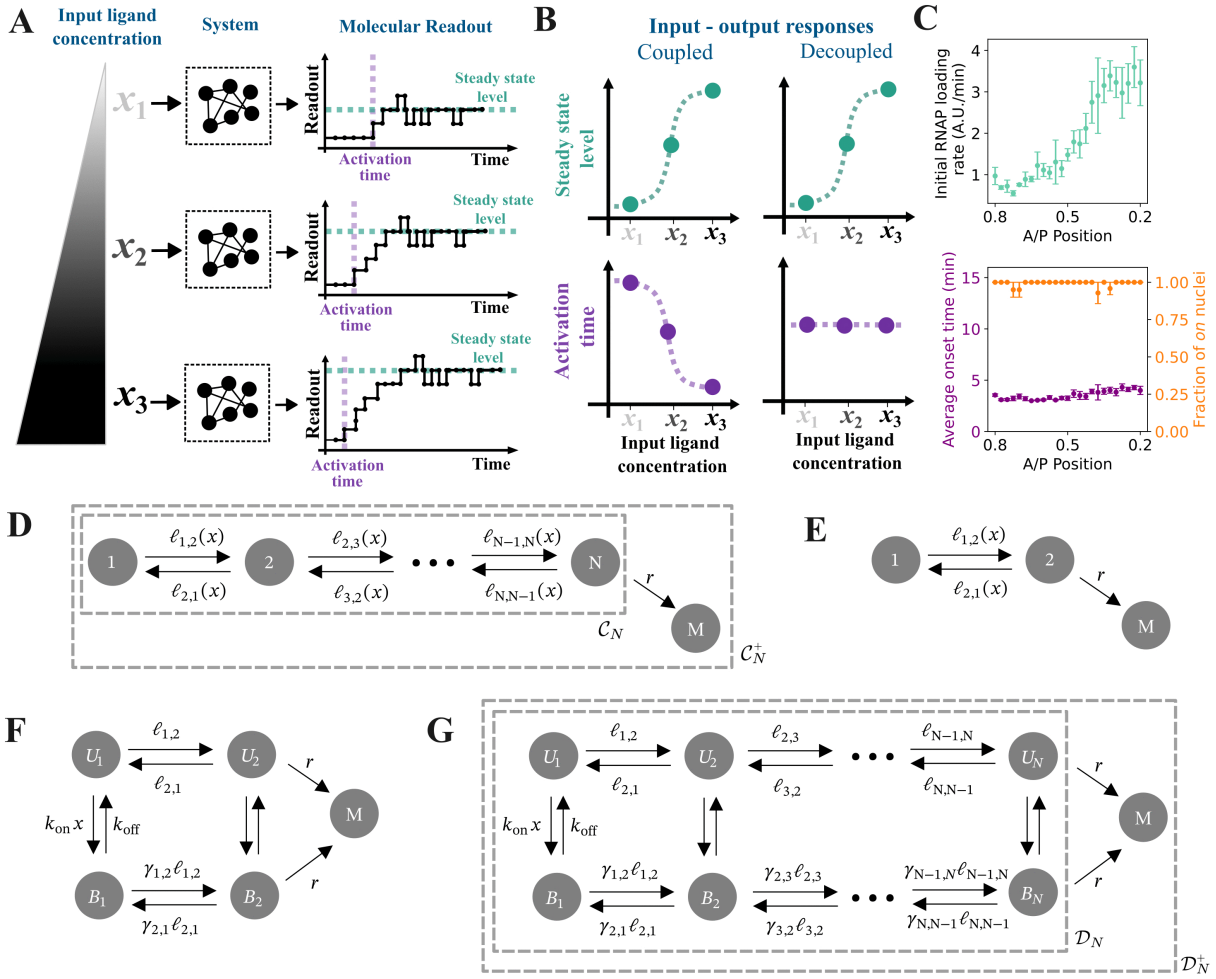
Interplay between steady state and activation time. **(A)** Schematic of input-output responses. An input ligand with concentration *x* (left) is processed by a system from which we measure a molecular readout, from which the readout’s steady-state level or activation time can be quantified (right). Here, “activation time” is defined as the time required for the readout to increase by one molecule after the input has been introduced. **(B)** Schematic of coupled and decoupled input-output responses for the steady-state level and activation time, given that the input is an activator. In the former, the steady-state level increases while the activation time decreases with input concentration; in the latter, the steady-state level increases with the input concentration, while the activation time remains constant. **(C)** Measurements of RNA polymerase loading rate (top), average transcription onset time (bottom, purple), and fraction of reporter-expressing nuclei (bottom, orange) for a reporter MS2 construct with the wild-type *hunchback* promoter, along the antero-posterior axis of the *Drosophila melanogaster* blastoderm (nuclear cycle 13), reproduced from [9, Fig 4]. Low “A/P position” corresponds to the anterior end of the embryo, where the Bicoid concentration is high; high values correspond to the posterior end of the embryo, where the Bicoid concentration is low. **(D–G)** The models used in this paper, where *M* is the molecular readout and *x* denotes the input concentration. See text for more details. **(D–E)** Chain models with implicit ligand binding. The ligand’s regulatory effect is captured in the edge labels as arbitrary functions of *x*. The graph, ${𝒞}_{N}$, without the terminal state *M* is used to calculate the steady-state level; the augmented graph, ${𝒞}_{N}^{+}$, is used to calculate the activation time. See text for more details. **(F–G)** Ladder models that explicitly incorporate ligand binding. The vertical edges represent ligand binding and unbinding, with rates *k*_on_*x* and *k*_off_, respectively. As with the chain models, the graph, ${𝒟}_{N}$, without the terminal state *M* is used to calculate the steady-state level, while the augmented graph, ${𝒟}_{N}^{+}$, is used to calculate the activation time. See text for more details.

Many biological processes are regulated by an input molecule that binds to a target. Examples include ligands binding to receptors, transcription factors (TFs) binding to regulatory sites on DNA, and splicing regulators binding to pre-mRNAs. Upon binding, the molecular system can undergo internal molecular changes that result in a measurable readout (Fig 1A). Example readouts can be a receptor’s phosphorylation level, a gene’s expression level, or an exon’s inclusion level. Usually, we extract summary features, or “outputs”, from the molecular readout and study their relationships with the input levels (Fig 1A,B). We call these types of mappings *input-output responses* [1–5].

Two outputs commonly considered in the literature are the *steady-state* *level* of the molecular readout, and the *activation time*. The latter can be defined and quantified in different ways (Discussion). In this work, we consider the activation time as the time for the molecular readout level to change upon introducing the input (Fig 1A), and we will define it formally below. When both the steady-state and activation time have been quantified as functions of input concentration, experiments have typically shown *coupling*: a higher steady-state level is usually accompanied by faster (smaller) activation times (Fig 1B, *Coupled*). Examples include β-adrenergic receptor activity as a function of drug concentration [6], viral entry as a function of receptor concentration [7], and transcription of various genes as a function of TF concentration [8–13].

Coupling can be intuitively explained by thinking about a system that undergoes a series of reversible transitions between an inactive and an active state from which the molecular readout is produced, with the binding of the input promoting (accelerating) the transitions towards the productive state [9,14,15]. In this scenario, higher input concentrations reduce the time for the readout to change while also increasing its steady-state level.

By contrast, experimental data from recent studies of gene regulation in the *Drosophila* blastoderm suggest the possibility of *output decoupling*, where the transcription level of a gene increases as a function of the concentration of an input TF, but the activation time does not change (Fig 1B, *Decoupled*). For example, such decoupling has been observed in the regulation of *hunchback* [9]. This gene is activated by the TF Bicoid, which exhibits a concentration gradient over the antero-posterior axis of the embryo. Eck et al. [9] observed that the transcriptional output (initial polymerase loading rate) of a *hunchback* reporter is position-dependent, whereas the activation time is not (Fig 1C). Similar observations have been made in other studies of this [16] and other genes [11,14]. This decoupling may serve valuable roles in development, for instance by enabling temporal coordination of gene expression programmes across tissue regions exposed to different morphogen concentrations. Decoupling may also be a desirable feature in synthetic biology applications, where different output levels are to be achieved at a given time, or simultaneously over a given population of cells. Although specific models have been proposed in some of the aforementioned studies (Discussion), a theoretical understanding of the fundamental mechanisms and key features required for generating decoupling is missing.

To explore this question, we focus on processes in which a ligand binds to a target and promotes the downstream production or accumulation of a molecule. This accounts for TFs activating transcription, or a ligand-bound receptor influencing protein modification or altering the permeability of a channel or transporter. For generality, we use the term “ligand” to refer to the input molecule, although we mostly frame the work in the language of gene regulation, given the motivating observations in that field.

We begin by giving a general overview of the models, namely Markov processes, that we examine and the mathematical formalism, namely the graph-theoretic *linear framework* [17–21], that we use to analyse them. We start with the simple two-state telegraph model, then proceed to more complex models. By employing a dialogue between numerical approaches and analytical calculations, we find two regulatory strategies for output decoupling: i) when the system exhibits *rate scale separation*, in which a slower forward transition or set of forward transitions govern the activation time, with the input affecting the steady state by modulating other transitions, and ii) when the system exhibits an *incoherent regulatory mode*, which simultaneously promotes and hinders production of the readout. This regulatory mode has recently garnered attention in the field of eukaryotic gene regulation [22–24], and our results suggest that output decoupling could be another significant consequence of such incoherent regulation. More generally, we demonstrate that a rich mechanistic playground is uncovered when jointly investigating the steady state and the transient regime of molecular systems, thus further motivating joint experimental and theoretical investigation of these two regimes.

## Modelling approach and mathematical setup

### Linear framework models

We model ligand-binding-readout systems as discrete-state, continuous-time Markov processes using the graph-theoretic linear framework [17–21]. This framework describes the dynamical properties of a Markov process on a graph, such as its steady-state behaviour or its first-passage times, in terms of the structural properties of the graph. Crucially, this framework allows for the derivation of exact, closed-form expressions for these dynamical properties, obviating the need for specifying numerical values for model parameters. We have extensively exploited these capabilities in previous studies to analyse various types of input-output systems, including those with multiple binding sites [5] and conformations [24,25].

We start by describing the system as a finite, directed graph, *G*, with labelled edges, in which the vertices, 𝒱(G), represent the system states; the edges, denoted i→j, represent transitions among the states; and the edge labels, denoted ℓ(i→j), represent transition rates with dimensions of ${\left(\text{time}\right)}^{-1}$. Such a description gives rise to a corresponding continuous-time Markov process, X(·), on state space 𝒱(G), in which the edges represent possible transitions between these states, with each edge label as the corresponding transition rate. To be more precise, the infinitesimal transition rate from *i* to *j* is nonzero if, and only if, the edge i→j exists in *G*, and the rate is given by the edge label:


$$\begin{array}{c} \lim_{h\to 0}\frac{\mathrm{Pr}\left(X(t+h)=j|X(t)=i\right)}{h}=\ell (i\to j). \end{array}$$

We note that the edge labels, ℓ(i→j), are assumed to be constant in time. As such, the transition rates in X(·) are constant, i.e., X(·) is a time-homogeneous Markov process.

Now, suppose that 𝒱(G)={1,…,n}, and let *p*_*i*_(*t*) be the probability that X(·) occupies vertex i∈𝒱(G) at time *t*, given some choice of initial vertex. The time-evolution of this probability is given by the master equation [26,18],


$$\begin{array}{c} \frac{d}{dt}𝐩(t)=ℒ(G)\;𝐩(t), \end{array}$$ (1)

where $𝐩(t)={\left(p_{1}(t),\ldots ,p_{n}(t)\right)}^{T}$ and ℒ(G) is the *n* × *n* *Laplacian matrix* of *G*, whose entries are given by


$$\begin{array}{c} ℒ(G{)}_{i,j}=\left\{ \begin{array}{cc} 0 & \text{if}\;i\neq j\;\text{and}\;j\text{⧸}\to i\\ \ell (j\to i) & \text{if}\;i\neq j\;\text{and}\;j\to i\\ -\sum_{k\in 𝒱(G):i\to k}\ell (i\to k) & \text{if}\;i=j. \end{array}\right. \end{array}$$ (2)

**Fig 2:**
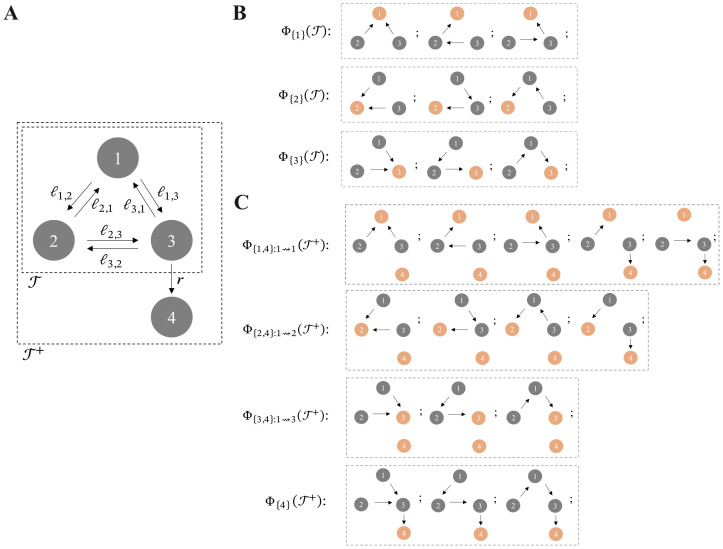
Using spanning trees and forests to calculate steady-state responses and activation times. **(A)** An example three-vertex graph, 𝒯, and a corresponding augmented graph, ${𝒯}^{+}$, as discussed in the text. Here, ${𝒱}_{\text{prod}}(𝒯)=\left\{3\right\}$. **(B)** The spanning trees of 𝒯 rooted at 1, 2 and 3. Roots are shown in orange. These spanning trees contribute to the calculation of the steady-state response, through Eq. 9; see the text for details. **(C)** The spanning forests of ${𝒯}^{+}$ rooted at {*j*,4}, for each *j* = 1, 2, 3, in which there is a path from vertex 1 to vertex *j* (top three rows); and the spanning trees of ${𝒯}^{+}$ rooted at 4 (bottom row). Roots are shown in orange. These spanning forests contribute to the calculation of the activation time, through Eq. 10; see the text for details.

For instance, we can use this formula to see that the Laplacian matrix of the three-vertex graph, 𝒯, in Fig 2A is the following 3 × 3 matrix,


$$\begin{array}{c} ℒ(𝒯)=\left[\begin{array}{ccc} -\ell_{1,2}-\ell_{1,3} & \ell_{2,1} & \ell_{3,1}\\ \ell_{1,2} & -\ell_{2,1}-\ell_{2,3} & \ell_{3,2}\\ \ell_{1,3} & \ell_{2,3} & -\ell_{3,1}-\ell_{3,2} \end{array}\right]. \end{array}$$ (3)

We will consider two kinds of models in our analysis. First, we consider *chain models* (also called *pipeline models* in previous work [20,21]), denoted ${𝒞}_{N}$, in which *N* vertices are reversibly connected in a linear arrangement. In particular, the vertices of ${𝒞}_{N}$ are given by $𝒱({𝒞}_{N})=\left\{1,\ldots ,N\right\}$, and consecutive vertices are connected by reversible edges, i→i+1 and i+1→i for i=1,…,N−1 (Fig 1D–E). Each vertex is assumed to correspond to a different state of the system, including the presence or absence of bound ligand, in addition to other internal features. For example, in a receptor system, a state could correspond to a given conformation or post-translational modification state. In a gene regulation system, states could correspond to DNA conformations, nucleosome patterns, polymerase occupancy at the promoter, and other salient features. The binding of the ligand is modelled through the edge labels, which can be generic functions of the ligand concentration, $\ell (i\to j)=\ell_{i,j}(x)$ (Fig 1D–E). For the sake of mechanistic generality, we leave the functional form of $\ell_{i,j}(x)$ unspecified.

In order to formalise a particular assumption for how the ligand influences the system, we turn to *ladder models*, denoted ${𝒟}_{N}$, which explicitly incorporate ligand binding to a single site, and assume that the ligand has an effect while bound (Fig 1F–G) [13,27]. In particular, ${𝒟}_{N}$ is a graph on 2*N* vertices,


$$\begin{array}{c} 𝒱({𝒟}_{N})=\left\{U_{1},\ldots ,U_{N},B_{1},\ldots ,B_{N}\right\}, \end{array}$$

where $U_{1},\ldots ,U_{N}$ represent ligand-unbound states and $B_{1},\ldots ,B_{N}$ represent ligand-bound states. There are edges between pairs of consecutive unbound vertices, $U_{i}\to U_{i+1}$ and $U_{i+1}\to U_{i}$; pairs of consecutive bound vertices, $B_{i}\to B_{i+1}$ and $B_{i+1}\to B_{i}$; and pairs of unbound and bound vertices of the same index, $U_{i}\to B_{i}$ and $B_{i}\to U_{i}$. We assume that the label on each binding edge, $U_{i}\to B_{i}$, and unbinding edge, $B_{i}\to U_{i}$, is independent of the index *i*, as


$$\begin{array}{c} \ell (U_{i}\to B_{i})=k_{\text{on}}x\;\text{and}\;\ell (B_{i}\to U_{i})=k_{\text{off}}, \end{array}$$

where *x* is the ligand concentration, *k*_on_ is a rate constant with units of ${\left(\text{concentration}\cdot \text{time}\right)}^{-1}$, and *k*_off_ is an off-rate. On the other hand, we allow for the other edge labels to vary with *i*, and write them as


$$\begin{array}{c} \ell (U_{i}\to U_{j})=\ell_{i,j}\;\text{and}\;\ell (B_{i}\to B_{j})=\gamma_{i,j}\ell_{i,j}. \end{array}$$

Here, $\gamma_{i,j}>0$ is a dimensionless parameter that captures the extent to which the ligand promotes ($\gamma_{i,j}>1$), hinders ($\gamma_{i,j}<1$), or maintains as is ($\gamma_{i,j}=1$) the transition from index *i* to index *j*. We call these parameters *regulatory factors*.

### Quantifying steady-state level and activation time

We now describe how we quantify the steady-state response and activation time of these systems. Each system we consider in this paper produces a molecular readout, *M*, whose copy-number we denote by *n*_*M*_. We assume that each graph, *G*, contains a subset of vertices, ${𝒱}_{\text{prod}}(G)\subset 𝒱(G)$, from which the system can produce *M* at a constant rate *r*. In addition, we assume that *M* undergoes first-order degradation, with rate $\delta n_{M}$. In this context, we can naturally define the steady-state level, SS(*x*), of *M* as the steady-state mean value of *n*_*M*_, which can be shown to be equal to (Appendix A in S1 Text) [28,29],


$$\begin{array}{c} SS(x)=\langle n_{M}\rangle^{*}=\frac{r}{\delta }\sum_{v\in {𝒱}_{\text{prod}}(G)}p_{v}^{*}, \end{array}$$ (4)

where $p_{v}^{*}$ is the steady-state probability of the vertex *v*. That is, $p_{v}^{*}$ is the *v*-th entry in the vector, ${𝐩}^{*}={\left(p_{1}^{*},\ldots ,p_{n}^{*}\right)}^{T}$, of probabilities that satisfies Eq. 1 with the left-hand time-derivative set to zero,


$$\begin{array}{c} ℒ(G)\;{𝐩}^{*}=0. \end{array}$$ (5)

Eq. 4 describes how the steady-state behaviour of the input-output system, as described by the graph *G*, determines the steady-state level of the readout, *M*, as a function of the input concentration, *x*. For the chain models, we assume that ${𝒱}_{\text{prod}}({𝒞}_{N})=\left\{N\right\}$; for the ladder models, we assume that ${𝒱}_{\text{prod}}({𝒟}_{N})=\left\{U_{N},B_{N}\right\}$.

To obtain $p_{v}^{*}$ for each $v\in {𝒱}_{\text{prod}}(G)$, Eq. 5 tells us that p^*^ lies in the kernel of ℒ(G). If *G* is *strongly connected*—that is, if every pair of vertices in *G* is connected by a path of directed edges—then one can show that this kernel has dimension 1 [18],


$$\begin{array}{c} \dim\ker ℒ(G)=1. \end{array}$$

In this case, p^*^ is unique, and can be obtained by identifying any vector, ρ, in kerℒ(G) by normalizing by the coordinate sum. The kinds of graphs we consider, ${𝒞}_{N}$ and ${𝒟}_{N}$, are strongly connected for all *N*. To calculate ρ in the numerical analyses we perform below, we obtained the singular value decomposition (SVD) of ℒ(G) and set ρ to the right singular vector corresponding to the zero singular value [30] (Materials and Methods).

On the other hand, we define the activation time of a system to produce one new molecule of *M* as a mean first-passage time (mFPT) on an augmented graph, *G*^+^, in which an additional “terminal” vertex is introduced to describe the production event. We also denote this new vertex by *M*, and identify it with *n* + 1 in the vertex ordering. We then define *G*^+^ as the graph on vertices $𝒱(G^{+})=𝒱(G)\cup \left\{M\right\}$ that is obtained by adding the edges $\begin{array}{c} \;j\to M \end{array}$, for $\;j\in {𝒱}_{\text{prod}}(G)$, each with the production rate, *r*, as the edge label. Fig 2A demonstrates this construction on our example graph, 𝒯, with ${𝒱}_{\text{prod}}(𝒯)=\left\{3\right\}$. The resulting augmented graph, ${𝒯}^{+}$, contains a new terminal vertex, *M* = 4, as well as the edge 3→4 with label ℓ(3→4)=r. From Eq. 2, we can see that the Laplacian matrix of ${𝒯}^{+}$ is the 4 × 4 matrix,


$$\begin{array}{c} ℒ({𝒯}^{+})=\left[\begin{array}{cccc} -\ell_{1,2}-\ell_{1,3} & \ell_{2,1} & \ell_{3,1} & 0\\ \ell_{1,2} & -\ell_{2,1}-\ell_{2,3} & \ell_{3,2} & 0\\ \ell_{1,3} & \ell_{2,3} & -\ell_{3,1}-\ell_{3,2}-r & 0\\ 0 & 0 & r & 0 \end{array}\right]. \end{array}$$ (6)

Now, let i∈𝒱(G) be a non-terminal vertex in *G*^+^, and let $X^{+}(\cdot )$ be the Markov process associated with *G*^+^. We define the activation time from vertex *i* as the mean time taken by $X^{+}(\cdot )$ to first reach *M* from *i*. In other words, the activation time from vertex *i* is the mFPT,


$$\begin{array}{c} {mFPT}^{i}(x)=𝔼\;\left[\;\inf\left\{t>0:X^{+}(t)=M\right\}|X^{+}(0)=i\;\right]. \end{array}$$ (7)

This mFPT can be obtained from the matrix equation (Appendix A in S1 Text) [21],


$$\begin{array}{c} {\left(𝐋(G^{+}{)}_{\left[\overset{―}{\left\{n+1\right\}},\overset{―}{\left\{n+1\right\}}\right]}\right)}^{2}\left[\begin{array}{c} {mFPT}^{1}(x)\\ \vdots \\ {mFPT}^{n}(x) \end{array}\right]=\left[\begin{array}{c} \ell (1\to n+1)\\ \vdots \\ \ell (n\to n+1) \end{array}\right], \end{array}$$ (8)

where we have introduced $𝐋(G^{+})=-ℒ(G^{+}{)}^{T}$, and $𝐋(G^{+}{)}_{\left[\overset{―}{\left\{n+1\right\}},\overset{―}{\left\{n+1\right\}}\right]}$ is the *n* × *n* sub-matrix of **L**(*G*^+^) obtained by removing the row and column corresponding to M≡n+1. Briefly, this matrix equation is obtained by defining the adjoint master equation of $X^{+}(\cdot )$ and taking its Laplace transform (Appendix A in S1 Text). For our numerical analyses below, we solved for the vector of mFPTs in Eq. 8 by obtaining the QR decomposition of the left-hand matrix [30] (Materials and Methods).

Let us consider how this calculation bears out for our example graph, ${𝒯}^{+}$. Taking the negative transpose of the Laplacian matrix, $ℒ({𝒯}^{+})$, in Eq. 6 and removing the row and column corresponding to *M* = 4, we get


$$\begin{array}{c} 𝐋({𝒯}^{+}{)}_{\left[\overset{―}{\left\{4\right\}},\overset{―}{\left\{4\right\}}\right]}=\left[\begin{array}{ccc} \ell_{1,2}+\ell_{1,3} & -\ell_{1,2} & -\ell_{1,3}\\ -\ell_{2,1} & \ell_{2,1}+\ell_{2,3} & -\ell_{2,3}\\ -\ell_{3,1} & -\ell_{3,2} & \ell_{3,1}+\ell_{3,2}+r \end{array}\right]. \end{array}$$

As such, the vector of mFPTs to *M* from the other three vertices satisfies the equation (Eq. 8),


$$\begin{array}{c} {\left[\begin{array}{ccc} \ell_{1,2}+\ell_{1,3} & -\ell_{1,2} & -\ell_{1,3}\\ -\ell_{2,1} & \ell_{2,1}+\ell_{2,3} & -\ell_{2,3}\\ -\ell_{3,1} & -\ell_{3,2} & \ell_{3,1}+\ell_{3,2}+r \end{array}\right]}^{2}\left[\begin{array}{c} {\text{mFPT}}^{1}\\ {\text{mFPT}}^{2}\\ {\text{mFPT}}^{3} \end{array}\right]=\left[\begin{array}{c} 0\\ 0\\ r \end{array}\right]. \end{array}$$

### Analytical formulas for SS(*x*) and mFPT^*i*^(*x*)

As described above, it is straightforward to solve Eqs. 5 and 8 numerically, given particular values of the edge labels, using standard techniques. Complementing this approach, we also sought to obtain analytical formulas for SS(*x*) and mFPT^*i*^(*x*), both of which are accessible via the Matrix-Tree theorems [18,21]. To understand these formulas, we must first consider the *spanning forests* of a graph. For any graph Γ, a *spanning forest* of Γ is a subgraph, *F*, that (1) contains all the vertices in Γ (“spanning”); (2) contains no cycles, even when ignoring edge directions (“forest”); and (3) contains exactly one outgoing edge from each vertex other than a subset, ℛ(F)⊂𝒱(Γ), from which there are no outgoing edges. This subset of vertices are called the *roots* of *F*. If ℛ(F) consists of a single vertex, then *F* is a *spanning tree*. We denote the set of spanning forests of Γ rooted at A⊂𝒱(Γ) by $\Phi_{A}(\Gamma )$. Returning to our running example, we show in Fig 2B the set of spanning trees of 𝒯 that are rooted at each of the vertices 1, 2 and 3, which we denote by $\Phi_{\left\{1\right\}}(𝒯)$, $\Phi_{\left\{2\right\}}(𝒯)$ and $\Phi_{\left\{3\right\}}(𝒯)$, respectively.

Since there is exactly one outgoing edge from each non-root vertex in any spanning forest *F*, it is easy to see that *F* must contain a directed path of edges, $i\to i_{1}\to \cdots \to i_{k}\to j$, from each vertex, i∉ℛ(F), to precisely one root, $\begin{array}{c} \;j\in ℛ(F) \end{array}$. For instance, if *F* is a spanning tree rooted at ℛ(F)={j}, then every vertex must have a path to *j* in *F*. On the other hand, each root in any spanning forest has a trivial path to itself and, since roots lack outgoing edges, evidently has no path to any other root. Now, given A⊂𝒱(Γ) and j∈A, we denote by $\Phi_{A:i⇝j}(\Gamma )\subset \Phi_{A}(\Gamma )$ the subset of spanning forests rooted at *A* in which there is a path from *i* to *j*. Here, *i* may either be any non-root vertex or *j* itself, so that i∈(𝒱(Γ)⧵A)∪{j}. If *i* = *j*, then, as described above, *j* always has a trivial path to itself and has no path to any other root, and so we have $\Phi_{A:j⇝j}(\Gamma )=\Phi_{A}(\Gamma )$.

Fig 2C shows four subsets of spanning forests of our example augmented graph, ${𝒯}^{+}$, each with a different set of roots ({1,4}, {2,4}, {3,4}, and {4}) and a different choice of root to which there is a path from the vertex 1. For instance, the second row contains the spanning forests of ${𝒯}^{+}$ that are rooted at {2,4} and contain a path from 1 to 2; following our notation, this is the set $\Phi_{\{2,4\}:1⇝2}({𝒯}^{+})$. Note that, in the first row, we show the set $\Phi_{\{1,4\}:1⇝1}({𝒯}^{+})$ of spanning forests rooted at {1,4} with a path from 1 to itself; as described above, since such a trivial path exists in every spanning forest rooted at {1,4}, this set is equal to the set $\Phi_{\left\{1,4\right\}}({𝒯}^{+})$. Similarly, the last row shows the set $\Phi_{\left\{4\right\}}({𝒯}^{+})$ of spanning trees rooted at 4; since every vertex has a path to the lone root in a spanning tree, this set is also equal to $\Phi_{\{4\}:1⇝4}({𝒯}^{+})$.

We are now ready to provide our formulas for SS(*x*) and mFPT^*i*^(*x*). For the former, the Matrix-Tree theorem tells us that the steady-state probability vector, p^*^, in Eq. 5 is given by [17–19]


$$\begin{array}{c} {𝐩}^{*}=\frac{\rho }{\rho_{1}+\cdots +\rho_{n}},\;\text{with}\;\rho_{i}=w(\Phi_{\left\{i\right\}}(G)), \end{array}$$ (9)

where w(·) is the *weight* function, which, here, evaluates the sum of products of edge labels over each spanning forest in $\Phi_{\left\{i\right\}}(G)$. More broadly, if ℋ is any collection of graphs, then w(ℋ) is defined as


$$\begin{array}{c} w(ℋ)=\sum_{H\in ℋ}\left(\prod_{u\to v\in H}\ell (u\to v)\right). \end{array}$$

To see how this calculation works on 𝒯, we can run through the spanning trees in Fig 2B and read off the corresponding edge labels in Fig 2A, to get


$$\begin{array}{c} \begin{array}{ccc} \rho_{1}= & \;\;\;\;w(\Phi_{\left\{1\right\}}(𝒯))= & \;\;\;\;\ell_{2,1}\ell_{3,1}+\ell_{2,1}\ell_{3,2}+\ell_{2,3}\ell_{3,1}\\ \rho_{2}= & \;\;\;\;w(\Phi_{\left\{2\right\}}(𝒯))= & \;\;\;\;\ell_{1,2}\ell_{3,2}+\ell_{1,3}\ell_{3,2}+\ell_{1,2}\ell_{3,1}\\ \rho_{3}= & \;\;\;\;w(\Phi_{\left\{3\right\}}(𝒯))= & \;\;\;\;\ell_{1,3}\ell_{2,3}+\ell_{1,2}\ell_{2,3}+\ell_{2,1}\ell_{1,3}. \end{array} \end{array}$$

We can then use Eq. 9 to calculate p^*^, then use Eq. 4 to solve for SS(*x*). In particular, since ${𝒱}_{\text{prod}}(𝒯)=\left\{3\right\}$, Eq. 4 tells us that


$$\begin{array}{c} SS(x)=\frac{rp_{3}^{*}}{\delta }=\frac{r}{\delta }\left(\frac{\rho_{3}}{\rho_{1}+\rho_{2}+\rho_{3}}\right). \end{array}$$

As for the mFPTs, we can use the All-Minors Matrix-Tree theorem to show that [21]


$$\begin{array}{c} {mFPT}^{i}(x)=\sum_{j\in 𝒱(G)}\frac{w(\Phi_{\{j,M\}:i⇝j}(G^{+}))}{w(\Phi_{\left\{M\right\}}(G^{+}))}, \end{array}$$ (10)

where, as described above, *G*^+^ is obtained by augmenting *G* with the terminal vertex, *M* (Appendix A in S1 Text). For instance, applying this formula to our example augmented graph, ${𝒯}^{+}$, with *i* = 1 yields


$$\begin{array}{c} {mFPT}^{1}=\sum_{j\in \{1,2,3\}}\frac{w(\Phi_{\{j,4\}:1⇝j}({𝒯}^{+}))}{w(\Phi_{\left\{4\right\}}({𝒯}^{+}))}. \end{array}$$ (11)

The spanning forests of ${𝒯}^{+}$ that contribute to the numerator are given in the first three rows of Fig 2C; the spanning trees that contribute to the denominator are given in the last row. Reading off the edge labels in Fig 2A, we can see that these terms are given by


$$\begin{array}{c} \begin{array}{ccc} & w(\Phi_{\{1,4\}:1⇝1}({𝒯}^{+}))\;\;\;\; & =\ell_{2,1}\ell_{3,1}+\ell_{2,1}\ell_{3,2}+\ell_{2,3}\ell_{3,1}+\ell_{2,1}r+\ell_{2,3}r\\ & w(\Phi_{\{2,4\}:1⇝2}({𝒯}^{+}))\;\;\;\; & =\ell_{1,2}\ell_{3,2}+\ell_{1,3}\ell_{3,2}+\ell_{1,2}\ell_{3,1}+\ell_{1,2}r\\ & w(\Phi_{\{3,4\}:1⇝3}({𝒯}^{+}))\;\;\;\; & =\ell_{1,3}\ell_{2,3}+\ell_{1,2}\ell_{2,3}+\ell_{1,3}\ell_{2,1}\\ & \;w(\Phi_{\left\{4\right\}}({𝒯}^{+}))\;\;\;\; & =\ell_{1,3}\ell_{2,3}r+\ell_{1,2}\ell_{2,3}r+\ell_{1,3}\ell_{2,1}r, \end{array} \end{array}$$

which we can substitute into Eq. 11 to obtain an expression for mFPT^1^.

Eqs. 9 and 10 demonstrate that analytical formulas for both quantities, in terms of the edge labels of the underlying graph, can be obtained through spanning tree/forest enumeration, as we have done with 𝒯 and ${𝒯}^{+}$. However, such enumeration can be prohibitively expensive even for simple graphs. To circumvent this, we turn to a recurrence relation due to Chebotarev and Agaev [31], which we summarise here and describe in more detail in Appendix A in S1 Text.

For any graph Γ with vertices V(Γ)={1,…,m}, Chebotarev and Agaev defined a sequence of *m* × *m* matrices, ${𝐐}_{k}(\Gamma )$, in which the (*i*,*j*)-th entry, denoted by $q_{i,j}^{\left(k\right)}(\Gamma )$, is the total weight of the spanning forests of Γ with the following properties: (1) it contains *k* edges, (2) *j* is one of its roots, and (3) it contains a path from *i* to *j*. Upon evaluating these matrices for *G* and *G*^+^, we can rewrite Eqs. 9 and 10 in terms of these matrices (Appendix A in S1 Text). First, we can rewrite our expression for $\rho_{i}$ in Eq. 9 as


$$\begin{array}{c} \rho_{i}=q_{i,i}^{\left(n-1\right)}(G). \end{array}$$ (12)

Second, we can rewrite Eq. 10 as


$$\begin{array}{c} {mFPT}^{i}(x)=\sum_{j\in 𝒱(G)}\frac{q_{i,j}^{\left(n-1\right)}(G^{+})}{q_{n+1,n+1}^{\left(n\right)}(G^{+})}. \end{array}$$ (13)

Now, these reformulations are useful because Chebotarev and Agaev showed that these matrices follow the recurrence relation,


$$\begin{array}{c} {𝐐}_{k+1}(\Gamma )=-𝐋(\Gamma )\;{𝐐}_{k}(\Gamma )+\left(\frac{tr\left(𝐋(\Gamma )\;{𝐐}_{k}(\Gamma )\right)}{k+1}\right)𝐈, \end{array}$$ (14)

with the initial condition ${𝐐}_{0}(\Gamma )=𝐈$. This means that we can calculate ${𝐐}_{n-1}(G)$, ${𝐐}_{n-1}(G^{+})$, and ${𝐐}_{n}(G^{+})$ by iteratively applying Eq. 14, then use their entries to calculate $\rho_{i}$ in Eq. 12 and therefore the steady-state response (Eqs. 4 and 9), as well as the mFPT with Eq. 13. Crucially, these equations constitute an algorithm with a polynomial runtime in the number of vertices, *n*, that circumvents the need to enumerate spanning trees and forests.

Let us now consider how this calculation proceeds on 𝒯 and ${𝒯}^{+}$. First, taking the negative transpose of ℒ(𝒯) in Eq. 3 and $ℒ({𝒯}^{+})$ in Eq. 6, we get


$$\begin{array}{c} 𝐋(𝒯)=\left[\begin{array}{ccc} \ell_{1,2}+\ell_{1,3} & -\ell_{1,2} & -\ell_{1,3}\\ -\ell_{2,1} & \ell_{2,1}+\ell_{2,3} & -\ell_{2,3}\\ -\ell_{3,1} & -\ell_{3,2} & \ell_{3,1}+\ell_{3,2} \end{array}\right] \end{array}$$

and


$$\begin{array}{c} 𝐋({𝒯}^{+})=\left[\begin{array}{cccc} \ell_{1,2}+\ell_{1,3} & -\ell_{1,2} & -\ell_{1,3} & 0\\ -\ell_{2,1} & \ell_{2,1}+\ell_{2,3} & -\ell_{2,3} & 0\\ -\ell_{3,1} & -\ell_{3,2} & \ell_{3,1}+\ell_{3,2}+r & -r\\ 0 & 0 & 0 & 0 \end{array}\right]. \end{array}$$

Noting that n=#𝒱(𝒯)=3, we can now apply Eq. 14 to compute ${𝐐}_{2}(𝒯)$, ${𝐐}_{2}({𝒯}^{+})$ and ${𝐐}_{3}({𝒯}^{+})$. The resulting matrices are rather complicated, but the reader can easily undertake these calculations to verify that the entries required in Eqs. 12 and 13 are given by


$$\begin{array}{c} \begin{array}{cc} \;q_{1,1}^{\left(2\right)}(𝒯)\;\;\;\; & =\ell_{2,1}\ell_{3,1}+\ell_{2,1}\ell_{3,2}+\ell_{2,3}\ell_{3,1}\\ \;q_{2,2}^{\left(2\right)}(𝒯)\;\;\;\; & =\ell_{1,2}\ell_{3,2}+\ell_{1,2}\ell_{3,1}+\ell_{1,3}\ell_{3,2}\\ \;q_{3,3}^{\left(2\right)}(𝒯)\;\;\;\; & =\ell_{1,3}\ell_{2,3}+\ell_{1,2}\ell_{2,3}+\ell_{2,1}\ell_{1,3}\\ q_{1,1}^{\left(2\right)}({𝒯}^{+})\;\;\;\; & =\ell_{2,1}\ell_{3,1}+\ell_{2,1}\ell_{3,2}+\ell_{2,3}\ell_{3,1}+\ell_{2,1}r+\ell_{2,3}r\\ q_{1,2}^{\left(2\right)}({𝒯}^{+})\;\;\;\; & =\ell_{1,2}\ell_{3,2}+\ell_{1,3}\ell_{3,2}+\ell_{1,2}\ell_{3,1}+\ell_{1,2}r\\ q_{1,3}^{\left(2\right)}({𝒯}^{+})\;\;\;\; & =\ell_{1,3}\ell_{2,3}+\ell_{1,2}\ell_{2,3}+\ell_{1,3}\ell_{2,1}\\ q_{4,4}^{\left(3\right)}({𝒯}^{+})\;\;\;\; & =\ell_{1,3}\ell_{2,3}r+\ell_{1,2}\ell_{2,3}r+\ell_{1,3}\ell_{2,1}r. \end{array} \end{array}$$

The reader may also verify that these expressions are precisely the weights of the spanning forests given in Fig 2B and C. S1 Fig shows the agreement between the mFPT obtained using this procedure and a set of simulated trajectories of $X^{+}(\cdot )$ using the Gillespie algorithm [32].

### Quantifying decoupling

To quantify decoupling between SS(*x*) and mFPT^*i*^(*x*), we consider normalised dynamic ranges for the two outputs, which we define as


$$\begin{array}{c} \overset{―}{SS}(x)=\frac{SS(x)}{r/\delta }\;\text{and}\;{\overset{―}{mFPT}}^{i}(x)=\frac{{mFPT}^{i}(x)}{\max_{x>0}\left\{{mFPT}^{i}(x)\right\}}. \end{array}$$ (15)

These definitions restrict $\overset{―}{SS}(x)$ and ${\overset{―}{mFPT}}^{i}(x)$ to between 0 and 1. Indeed, this restriction is self-evident for ${\overset{―}{mFPT}}^{i}(x)$; for $\overset{―}{SS}(x)$, this stems from Eq. 4, which tells us that


$$\begin{array}{c} \overset{―}{SS}(x)=\sum_{v\in {𝒱}_{\text{prod}}(G)}p_{v}^{*} \end{array}$$

is the sum of steady-state probabilities for the subset of productive states, $v\in {𝒱}_{\text{prod}}(G)$. From here, we define the dynamic range of the two outputs as


$$\begin{array}{cc} \Delta_{\overset{―}{SS}} & =\max_{x>0}\left\{\overset{―}{SS}(x)\right\}-\min_{x>0}\left\{\overset{―}{SS}(x)\right\}\\ \Delta_{{\overset{―}{mFPT}}^{i}} & =\max_{x>0}\left\{{\overset{―}{mFPT}}^{i}(x)\right\}-\min_{x>0}\left\{{\overset{―}{mFPT}}^{i}(x)\right\}, \end{array}$$ (16)

which also range between 0 and 1. We note that both $\Delta_{\overset{―}{SS}}$ and $\Delta_{{\overset{―}{mFPT}}^{i}}$ are relative dynamic ranges: the former is normalised by the theoretical maximum value of SS(*x*) (namely r/δ), while the latter is normalised by the actual maximum value of mFPT^*i*^(*x*). Therefore, both dynamic ranges account for changes in SS(*x*) and mFPT^*i*^(*x*) in proportion to these maximum values. We also note that perfect decoupling, in which only the steady-state level changes with input concentration, is obtained when $\Delta_{{\overset{―}{mFPT}}^{i}}=0$ and $\Delta_{\overset{―}{SS}}=1$.

## Results

### Decoupling under rate scale separation in ${𝒞}_{2}$ and ${𝒟}_{2}$

We start our analysis by considering the two-state chain model, ${𝒞}_{2}$, which is equivalent to the “random telegraph” model of transcriptional bursting (Fig 1E) [14,33,34]. Here, vertex 1 represents an inactive state, and vertex 2 represents an active state that can produce the molecular readout, *M*, so that ${𝒱}_{\text{prod}}({𝒞}_{2})=\left\{2\right\}$. The steady-state level is given by (Eqs. 4 and 9; Appendix A in S1 Text) [22,28]:


$$\begin{array}{c} SS(x)=\frac{rp_{2}^{*}}{\delta }=\frac{r}{\delta }\left(\frac{\ell_{1,2}(x)}{\ell_{1,2}(x)+\ell_{2,1}(x)}\right), \end{array}$$

whereas the activation time is given by (Eq. 10)


$$\begin{array}{c} {mFPT}^{1}(x)=\frac{\ell_{1,2}(x)+\ell_{2,1}(x)+r}{r\ell_{1,2}(x)}, \end{array}$$ (17)

where we have set the initial vertex to *i* = 1. Notice that, upon sending r→∞, we have


$$\begin{array}{c} \lim_{r\to \infty }{mFPT}^{1}(x)=\frac{1}{\ell_{1,2}(x)} \end{array}$$ (18)

From this, we can see that if the ligand acts only on the edge 2→1, such that $\ell_{1,2}(x)=\ell_{1,2}$ does not depend on *x*, and the production rate is large (r→∞), then mFPT^1^(*x*) remains constant while the steady-state level, SS(*x*), changes with *x*. Thus, in this regime, the ligand may only affect the steady-state level, but not the activation time, leading to decoupling. This simple model immediately suggests that decoupling can be easily achieved if the ligand only acts on the deactivation transition, as long as production from vertex 2 is sufficiently fast such that, upon reaching vertex 2, the system produces *M* before transitioning back to vertex 1. We call this *rate scale separation*. If the ligand acts on the edge 1→2 as well, so that $\ell_{1,2}(x)$ also depends on *x*, whether decoupling is possible depends on the functional forms of $\ell_{1,2}(x)$ and $\ell_{2,1}(x)$.

In order to examine the implications of assuming a specific mechanism by which the bound ligand affects the system, we next considered the corresponding ladder model, ${𝒟}_{2}$ (Fig 1F). Here, recalling that ${𝒱}_{\text{prod}}({𝒟}_{2})=\left\{U_{2},B_{2}\right\}$, the steady-state level of *M* is defined as (Eqs. 4 and 9)


$$\begin{array}{c} SS(x)=\frac{r}{\delta }\left(p_{U_{2}}^{*}(x)+p_{B_{2}}^{*}(x)\right), \end{array}$$

and we can similarly use Eq. 10 to derive the activation time, ${mFPT}^{U_{1}}(x)$, with initial vertex *i* = *U*_1_.

The ligand can either promote or hinder production of the readout; in this model, this is determined by the values of the regulatory factors, $\gamma_{1,2}$ and $\gamma_{2,1}$. The former measures the strength with which the ligand regulates the forward transition, $B_{1}\to B_{2}$; the latter measures the strength with which the ligand regulates the backward transition, $B_{2}\to B_{1}$. To simplify our analysis, we will assume that the ligand acts on only one of the two transitions, and that it *promotes* readout production, thus acting as an *activator*. Mathematically, this means that either $\gamma_{1,2}>1$ and $\gamma_{2,1}=1$, or $\gamma_{1,2}=1$ and $\gamma_{2,1}<1$. Using the mathematical machinery laid out in the previous section, it can be shown that, in this regime, SS(*x*) increases monotonically with *x* and ${mFPT}^{U_{1}}(x)$ decreases monotonically with *x* (Appendix B in S1 Text). In this case, analytical expressions for the corresponding dynamic ranges can be obtained by comparing the values of $\overset{―}{SS}(x)$ and ${\overset{―}{mFPT}}^{U_{1}}(x)$ at *x* = 0 and x→∞. In particular, when the forward transition is regulated by the ligand ($\gamma_{1,2}>1$, $\gamma_{2,1}=1$), we obtain the following dynamic ranges:


$$\begin{array}{c} \Delta_{\overset{―}{SS}}=\frac{\gamma_{1,2}-1}{\gamma_{1,2}\left(\frac{\ell_{1,2}}{\ell_{2,1}}\right)+\gamma_{1,2}+1+\frac{\ell_{2,1}}{\ell_{1,2}}},\;\Delta_{{\overset{―}{mFPT}}^{U_{1}}}=\frac{\gamma_{1,2}-1}{\gamma_{1,2}}\left(\frac{\ell_{2,1}+r}{\ell_{1,2}+\ell_{2,1}+r}\right). \end{array}$$ (19)

Meanwhile, when the ligand regulates the backward transition ($\gamma_{1,2}=1$, $\gamma_{2,1}<1$), we obtain,


$$\begin{array}{c} \Delta_{\overset{―}{SS}}=\frac{1-\gamma_{2,1}}{\frac{\ell_{1,2}}{\ell_{2,1}}+\gamma_{2,1}+1+\gamma_{2,1}\left(\frac{\ell_{2,1}}{\ell_{1,2}}\right)},\;\Delta_{{\overset{―}{mFPT}}^{U_{1}}}=\frac{1-\gamma_{2,1}}{\frac{\ell_{1,2}}{\ell_{2,1}}+\gamma_{2,1}+\frac{r}{\ell_{2,1}}}. \end{array}$$ (20)

The expressions in Eq. 20 reveal that, when the backward transition is regulated by the ligand, $\Delta_{{\overset{―}{mFPT}}^{U_{1}}}$ tends to zero as r→∞, while $\Delta_{\overset{―}{SS}}$ does not depend on *r*. However, if the forward transition is regulated by the ligand (Eq. 19), then taking the same limit causes $\Delta_{{\overset{―}{mFPT}}^{U_{1}}}$ to converge to a finite nonzero value, namely $(\gamma_{1,2}-1)/\gamma_{1,2}$. Therefore, a similar result to what we found for ${𝒞}_{2}$ holds for ${𝒟}_{2}$: the two outputs can be decoupled if the ligand regulates the backward transition, $B_{2}\to B_{1}$, and the production rate, *r*, is large. Notice that, in both equations, $\Delta_{\overset{―}{SS}}$ and $\Delta_{{\overset{―}{mFPT}}^{U_{1}}}$ may both tend to zero in other limiting regimes, e.g., $\ell_{1,2}/\ell_{2,1}\to \infty$; this does not correspond to our definition of decoupling, but rather a trivial case of unresponsiveness of the system.

### Decoupling under rate scale separation in ${𝒟}_{3}$

Molecular systems in various biological settings often transition through multiple states before producing the molecular readout [9,14,15,35,36]. We thus asked what happens in the ladder model ${𝒟}_{3}$.

To begin with a simplified setting, we assumed $\ell_{1,2}=\ell_{2,1}$ and $\ell_{2,3}=\ell_{3,2}$, and that the ligand regulates exactly one of the four transitions, $B_{1}\to B_{2}$, $B_{2}\to B_{1}$, $B_{2}\to B_{3}$, and $B_{3}\to B_{2}$, promoting readout production. In this case, there are four possibilities, which are enumerated in Table 1. For each of these regimes, it can be shown that SS(*x*) and ${mFPT}^{U_{1}}(x)$ are both monotonic in *x* (Appendix B in S1 Text) and analytical expressions for the dynamic ranges of the corresponding normalised quantities can be obtained, analogously to the ${𝒟}_{2}$ model.

**Table 1:** Dynamic ranges of $\overset{―}{SS}$ and ${\overset{―}{mFPT}}^{U_{1}}$ in ${𝒟}_{3}$, for regulatory regimes that promote readout production through the regulation of one transition.

regulatory regimes		$\Delta_{\overset{―}{SS}}$	$\Delta_{{\overset{―}{mFPT}}^{U_{1}}}$
1.I	$\gamma_{1,2}>1$	$\frac{\gamma_{1,2}-1}{3\left(2\gamma_{1,2}+1\right)}$	$\frac{\gamma_{1,2}-1}{\gamma_{1,2}}\left(\frac{\beta +\alpha +\alpha \beta }{\alpha \beta +2\beta +3\alpha }\right)$
1.II	$\gamma_{2,1}<1$	$\frac{1-\gamma_{2,1}}{3\left(\gamma_{2,1}+2\right)}$	$\left(1-\gamma_{2,1}\right)\left(\frac{\alpha +\beta }{\alpha \beta +2\beta +3\alpha }\right)$
1.III	$\gamma_{2,3}>1$	$\frac{2\left(\gamma_{2,3}-1\right)}{3\left(\gamma_{2,3}+2\right)}$	$\frac{\gamma_{2,3}-1}{\gamma_{2,3}}\left(\frac{2\left(\alpha +\beta \right)}{\alpha \beta +2\beta +3\alpha }\right)$
1.IV	$\gamma_{3,2}<1$	$\frac{2\left(1-\gamma_{3,2}\right)}{3\left(2\gamma_{3,2}+1\right)}$	$\left(1-\gamma_{3,2}\right)\left(\frac{2\alpha }{\alpha \beta +2\beta +3\alpha }\right)$

The normalised dynamic ranges for the four parametric regimes are summarised in Table 1 below, where we have introduced the dimensionless parameters,


$$\begin{array}{c} \alpha =\frac{\ell_{2,3}}{\ell_{1,2}}\;\text{and}\;\beta =\frac{r}{\ell_{1,2}}. \end{array}$$ (21)

We first notice that, in almost all the cases, having a large production rate (r→∞) is no longer sufficient to get decoupling. The only case in which this is sufficient is case 1.IV, where only the last backward transition, $B_{3}\to B_{2}$, is regulated. Here, we may rewrite $\Delta_{{\overset{―}{mFPT}}^{U_{1}}}$ as,


$$\begin{array}{c} 2\left(1-\gamma_{3,2}\right){\left(\frac{r}{\ell_{1,2}}+\frac{2r}{\ell_{2,3}}+3\right)}^{-1}. \end{array}$$

This is in line with the findings for ${𝒟}_{2}$ in the previous section, and can be understood intuitively as follows. When *r* is sufficiently large, every time the system reaches *U*_3_ or *B*_3_, it will rapidly proceed to *M* without backtracking to vertex *U*_2_ or *B*_2_, respectively. Therefore, the mFPT from *U*_1_ to *M* can be approximated as the mFPT from *U*_1_ to *U*_3_ or *B*_3_, whichever is reached first. Now, if the ligand does not regulate any transition other than $B_{3}\to B_{2}$, the dynamics with which the system proceeds to *U*_3_ or *B*_3_ in the limits of zero or infinite ligand concentration, respectively, are the same on average. Since the mFPT is monotonic in *x* (Appendix B in S1 Text), this implies that the mFPT does not change with *x*. Therefore, in case 1.IV, a large production rate is sufficient for decoupling.

In addition, we notice that for cases 1.II, 1.III and 1.IV, but not 1.I, there exists a different parametric regime in which $\Delta_{{\overset{―}{mFPT}}^{U_{1}}}$ tends to zero but $\Delta_{\overset{―}{SS}}$ does not, thus giving rise to decoupling: α,β≫1, which we may rewrite in terms of rates as $\ell_{2,3},r\gg \ell_{1,2}$. In particular, we note that, in cases 1.II and 1.III, it is not sufficient to have *either* α≫1 *or* β≫1; rather, both α and β must be large. In this rate-scale-separated regime, the first forward transition in the absence of ligand, $U_{1}\to U_{2}$, is much slower than the second, $U_{2}\to U_{3}$, as well as the production transitions, $U_{3}\to M$ and $B_{3}\to M$. In addition, when $\gamma_{1,2}=1$ (as in cases 1.II, 1.III, and 1.IV), the first forward transition in the presence of ligand, $B_{1}\to B_{2}$, is also much slower than $U_{2}\to U_{3}$, $B_{2}\to B_{3}$, $U_{3}\to M$, and $B_{3}\to M$. We hypothesised that, in this case, a partitioning of the graph arises where the slow rate, namely $\ell_{1,2}$, completely determines the activation time, while the fast rates dictate the dynamic range of the steady-state level. To test this hypothesis, we examined ${mFPT}^{U_{1}}(x)$ for cases 1.II, 1.III and 1.IV, when *x* = 0. For all three cases, the activation time is given by (Eq. 13 and setting *x* = 0),


$$\begin{array}{c} {mFPT}^{U_{1}}(x=0)=\frac{1}{\ell_{1,2}}\left(1+\frac{3\alpha +2\beta }{\alpha \beta }\right). \end{array}$$ (22)

Here, imposing α,β≫1 yields an activation time that depends merely on $\ell_{1,2}$. Since, as shown above, $\Delta_{{\overset{―}{mFPT}}^{U_{1}}}\to 0$ in this parametric regime for cases 1.II, 1.III and 1.IV, this means that ${mFPT}^{U_{1}}(x)$ depends merely on $\ell_{1,2}$ for all *x*. Therefore, under rate scale separation, the activation time depends entirely on the *slower, unregulated* forward transition.

Meanwhile, the expression for the steady-state level, SS(*x*), depends on the specific case, as well as which parametric limits are applied to realise the condition that α and β should be large. For instance, in the simple setting in which we send $\ell_{1,2}\to 0$, it can be easily shown that the steady-state level in case 1.III—obtained by applying Eq. 9 and taking the symbolic limit as $\ell_{1,2}\to 0$—depends on the fast rate $\ell_{2,3}$, and the corresponding regulatory factor, $\gamma_{2,3}$, in addition to *k*_on_, *k*_off_, and *x*. In contrast, the steady-state level in case 1.II, in which the slow backward transition $B_{2}\to B_{1}$ is regulated, depends on the regulatory factor, $\gamma_{2,1}$, but not the fast rate, $\ell_{2,3}$.

**Fig 3:**
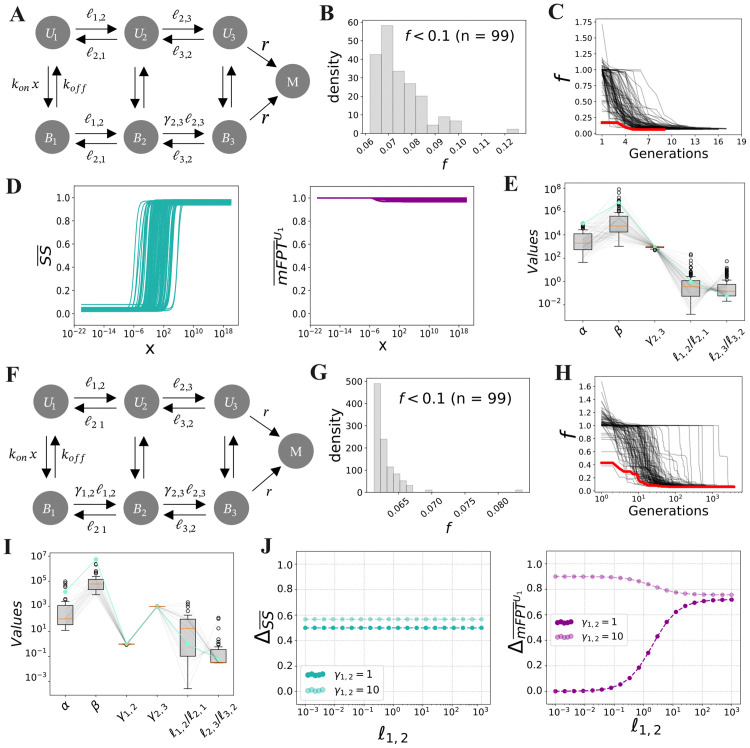
Decoupling under rate scale separation in the ladder model, ${𝒟}_{3}$, for case 1.III in Table 1 (A–E) and a generalization of case 2.I in Table 2 (F–J). **(A)** Schematic of ${𝒟}_{3}$ with regulation of $B_{2}\to B_{3}$ (case 1.III). **(B)** Distribution of coupling scores after termination of the PSO. Each PSO run was terminated whenever *f* < 1 for more than 5 consecutive generations. For almost all the runs (n = 99) *f* < 0.1. **(C)** Evolution of *f* over each PSO run. The red curve represents the “best” optimised parameter set with the smallest value of *f*. **(D)** Input-output responses of optimised parameter sets for which *f* < 1 and the steady-state level increases monotonically with *x*. **(E)** Distributions of parameter values corresponding to the curves in panel D, with $\alpha =\ell_{2,3}/\ell_{1,2}$ and $\beta =r/\ell_{1,2}$. The green curve represents the best parameter set (red curve in **C**). **(F)** Schematic of ${𝒟}_{3}$ with regulation of $B_{1}\to B_{2}$ and $B_{2}\to B_{3}$ (generalization of case 2.I). **(G)** Distribution of coupling scores after termination of the PSO. Each PSO run was terminated after 23 hours of computation time. For almost all the runs (n = 99) *f* < 0.1. **(H)** Evolution of *f* over each PSO run. The red curve represents the best parameter set. **(I)** Distributions of optimised parameter values for which *f* < 1 and the steady-state level increases monotonically with *x*. The green curve corresponds to the best parameter set (red curve in **H**). **(J)** Normalised dynamic ranges for two families of parameter sets, with the parameters set as follows: $\ell_{2,3}=\ell_{3,2}=10\delta$, $k_{\text{off}}=\delta$, $k_{\text{on}}=\delta /\left(1\;\text{c.u.}\right)$, r=10δ, $\gamma_{2,3}=10$, $\gamma_{1,2}=1$ or 10, and $\ell_{1,2}=\ell_{2,1}$ varied over a logarithmic range. The dots represent numerical computations (Materials and Methods), and the dashed lines represent the formulas in Table 2 (case 2.I).

Next, we aimed to assess whether rate scale separation can still give rise to decoupling if we relax some of the assumptions made in the above analysis, namely that $\ell_{1,2}=\ell_{2,1}$ and $\ell_{2,3}=\ell_{3,2}$. We found that, upon relaxing these assumptions, the expressions for $\Delta_{\overset{―}{SS}}$ and $\Delta_{\overset{―}{mFPT}}$ become substantially more complicated; therefore, we resorted to numerical optimisation to search for parameter regimes that lead to decoupling, focusing on case 1.III ($\gamma_{2,3}>1$; Fig 3A).

We defined a *coupling score*,


$$\begin{array}{c} f=1-(\Delta_{\overset{―}{SS}}-\Delta_{{\overset{―}{mFPT}}^{U_{1}}}), \end{array}$$

which describes the extent of coupling between the steady-state level and activation time. Perfect decoupling corresponds to *f* = 0, corresponding to a maximum steady-state dynamic range ($\Delta_{\overset{―}{SS}}=1$) and a minimum activation time dynamic range ($\Delta_{{\overset{―}{mFPT}}^{U_{1}}}=0$). We then searched for parameter sets that yield a small value of *f* by using a Particle Swarm Optimisation (PSO) algorithm [37,38] (Materials and Methods). Briefly, this algorithm begins with a collection (or “swarm”) of parameter sets (or “particles”), and iteratively updates each particle’s position and velocity according to the values of the objective function (here, *f*) across the swarm. Over successive generations, the swarm collectively converges to the optimal solution(s) with respect to the objective function.

We allowed the rates, $\ell_{i,j}$, *k*_off_, and *r*, to lie within a large parameter range, namely $\left[10^{-4},10^{4}\right]$ in units of δ. The binding rate constant, *k*_on_, was also assumed to lie in the range $\left[10^{-4},10^{4}\right]$, but in units of δ/(1c.u.), where “c.u.” denotes the concentration units used for *x*. Finally, the dimensionless regulatory parameter, $\gamma_{2,3}$, was constrained to lie in the range $\left[1,10^{3}\right]$. Each parameter was restricted to lie within these ranges throughout the optimisation. We ran 100 independent optimisation runs, each starting from a different random initial condition. Fig 3B and C show that more than 90% of the runs converged to a final coupling score of *f* < 0.1, strongly suggesting that this procedure effectively minimises the objective function.

Among these successful optimisation runs, we retained only the optimised parameter sets for which $\overset{―}{SS}$ increases with *x*, and visualised the corresponding normalised steady-state level and activation time responses (Fig 3D). This revealed that, while the steady-state level increases monotonically with *x* (Fig 3D, left), the mFPT barely changes (Fig 3D, right), demonstrating near-perfect decoupling. In line with our analytical results, we found that (1) the values of α and β are both large, and (2) the second forward transition, $B_{2}\to B_{3}$, is strongly promoted by the ligand, with $\gamma_{2,3}$ almost always reaching its maximum value of 10^3^ (Fig 3E). This strongly suggests that our optimisation procedure is identifying parameter regimes that achieve decoupling via rate scale separation.

Notably, we also found that $\ell_{3,2}$ often exceeds $\ell_{2,3}$, albeit to an extent less than *r*; for instance, the best parameter set over all optimisation runs (Fig 3E, green) exhibited $\ell_{3,2}/\ell_{2,3}\sim 10$ and $\beta /\alpha =r/\ell_{2,3}\sim 10^{2}$. We also found a broad distribution of values for $\ell_{1,2}/\ell_{2,1}$. This indicates that the assumptions that $\ell_{1,2}=\ell_{2,1}$ and $\ell_{2,3}=\ell_{3,2}$ are not necessary for decoupling via rate scale separation. Indeed, combining these results with the analytical formulas in Table 1 reveals that $\ell_{3,2}>\ell_{2,3}$ *strengthens* decoupling, by increasing the steady-state dynamic range, $\Delta_{\overset{―}{SS}}$. We can understand this effect by considering some simple limiting cases. For instance, it is easy to see that, for a regulatory regime in case 1.III in which $\ell_{1,2}=\ell_{2,1}$, $\ell_{2,3}=\ell_{3,2}$, and $\gamma_{2,3}\gg 1$, we have a basal steady-state response of $\overset{―}{SS}(x=0)=1/3$, which limits the steady-state dynamic range that can be achieved (Appendix C in S1 Text). On the other hand, removing the equality constraints on the horizontal transition rates enables the accumulation of steady-state probability on the non-productive states, *U*_1_ and *U*_2_, and thereby decreases $\overset{―}{SS}(x=0)$, which allows for an increased dynamic range. Therefore, asymmetry in the transition rates acts in concert with the regulatory factor, $\gamma_{2,3}$, to modulate the steady-state dynamic range, whereas α and β together dictate the mFPT dynamic range, thus inducing decoupling.

We next proceeded to extend our analysis to regulatory regimes in which the ligand regulates *two* transitions. There are 16 such possible regulatory regimes, depending on the choice of ligand-regulated transitions and whether each corresponding regulatory factor, $\gamma_{i,j}$, is either greater than or less than 1. We can categorise these regimes into two classes: eight *coherent* regimes, in which the ligand consistently promotes or hinders transitioning towards the productive state (e.g., $\gamma_{1,2}>1$ and $\gamma_{2,3}>1$); and eight *incoherent* regimes, in which the ligand simultaneously promotes and hinders transitioning towards the productive state (e.g., $\gamma_{1,2}>1$ and $\gamma_{2,3}<1$) [24]. We first restricted our attention to the four *coherent* regulatory regimes in which the ligand consistently *promotes* transitioning towards the productive state; these regimes are enumerated in Table 2. For each of these regimes, it can be shown that SS(*x*) and ${mFPT}^{U_{1}}(x)$ increase and decrease monotonically with *x*, respectively (Appendix B in S1 Text), and we can obtain analytical expressions for the normalised dynamic ranges of these quantities, as before. These expressions are given in Table 2.

**Table 2:** Dynamic ranges of $\begin{array}{c} \overset{―}{SS} \end{array}$ and $\begin{array}{c} {\overset{―}{mFPT}}^{U_{1}} \end{array}$ in $\begin{array}{c} D_{3} \end{array}$, for regulatory regimes that promote readout production through the regulation of two transitions.

regulatory regimes		$\Delta_{\overset{―}{SS}}$	$\Delta_{{\overset{―}{mFPT}}^{U_{1}}}$
2.I	$\gamma_{1,2}>1$, $\gamma_{2,3}>1$	$\frac{2\gamma_{1,2}\gamma_{2,3}-\gamma_{1,2}-1}{3\left(\gamma_{1,2}\gamma_{2,3}+\gamma_{1,2}+1\right)}$	$\frac{\alpha \beta \gamma_{2,3}(\gamma_{1,2}-1)+\left(\alpha +\beta \right)\left(2\gamma_{1,2}\gamma_{2,3}-\gamma_{1,2}-1\right)}{\gamma_{1,2}\gamma_{2,3}\left(\alpha \beta +3\alpha +2\beta \right)}$
2.II	$\gamma_{1,2}>1$, $\gamma_{3,2}<1$	$\frac{2\gamma_{1,2}-\gamma_{1,2}\gamma_{3,2}-\gamma_{3,2}}{3(\gamma_{1,2}\gamma_{3,2}+\gamma_{1,2}+\gamma_{3,2})}$	$\frac{\left(\alpha \beta +\beta \right)\left(\gamma_{1,2}-1\right)+\alpha \left(2\gamma_{1,2}-\gamma_{1,2}\gamma_{3,2}-\gamma_{3,2}\right)}{\gamma_{1,2}\left(\alpha \beta +3\alpha +2\beta \right)}$
2.III	$\gamma_{2,1}<1$, $\gamma_{2,3}>1$	$\frac{2\gamma_{2,3}-\gamma_{2,1}-1}{3\left(\gamma_{2,1}+\gamma_{2,3}+1\right)}$	$\frac{\left(\alpha +\beta \right)\left(2\gamma_{2,3}-\gamma_{2,1}-1\right)}{\gamma_{2,3}\left(\alpha \beta +3\alpha +2\beta \right)}$
2.IV	$\gamma_{2,1}<1$, $\gamma_{3,2}<1$	$\frac{2-\gamma_{2,1}\gamma_{3,2}-\gamma_{3,2}}{3\left(\gamma_{2,1}\gamma_{3,2}+\gamma_{3,2}+1\right)}$	$\frac{\beta \left(1-\gamma_{2,1}\right)+\alpha \left(2-\gamma_{2,1}\gamma_{3,2}-\gamma_{3,2}\right)}{\alpha \beta +3\alpha +2\beta }$

Although these expressions are more complicated, we still see that imposing rate scale separation, by setting α,β≫1, causes $\Delta_{{\overset{―}{mFPT}}^{U_{1}}}$, but not $\Delta_{\overset{―}{SS}}$, to tend to zero, as long as the slow, forward transition ($B_{1}\to B_{2}$) is not regulated (cases 2.III and 2.IV). Moreover, for each of these two cases, it is easy to directly compare the expressions for $\Delta_{\overset{―}{SS}}$ and $\Delta_{{\overset{―}{mFPT}}^{U_{1}}}$ to the expressions that arise when only one transition is regulated (cases 1.II and 1.III for 2.III, and cases 1.II and 1.IV for 2.IV), to see that $\Delta_{{\overset{―}{mFPT}}^{U_{1}}}$ decreases and $\Delta_{\overset{―}{SS}}$ increases when a second transition is regulated. As such, introducing a second regulated transition enhances decoupling.

We next assessed the relevance of the constraints $\ell_{1,2}=\ell_{2,1}$ and $\ell_{2,3}=\ell_{3,2}$, again using the numerical optimisation procedure outlined above. We aimed to minimise the coupling score, *f*, for a generalised version of case 2.I (Fig 3F), in which $\gamma_{1,2}$ and $\gamma_{2,3}$ can both assume any value within the range, $\left[10^{-3},10^{3}\right]$. Similarly to the optimisation for case 1.III, we found that more than 90% of the optimisation runs converge to a coupling score of *f* < 0.1 (Fig 3G), albeit with a larger number of generations (Fig 3H), as expected by the increased dimensionality of the parameter space. Notably, we found that the optimal parameter sets exhibit significant rate scale separation, α≫1 and β≫1, as well as values of $\gamma_{1,2}\approx 1$, representing little to no regulation of the slow forward transition $B_{1}\to B_{2}$, and $\gamma_{2,3}$ close to the maximum value of 10^3^ (Fig 3I). This strongly suggests that, to attain decoupling, the algorithm is effectively reducing this generalization of case 2.I to a regulatory regime in case 1.III, in which $B_{1}\to B_{2}$ is unregulated. Therefore, regulation of the slow forward transition, $B_{1}\to B_{2}$, does not improve decoupling when the input also regulates $B_{2}\to B_{3}$. This contrasts with what we observed above for cases 2.III and 2.IV, where regulation of two transitions *does* improve decoupling when $B_{1}\to B_{2}$ is *not* regulated.

In addition, we found that the ratios $\ell_{1,2}/\ell_{2,1}$ and $\ell_{2,3}/\ell_{3,2}$ follow similar distributions as in case 1.III (Fig 3E), with $\ell_{3,2}/\ell_{2,3}\sim 10$ in the best parameter set (Fig 3I, green). This, again, reflects the fact that increasing $\ell_{3,2}/\ell_{2,3}$ decreases $\overset{―}{SS}(x=0)$, and therefore increases $\Delta_{\overset{―}{SS}}$, thus strengthening decoupling.

The equations in Table 1 and Table 2 and the numerical results in Fig 3 show that a large rate scale separation can give rise to decoupling. To assess whether decoupling can be achieved in a more constrained scenario, we examined a family of example parameter sets in case 1.III with $\gamma_{2,3}=10$, $\ell_{2,3}=\ell_{3,2}=r=10\delta$, $k_{\text{off}}=\delta$, and $k_{\text{on}}=\delta /\left(1\;\text{c.u.}\right)$, and plotted $\Delta_{\overset{―}{SS}}$ and $\Delta_{{\overset{―}{mFPT}}^{U_{1}}}$ while varying $\ell_{1,2}=\ell_{2,1}$ over several orders of magnitude (Fig 3J, dark colours). Note that, in this case, α=β. Here, we found that, while $\Delta_{\overset{―}{SS}}$ did not significantly vary with $\ell_{1,2}$, we could decrease $\Delta_{{\overset{―}{mFPT}}^{U_{1}}}$ to as small as 0.1 by setting $\alpha =\beta \approx 10^{1.5}$, suggesting that values of α, β, and $\gamma_{2,3}$ much less than those reported in Fig 3E can also give rise to significant decoupling. Meanwhile, we found that the same family of parameter sets but with $\gamma_{1,2}=10$, which instead fall under case 2.I, did not exhibit significant decoupling for any choice of $\ell_{1,2}=\ell_{2,1}$ (Fig 3J, light colours), consistent with our observations in Fig 3I.

In summary, our numerical and analytical results demonstrate that rate scale separation, when paired with a lack of regulation of the slower forward transition, $B_{1}\to B_{2}$, gives rise to decoupling in the ladder model, ${𝒟}_{3}$. In order to check whether this same mechanism enables decoupling in larger models, we performed a similar analysis of ${𝒟}_{6}$ and found that, there too, decoupling can arise when (1) the system exhibits a block of transitions that are slower than a subsequent block of transitions, and (2) regulation occurs along one or more of the faster forward transitions (S9A–C Fig). Such separations of timescales have been widely recognised in the setting of gene regulation, where changes in chromatin state are typically measured to proceed on slower timescales than TF binding and the reactions that comprise the polymerase cycle [33,39–41]. This suggests that decoupling in gene regulation could arise when activating TFs do not accelerate the slow chromatin opening transitions, but rather work on other subsequent steps.

### Decoupling due to incoherent regulation, in the absence of rate scale separation

We then asked whether there are alternative regulatory mechanisms that can yield decoupling when all transitions operate on similar timescales. Recently, we and others have argued that TFs may act on multiple steps of a gene-regulatory mechanism in an incoherent fashion, simultaneously promoting and hindering transcription [22,24]. In the light of this, we hypothesised that such incoherent regulation may be an alternative way to maintain a constant activation time, perhaps by counterbalancing the effects of promoting progression towards the productive state through certain transitions by hindering this progression along other transitions, all while allowing for a change in the steady state.

To examine how decoupling might arise when the transition rates are constrained to be similar, we first considered the extreme scenario in which $\ell_{1,2}=\ell_{2,1}=\ell_{2,3}=\ell_{3,2}$. In this case, assuming that the ligand acts only on the forward transitions $B_{1}\to B_{2}$ and $B_{2}\to B_{3}$ (i.e., $\gamma_{1,2},\gamma_{2,3}\neq 1$ and $\gamma_{2,1}=\gamma_{3,2}=1$), it can be shown that decoupling cannot be achieved in any coherent regulatory regime in which $\gamma_{1,2}\geq 1$ and $\gamma_{2,3}\geq 1$. In particular, it can be shown that, in this regime (Appendix D in S1 Text),


$$\begin{array}{c} \Delta_{\overset{―}{SS}}<\Delta_{{\overset{―}{mFPT}}^{U_{1}}}. \end{array}$$

This implies that, whenever $\gamma_{1,2}\geq 1$ and $\gamma_{2,3}\geq 1$, the coupling score, *f*, must be greater than one. Therefore, if the ligand is assumed to promote one of the forward transitions *and* $\ell_{1,2}=\ell_{2,1}=\ell_{2,3}=\ell_{3,2}$, decoupling may only be achieved if the ligand *hinders* the other forward transition, i.e., the ligand regulates the two transitions in an incoherent manner.

We next considered what happens when we allow for such incoherent regulation by the ligand. Here, the ligand regulates two transitions in such a way that it promotes progression towards the productive state through one transition, while hindering this progression through the other (e.g., $\gamma_{1,2}>1$ and $\gamma_{2,3}<1$). In this case, $\overset{―}{SS}(x)$ and ${\overset{―}{mFPT}}^{U_{1}}(x)$ are not necessarily monotonic in *x* (Appendix B in S1 Text), consistent with similar observations reported in previous work [23,24]. However, we can still characterise conditions under which ${\overset{―}{mFPT}}^{U_{1}}(x)$ is not necessarily constant in *x*, but satisfies the weaker condition,


$$\begin{array}{c} {\overset{―}{mFPT}}^{U_{1}}(x=0)=\lim_{x\to \infty }{\overset{―}{mFPT}}^{U_{1}}(x). \end{array}$$ (23)

In particular, we can show that, if we set $\ell_{1,2}=\ell_{2,3}=\ell_{2,1}=\ell_{3,2}=r$ and allow for regulation along $B_{1}\to B_{2}$ and $B_{2}\to B_{3}$ ($\gamma_{1,2},\gamma_{2,3}\neq 1$ and $\gamma_{2,1}=\gamma_{3,2}=1$), then we have,


$$\begin{array}{c} \lim_{x\to \infty }{\overset{―}{mFPT}}^{U_{1}}(x)-{\overset{―}{mFPT}}^{U_{1}}(x=0)=\frac{2\gamma_{1,2}-5\gamma_{1,2}\gamma_{2,3}+\gamma_{2,3}+2}{6\gamma_{1,2}\gamma_{2,3}}. \end{array}$$

Equating this to zero and solving for either $\gamma_{1,2}$ or $\gamma_{2,3}$, we obtain,


$$\begin{array}{c} \gamma_{1,2}=\frac{\gamma_{2,3}+2}{5\gamma_{2,3}-2}\;\text{and}\;\gamma_{2,3}=\frac{2\left(\gamma_{1,2}+1\right)}{5\gamma_{1,2}-1}. \end{array}$$

From here, it is easy to see that $\gamma_{1,2}>1$ if, and only if, $\gamma_{2,3}<1$. This implies that any coherent regulatory regime in which $\gamma_{1,2},\gamma_{2,3}>1$ or $\gamma_{1,2},\gamma_{2,3}<1$ cannot satisfy Eq. 23, and therefore cannot exhibit a constant ${\overset{―}{mFPT}}^{U_{1}}(x)$ in *x*. This demonstrates that, in the extreme scenario where $\ell_{1,2}=\ell_{2,1}=\ell_{2,3}=\ell_{3,2}=r$, incoherent regulation is necessary to achieve ${\overset{―}{mFPT}}^{U_{1}}(x=0)=\lim_{x\to \infty }{\overset{―}{mFPT}}^{U_{1}}(x)$, which is a prerequisite for a flat activation time, $\Delta_{{\overset{―}{mFPT}}^{U_{1}}}=0$. We emphasise, however, that ${\overset{―}{mFPT}}^{U_{1}}(x)$ may be non-monotonic in *x* in general, in which case $\Delta_{{\overset{―}{mFPT}}^{U_{1}}}$ may be nonzero even if the initial and final values of ${\overset{―}{mFPT}}^{U_{1}}(x)$ are the same.

To pursue a more comprehensive analysis of decoupling in the situation where the transitions proceed with similar rates, we again turned to numerical optimisation. In particular, we adapted our PSO approach to incorporate a “Rate Scale Constraint” (RSC) that constrains the ratio between each pair of transition rates, as follows:


$$\begin{array}{cc} \text{minimize}\;f & =1-\left(\Delta_{\overset{―}{SS}}-\Delta_{{\overset{―}{mFPT}}^{U_{1}}}\right)\\ \text{subject to}\;g_{1} & =\left|\log_{10}\left(\ell_{1,2}/\ell_{2,3}\right)\right|-RSC\leq 0\\ g_{2} & =\left|\log_{10}\left(\ell_{1,2}/\ell_{2,1}\right)\right|-RSC\leq 0\\ g_{3} & =\left|\log_{10}\left(\ell_{2,3}/\ell_{3,2}\right)\right|-RSC\leq 0, \end{array}$$ (24)

where RSC is some positive constant. The smaller RSC is, the more similar $\ell_{1,2}$, $\ell_{2,1}$, $\ell_{2,3}$, and $\ell_{3,2}$ tend to be. Within the PyMoo optimisation framework that we used to perform PSO [38], inequality constraints are handled as penalties to the objective function; as such, we independently confirmed that all solutions obtained from the PSO do satisfy the constraints given in Eq. 24 (S2C Fig, left). We emphasise that we did not impose any constraints on the ligand’s regulatory mode, in principle allowing for both coherent and incoherent regulation.

We first focused on the case where the ligand may regulate the two forward transitions, $B_{1}\to B_{2}$ and $B_{2}\to B_{3}$ (so that $\gamma_{1,2}$ and/or $\gamma_{2,3}$ may be distinct from 1, and $\gamma_{2,1}=\gamma_{3,2}=1$). We ran PSO with six different values for RSC: 0.005, 0.05, 0.5, 1, 2, and 3. As before, to increase the probability of finding global optima, we performed 100 replicates of the optimisation, each starting from a different random initial condition. The convergence of each replicate for RSC = 0.005 is shown in S2A Fig. Consistent with the analyses throughout the manuscript, we considered only activating responses, where the steady-state response increases with TF concentration.

**Fig 4:**
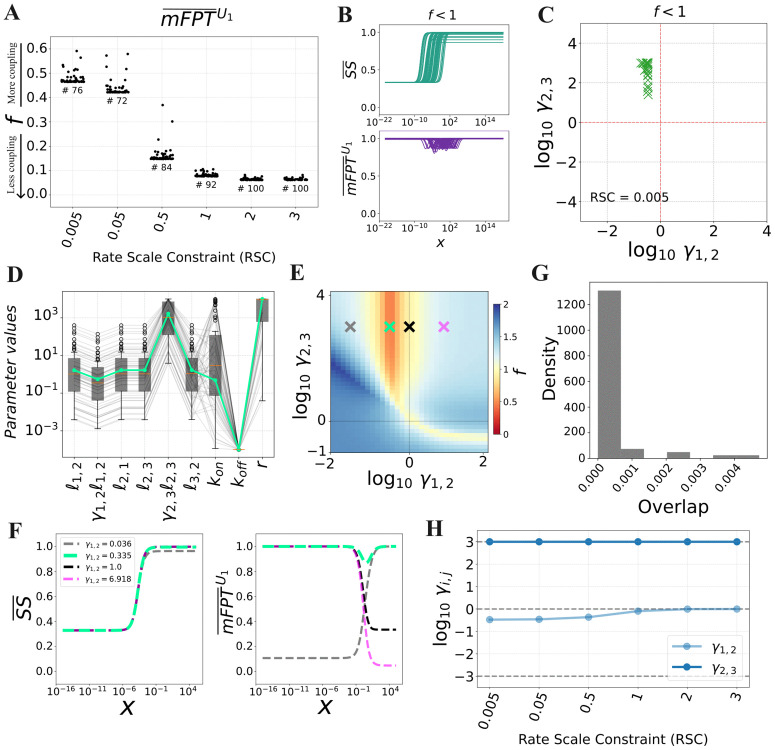
Decoupling under rate scale constraint arises under incoherent regulation. **(A)** Distributions of the coupling score, *f*, obtained from optimisation with various rate scale constraints (RSC). The lower the RSC, the more similar the horizontal transition rates are forced to be. Optimisations were terminated after a predefined compute time (Materials and Methods). Only the parameter sets for which *f* < 1 are shown, and their number for each RSC value is given underneath the corresponding set of points. **(B)** Input-output responses corresponding to the parameter sets obtained from optimisation with RSC = 0.005, for which *f* < 1 and $\overset{―}{SS}$ increases with *x*. **(C)** Values of $\gamma_{1,2}$ and $\gamma_{2,3}$ in the parameter sets corresponding to the responses in **B** (RSC = 0.005). **(D)** Parameter values corresponding to the responses in **B** (RSC = 0.005). The green line represents the best parameter set (minimum *f*). Each parameter is plotted in units of δ, except for *k*_on_, which is in units of δ/(1c.u.). **(E)** Heatmap of the coupling score, *f*, with respect to $\gamma_{1,2}$ and $\gamma_{2,3}$, with the other parameters set to the most optimal parameter set (green curve in **D**), along with select choices of $\gamma_{1,2}$ and $\gamma_{2,3}$ (crosses, *γ*_2,3_ = 10^3^) whose corresponding input-output curves are shown in **F**. **(F)** Input-output curves corresponding to the parameter sets indicated in **E**. **(G)** Overlap between the concentration ranges over which the input-output curves in **B** change by 90%. Given two intervals [*a*,*b*] and [*c*,*d*], the overlap is computed as max{0,min{b,d}−max{a,c}}/((b−a)+(d−c)). The closer this value is to zero, the less overlap there is between the concentration ranges over which $\overset{―}{SS}(x)$ and ${\overset{―}{mFPT}}^{U_{1}}(x)$ exhibit the greatest change. **(H)** Values of $\gamma_{1,2}$ and $\gamma_{2,3}$ in the best parameter set for each choice of RSC.

Fig 4A shows that, as RSC→0, we obtained a larger minimum coupling score, suggesting that it is more difficult to obtain decoupling under rate scale constraint. When examining the corresponding input-output curves for RSC = 0.005 in which $\overset{―}{SS}$ increases with *x*, we observed some dependence of the normalised mFPT on the ligand concentration (Fig 4B, bottom). Yet, we observed that the increase in the coupling score was mostly determined by a smaller dynamic range in the steady-state level, arising from a nonzero basal steady-state level at zero input concentration (Fig 4B, top). This is consistent with our previous reasoning: the closer $\ell_{1,2}/\ell_{2,1}$ and $\ell_{2,3}/\ell_{3,2}$ are to 1, the closer the normalised steady-state level at zero ligand concentration is to 1/3, which is indeed the value of $\overset{―}{SS}(x=0)$ that we observe in our optimisation results (Fig 4B, top).

When examining the optimal parameter sets with a coupling score of *f* < 1 for RSC = 0.005, we found that $\gamma_{1,2}$ and $\gamma_{2,3}$ lie in the incoherent space, with $\gamma_{1,2}<1$ and $\gamma_{2,3}>1$ (Fig 4C). To confirm the relevance of the incoherent regulatory mode, we identified the best parameter set from this ensemble of optimisation results (Fig 4D, green curve) and computed the coupling score while varying $\gamma_{1,2}$ and $\gamma_{2,3}$ within the range $\left[10^{-6},10^{6}\right]$ (Fig 4E shows a portion of this parameter space region; the green cross corresponds to the best parameter set). As expected, in this scenario, moving $\gamma_{1,2}$ away from the identified minimum towards $\gamma_{1,2}=1$ leads to a rapid increase in the coupling score, suggesting significant sensitivity of the coupling score to the value of $\gamma_{1,2}$. To observe this more directly, we computed the responses for the parameter points corresponding to the crosses in Fig 4E (Fig 4F). When we set $\gamma_{1,2}=1$ (black cross), we observed that decoupling was significantly reduced, with $\Delta_{{\overset{―}{mFPT}}^{U_{1}}}\approx \Delta_{\overset{―}{SS}}$; and when we entered the coherent space by setting $\gamma_{1,2}>1$ (pink cross), we found that $\Delta_{{\overset{―}{mFPT}}^{U_{1}}}$ exceeds $\Delta_{\overset{―}{SS}}$ (Fig 4F).

By analysing the curves in Fig 4B, we found that the concentration range over which the steady-state level changed the most was systematically different from that for the mFPT, which can be quantified by the overlap of the input ranges over which the curves change the most (Fig 4G, see also the green curves in Fig 4F). Examining the parameter sets, we noticed that the concentration, *x*_1/2_, at which the steady-state level is half-maximal, i.e.,


$$\begin{array}{c} \frac{\overset{―}{SS}(x_{1/2})-\min\overset{―}{SS}(x)}{\max\overset{―}{SS}(x)-\min\overset{―}{SS}(x)}=\frac{1}{2}, \end{array}$$

is always close to $k_{\text{off}}/k_{\text{on}}$, which is reminiscent of a Michaelis–Menten kinetic scheme (S3A Fig, left). We also observed that the concentration at which the normalised mFPT is minimised, which we denote by *x*_fast_, is close to $\ell_{1,2}/k_{\text{on}}$ (S3A Fig, right). Together, this suggests that we can modulate the overlap to some extent by tuning *k*_off_. Indeed, we found that increasing the value of *k*_off_ in the best parameter set in Fig 4D shifted the normalised steady-state curve rightward and increased *x*_1/2_, while only minimally affecting *x*_fast_ (S3B Fig). However, we also found that increasing *k*_off_ beyond a certain critical value also increases $\Delta_{{\overset{―}{mFPT}}^{U_{1}}}$ (S3B-C Fig). This illustrates that, within an appropriate range of values of *k*_off_, we not only achieve global decoupling in the sense that the variation in the steady-state is much larger than that of the activation time, but we can also achieve a concentration-dependent form of decoupling, in which $\overset{―}{SS}(x)$ and ${\overset{―}{mFPT}}^{U_{1}}(x)$ both vary with *x*, but over largely non-overlapping ranges.

Regarding the effect of the RSC value and thus the similarity of the rates of the various transitions, we found that, as we increased RSC to allow for rate scale separation (RSC ≥ 1), the optimal value of $\gamma_{1,2}$ approached 1, whereas the optimal value of $\gamma_{2,3}$ remained similarly large (Fig 4H). In other words, we observed a transition from an incoherent regime, in which $\gamma_{1,2}<1$, $\gamma_{2,3}\gg 1$, and the transition rates are more tightly constrained, to a regime in which $B_{1}\to B_{2}$ is unregulated ($\gamma_{1,2}\approx 1$), $\gamma_{2,3}\gg 1$, and the transition rates are separated (i.e., case 1.III in Table 1, Fig 3E).

We also noticed that the optimisations tended to yield values of *r* near the maximum possible value (*r* ≈ 10^4^, Fig 4D), although there was a spread of values. To ascertain whether a large value of *r* is necessary for decoupling in this context, we also ran optimisations with the additional constraints that $\ell_{1,2}=\ell_{2,3}=r$ and $\ell_{2,1}=\ell_{3,2}$, so that the production transition proceeds on the same timescale as the preceding forward transitions, $U_{1}\to U_{2}$ and $U_{2}\to U_{3}$ (S4 Fig). As before, we observed the strongest decoupling when the ligand operates in an incoherent regime, with comparably low coupling scores as in the previous optimisation (S4 Fig). This suggests that decoupling due to incoherent regulation does not require a large value of *r* relative to the other transition rates.

Finally, we also considered regulatory regimes in which one or both of the backward transitions, $B_{2}\to B_{1}$ and $B_{3}\to B_{2}$, are regulated. Specifically, we performed additional optimisations with the constraints $\ell_{1,2}=\ell_{2,3}=r$ and $\ell_{2,1}=\ell_{3,2}$, but with the following choices of regulatory factors that may differ from 1: (I) $\gamma_{2,1}$ and/or $\gamma_{2,3}$; (II) $\gamma_{1,2}$ and/or $\gamma_{3,2}$; or (III) $\gamma_{2,1}$ and/or $\gamma_{3,2}$ (S6 Fig, S7 Fig, and S8 Fig, respectively; Appendix E in S1 Text). Here, we found instances of decoupling for cases II and III in the incoherent space ($\gamma_{1,2}<1$ and $\gamma_{3,2}<1$ for case II, $\gamma_{2,1}>1$ and $\gamma_{3,2}<1$ for case III), in which the ligand-bound transitions were biased towards *B*_1_ and *B*_3_. This again demonstrates that incoherent regulation can give rise to decoupling. On the other hand, no instances of decoupling were found for case I, for which the reasons remain elusive (S6 Fig).

In summary, our findings demonstrate that, when the transition rates are similar to each other, incoherent regulation can facilitate decoupling between the steady-state level and activation time. In this regime, unlike decoupling due to rate scale separation, the activation time exhibits a more significant dependence on the input concentration, but this variation can occur over a concentration range that is largely non-overlapping with that over which the steady-state changes (Fig 4G), effectively leading to decoupling. We have also confirmed that these results extend to the larger graph, ${𝒟}_{6}$ (S9D-E Fig).

### Decoupling from an equilibrium of initial states

So far, we have defined the activation time in the ladder models, ${𝒟}_{N}^{+}$, as the mFPT from one initial state, *U*_1_, to the terminal state, *M*, in which a copy of the readout *M* has been produced. This definition is reasonable in the setting of morphogen-mediated gene regulation in developmental systems such as the *Drosophila* blastoderm, a key model system that motivated our analyses in this paper [9,11,14,16]. In this system, the nuclei divide every few minutes, with each mitosis resulting in the repression of transcription and the condensation of chromatin into a broadly inaccessible state. Therefore, it is reasonable in this context to assume that, upon the initiation of a new nuclear cycle, the regulatory DNA element that binds the morphogen has been “reset” to exist in the state *U*_1_.

**Fig 5:**
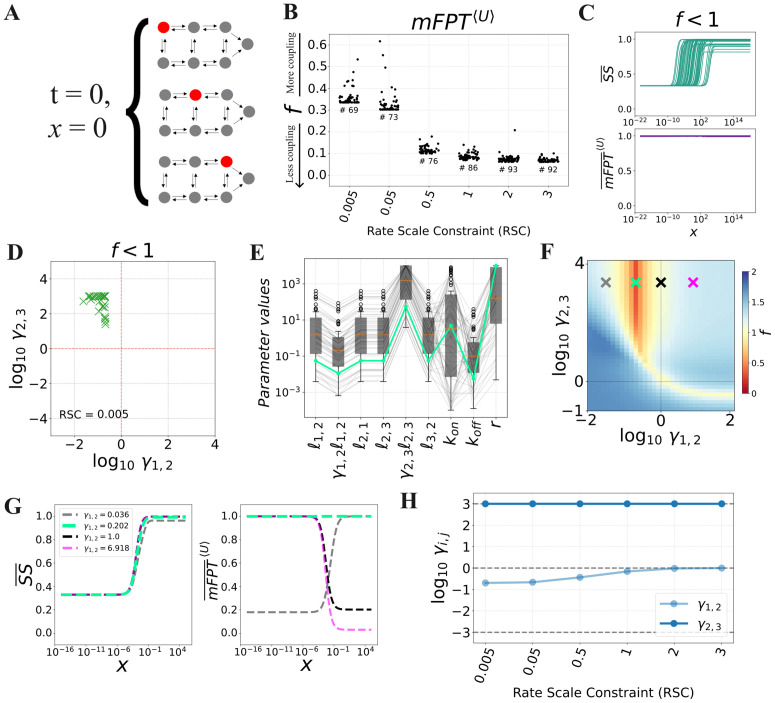
Decoupling from an equilibrium of initial states. **(A)** Schematic of the ${mFPT}^{\langle U\rangle }(x)$ definition of activation time. This definition assumes that, before the ligand is introduced, the system has reached a steady state over the unbound states. That is, at the moment immediately prior to the introduction of ligand (t = 0 and *x* = 0), the system may occupy any of the unbound states (red vertices), each according to its steady-state probability. **(B)** Distributions of the coupling score, *f*, obtained from optimisation with various rate scale constraints (RSC). Only the parameter sets for which *f* < 1 are shown, and their number for each RSC value is given underneath the corresponding set of points. **(C)** Input-output responses corresponding to the parameter sets obtained from optimisation with RSC = 0.005, for which *f* < 1 and $\overset{―}{SS}$ increases with *x*. **(D)** Values of $\gamma_{1,2}$ and $\gamma_{2,3}$ in the parameter sets corresponding to the responses in **C** (RSC = 0.005). **(E)** Parameter values corresponding to the responses in **C** (RSC = 0.005). The green line represents the best parameter set. Each parameter is plotted in units of δ, except for *k*_on_, which is in units of δ/(1c.u.). **(F)** Heatmap of the coupling score, *f*, with respect to $\gamma_{1,2}$ and $\gamma_{2,3}$, with the other parameters set to the most optimal parameter set (green curve in **E**), along with select choices of $\gamma_{1,2}$ and $\gamma_{2,3}$ (crosses, *γ*_2,3_ = 10^3^) whose corresponding input-output curves are shown in **G**. **(G)** Input-output curves corresponding to the parameter sets indicated in **F**. **(H)** Values of $\gamma_{1,2}$ and $\gamma_{2,3}$ in the best parameter set for each choice of RSC.

However, in other contexts, such as those in which the cell is non-dividing or exhibits a long division time, it is plausible that the system exists in an equilibrium of initial states before the ligand is introduced. In this case, a more appropriate measure of the activation time would be the average mFPT to the terminal state over all possible initial states, each weighted by its steady-state probability (Fig 5A). In other words, we define,


$$\begin{array}{c} {mFPT}^{\langle U\rangle }(x)=\sum_{i=1}^{N}\left(p_{U_{i}}^{*}(x=0)\cdot {mFPT}^{U_{i}\;}(x)\right), \end{array}$$ (25)

where we have used the same notation as in Eq. 7, and $p_{U_{i}}^{*}(x=0)$ is the steady-state probability of *U*_*i*_ in ${𝒟}_{N}$ prior to introduction of ligand (*x* = 0). A mathematical justification of this definition is provided in Appendix F in S1 Text.

To determine whether decoupling can be obtained with this alternative definition of activation time, we ran the constrained PSO with different choices of RSC (Eq. 24), again focusing on the case where $\gamma_{1,2}$ and $\gamma_{2,3}$ may be distinct from 1, and $\gamma_{2,1}=\gamma_{3,2}=1$. As with the preceding analysis, we found that the strength of decoupling decreases as we decrease the RSC (corresponding to a stronger constraint on the transition rates), but we still observed significant decoupling at RSC = 0.005 (Fig 5B). As before, the increase in the coupling score with the RSC can be attributed to a smaller dynamic range in the steady-state level (Fig 5C, top). In this case, we found that the activation time shows little to no dependence on the ligand concentration, in contrast to our previous analysis (Fig 5C, bottom; compare to Fig 4B, bottom). We confirmed this observation with Gillespie simulations, which we performed to further validate the definition in Eq. 25 (S5 Fig).

Importantly, the optimised parameter sets were found to lie in the incoherent space for RSC = 0.005 (Fig 5D). Upon varying the regulatory factors, $\gamma_{1,2}$ and $\gamma_{2,3}$, in the best parameter set (Fig 5E, green curve), we again found that the strongest decoupling is indeed obtained when $\gamma_{1,2}<1$ and $\gamma_{2,3}>1$ (Fig 5F and G). Moreover, increasing RSC results in optimal parameter sets that satisfy $\gamma_{1,2}\approx 1$ and $\gamma_{2,3}>1$ (Fig 5H), again demonstrating that, as we allow for rate scale separation, we reach regulatory regimes in which only $B_{2}\to B_{3}$ is regulated (case 1.III in Table 1). Finally, we confirmed that decoupling under incoherent regulation with respect to ${\overset{―}{mFPT}}^{\langle U\rangle }$ can also be observed in the ${𝒟}_{6}$ model (S9F–G Fig).

### Sensitivity of the proposed mechanisms to single-parameter variations

Our analyses demonstrate that decoupling can arise from two mechanisms: rate scale separation and incoherent regulation under rate scale constraint. We now examine how sensitive these mechanisms are to variations in individual parameters. For each optimal parameter set, $\theta^{*}$, with steady-state response $\Delta_{\overset{―}{SS}}(\theta^{*})$ and activation time $\Delta_{{\overset{―}{mFPT}}^{U_{1}}}(\theta^{*})$, we generated a perturbed parameter set, $\theta^{\left(i\right)}$, in which the *i*-th entry was perturbed by setting its value to $10^{\alpha }$, where α was randomly sampled from the uniform distribution on $\left[\log_{10}\left(\theta_{i}^{*}\right)-1,\log_{10}\left(\theta_{i}^{*}\right)+1\right]$, and every other entry was held fixed. We then calculated the corresponding steady-state dynamic range, $\Delta_{\overset{―}{SS}}(\theta^{\left(i\right)})$, and activation time dynamic range, $\Delta_{{\overset{―}{mFPT}}^{U_{1}}}(\theta^{\left(i\right)})$, and compared these values with their unperturbed counterparts, as


$$\begin{array}{cc} \Delta \Delta_{\overset{―}{SS}} & =\Delta_{\overset{―}{SS}}(\theta^{\left(i\right)})-\Delta_{\overset{―}{SS}}(\theta^{*})\\ \Delta \Delta_{{\overset{―}{mFPT}}^{U_{1}}} & =\Delta_{{\overset{―}{mFPT}}^{U_{1}}}(\theta^{\left(i\right)})-\Delta_{{\overset{―}{mFPT}}^{U_{1}}}(\theta^{*}). \end{array}$$ (26)

We emphasise that, in this procedure, each parameter is perturbed one at a time; as such, this analysis does not reveal how the system may respond to simultaneous perturbations of multiple parameters. We expand on this point in the Discussion.

**Fig 6:**
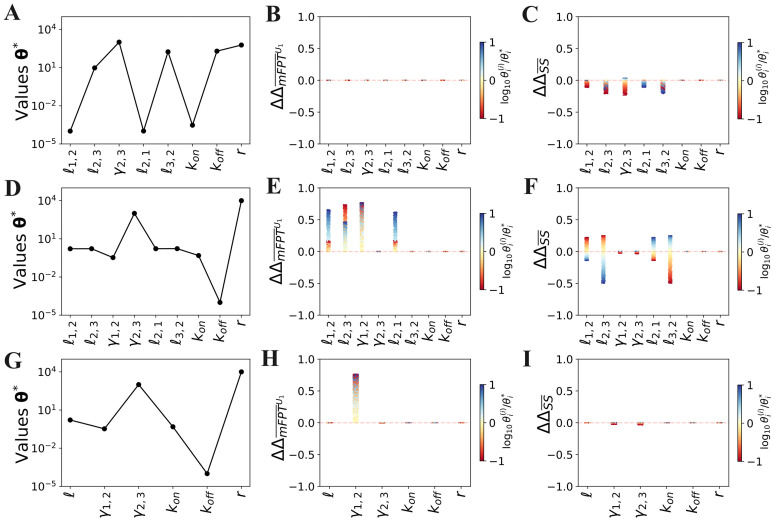
Sensitivity of decoupling via the rate scale separation (A–C) and incoherent regulation (D–I) mechanisms to single-parameter perturbations. **(A)** The best parameter set, $\theta^{*}$, obtained from the optimisation in Fig 3A–E (see also Fig 3E, green line). **(B–C)** Distributions of $\Delta \Delta_{{\overset{―}{mFPT}}^{U_{1}}}$ (**B**) and $\Delta \Delta_{\overset{―}{SS}}$ (**C**) arising from perturbations in each parameter, starting from the choice of $\theta^{*}$ in **A**. The colormap represents the log-ratio of the perturbed parameter value, $\theta_{i}^{\left(i\right)}$, with respect to the optimal parameter value, $\theta_{i}^{*}$. **(D)** The best parameter set, $\theta^{*}$, obtained from the optimisation in Fig 4 (see also Fig 4D, green line). **(E–F)** Distributions of $\Delta \Delta_{{\overset{―}{mFPT}}^{U_{1}}}$ (**E**) and $\Delta \Delta_{\overset{―}{SS}}$ (**F**) arising from perturbations in each parameter, starting from the choice of $\theta^{*}$ in **D**. **(G)** A modified version of the parameter set in **D**, in which all horizontal rates have been set to the same value, $\ell_{1,2}=\ell_{2,1}=\ell_{2,3}=\ell_{3,2}=\ell$, as described in the text. **(H–I)** Distributions of $\Delta \Delta_{{\overset{―}{mFPT}}^{U_{1}}}$ (**H**) and $\Delta \Delta_{\overset{―}{SS}}$ (**I**) arising from perturbations in each parameter, starting from the choice of $\theta^{*}$ in **G**. Here, the horizontal rates were perturbed as a single parameter, *ℓ*, as described in the text.

Fig 6A–6C show the results of this analysis for the rate scale separation mechanism. Here, $\theta^{*}$ was set to the best parameter set in Fig 3E (green line), which is an example of a case 1.III mechanism, in which the ligand regulates the $B_{2}\to B_{3}$ transition via the regulatory factor $\gamma_{2,3}$ (Fig 3A). This parameter set is shown in Fig 6A. For each parameter *i*, we sampled 1000 parameter sets $\theta^{\left(i\right)}$ and calculated the corresponding distributions of $\Delta \Delta_{{\overset{―}{mFPT}}^{U_{1}}}$ (Fig 6B) and $\Delta \Delta_{\overset{―}{SS}}$ (Fig 6C). As shown in Fig 6B, we found that $\Delta_{{\overset{―}{mFPT}}^{U_{1}}}$ is minimally affected by these perturbations. This suggests that, as long as the perturbation does not significantly decrease the rate scale separation, $\Delta_{{\overset{―}{mFPT}}^{U_{1}}}$ is minimally affected. As for $\Delta_{\overset{―}{SS}}$, we found that the same parameter perturbations led to small changes in $\Delta_{\overset{―}{SS}}$, with the largest changes arising from the second set of transitions (Fig 6C). In particular, we note that increasing (resp. decreasing) $\gamma_{2,3}$ leads to an increase (resp. decrease) in $\Delta_{\overset{―}{SS}}$. This was previously suggested by our theoretical analysis of the case 1.III mechanism (Table 1), although we note that this preceding analysis enforced the additional assumption that $\ell_{1,2}=\ell_{2,1}$ and $\ell_{2,3}=\ell_{3,2}$.

In Fig 6D–F, we show the results of two similar analyses for the incoherent regulation mechanism. Here, $\theta^{*}$ was first set to the best parameter set in Fig 4D (green line), in which the horizontal transition rates were tightly constrained, with an RSC value of 0.005; this parameter set is shown in Fig 6D. Now, since introducing parameter perturbations can cause these constraints to be violated, and incoherent regulation is only useful for decoupling for tightly constrained rates (Fig 4H), we hypothesised that perturbing the horizontal transition rates in $\theta^{*}$ would lead to weaker decoupling. This is indeed what we observed: perturbing $\ell_{1,2}$, $\ell_{2,3}$, or $\ell_{2,1}$ in either direction resulted in significantly larger values of $\Delta_{{\overset{―}{mFPT}}^{U_{1}}}$ (Fig 6E). In contrast, we observed no such effect upon perturbing $\ell_{3,2}$; we attribute this to the significantly larger value of *r* in $\theta^{*}$ (Fig 6D), which is sufficiently large that a 10-fold increase in $\ell_{3,2}$ does not appreciably increase the probabilities of the backward transitions, $U_{3}\to U_{2}$ and $B_{3}\to B_{2}$, relative to the probabilities of the terminal transitions, $U_{3}\to M$ and $B_{3}\to M$. Moreover, we found that perturbing the regulatory parameter, $\gamma_{1,2}$, also gives rise to increased $\Delta_{{\overset{―}{mFPT}}^{U_{1}}}$, but perturbing $\gamma_{2,3}$ does not; this is consistent with what we observed in Fig 4E, where a thin sliver of values in $\gamma_{1,2}$ in the incoherent space affords the strongest decoupling under strong rate scale constraint. As for $\Delta_{\overset{―}{SS}}$, we found that its sensitivity to parameter perturbations is comparable to that for $\Delta_{{\overset{―}{mFPT}}^{U_{1}}}$, with the largest changes arising from perturbations to the horizontal transition rates (Fig 6F).

We then sought to understand whether the incoherent regulation mechanism is sensitive to a different type of perturbation, in which we force all horizontal rates to be equal (Fig 6G–I). Here, we set $\theta^{*}$ to the same optimal parameter set as in Fig 4D, but with $\ell_{2,1}$, $\ell_{2,3}$, and $\ell_{3,2}$ set to the $\ell_{1,2}$ value. This modified parameter set is shown in Fig 6G. We then treated all four of these parameters as one common parameter, denoted *ℓ*, which we perturbed in the same way as before. Here, since perturbing *ℓ* does not change the ratios between the horizontal rates, which are fixed at 1, we hypothesised that the decoupling mechanism corresponding to $\theta^{*}$ is more robust to such perturbations. Again, this is indeed what we observed (Fig 6H). We also observed a similarly high sensitivity to perturbations in $\gamma_{1,2}$ and low sensitivity to perturbations in $\gamma_{2,3}$ as in the preceding analysis. Finally, we found that perturbing *ℓ* also leads to negligible changes in $\Delta_{\overset{―}{SS}}$ (Fig 6I), in contrast to the sensitivity of $\Delta_{\overset{―}{SS}}$ to perturbations in the horizontal transition rates in Fig 6F. This is consistent with our observations in Appendix C in S1 Text, on the effects of variable horizontal rates on $\Delta_{\overset{―}{SS}}$. In all, these results demonstrate that rate scale separation is less sensitive to single-parameter variations than incoherent regulation with a rate scale constraint, due to the intrinsic requirement that the horizontal rates be similar in the latter mechanism.

In S10 Fig, we show the results of similar analyses for two parameter sets identified from the optimisations in Fig 5, which were performed with respect to the ${mFPT}^{\langle U\rangle }$ definition of activation time (Eq. 25). As described next, these analyses yielded very similar results to those shown in Fig 6, corresponding to the ${mFPT}^{U_{1}}$ definition.

First, we set $\theta^{*}$ to the best parameter set obtained for the optimisation with RSC = 3 (Fig 5B), in which the rates are not tightly constrained, and the two forward regulatory factors, $\gamma_{1,2}$ and $\gamma_{2,3}$, may be distinct from 1 (S10A Fig). We observed significant rate scale separation in this parameter set and a value of $\gamma_{1,2}\approx 1$ (Fig. 5H). To make this analysis comparable to that in Fig. 6A–C, we fixed $\gamma_{1,2}=1$ and did not subject it to perturbations. We found that perturbing the other parameters as before yielded very similar $\Delta \Delta_{\overset{―}{SS}}$ distributions to the analysis in Fig 6C (S10C Fig). As for $\Delta \Delta_{{\overset{―}{mFPT}}^{\langle U\rangle }}$, we observed qualitatively similar results to the analysis in Fig 6B, but with slightly greater sensitivity to perturbations in $\ell_{1,2}$ and $\ell_{2,3}$ (S10B Fig). We then set $\theta^{*}$ to the best parameter set obtained for the optimisation with RSC = 0.005 (Fig 5E, green line, and S10D Fig), in which the rates are tightly constrained, similar to the best parameter set in Fig 4D analysed in Fig. 6D–F. Again, we found that parameter perturbations yield a very similar pattern of $\Delta \Delta_{\overset{―}{SS}}$ distributions to the corresponding analysis in Fig 6F (S10F Fig), and a qualitatively similar pattern of $\Delta \Delta_{{\overset{―}{mFPT}}^{\langle U\rangle }}$ distributions to the analysis in Fig 6E (S10E Fig). Finally, we set $\theta^{*}$ to the same parameter set but with $\ell_{2,1}$, $\ell_{2,3}$, and $\ell_{3,2}$ set to the $\ell_{1,2}$ value, and treated all four parameters as one common parameter, *ℓ* (S10G Fig), akin to our previous analysis in Fig 6G–I. Again, the resulting $\Delta \Delta_{\overset{―}{SS}}$ and $\Delta \Delta_{{\overset{―}{mFPT}}^{\langle U\rangle }}$ distributions (S10I Fig and S10H Fig, respectively) were similar to the corresponding distributions in Fig 6I and H.

Finally, we asked how this picture changes when we constrain the parameters to better reflect plausible scenarios in biological input-output systems. Specifically, we used our optimisation procedure to identify parameter sets that achieve a prescribed minimum value for $\Delta_{\overset{―}{SS}}$ over a reduced input concentration range, as well as a target value for ${mFPT}^{U_{1}}$ along that range, while also achieving strong decoupling globally.

We chose a reduced input concentration range spanning three orders of magnitude,


$$\begin{array}{c} x\in \left[10^{-3}\;\text{c.u.},1\;\text{c.u.}\right], \end{array}$$ (27)

which is a plausible estimate for spatial morphogen gradients in developing embryos [42,43]; and sought to achieve a steady-state dynamic range that exceeds half its theoretical maximum, i.e.,


$$\begin{array}{c} \Delta_{\overset{―}{SS}}>0.5. \end{array}$$ (28)

We also chose a target ${mFPT}^{U_{1}}$ value of $\delta^{-1}$, so as to attain an activation time that roughly scales with the half-life of the readout, *M*; this is again broadly consistent with measurements in gene regulation systems, in which both the activation time and the half-life of the produced mRNA are often on the order of minutes [44,45]. Since ${mFPT}^{U_{1}}$ can change with *x*, we sought to enforce this target value by implementing the constraint,


$$\begin{array}{c} {mFPT}^{U_{1}}(x=10^{-3}\;\text{c.u.})\in \left[0.9\delta^{-1},1.1\delta^{-1}\right]. \end{array}$$ (29)

That is, we accepted values within 10% of the target value. We first ran the optimisation procedure with these three new constraints, to obtain a parameter set that achieves strong decoupling through a rate scale separation mechanism (S11A Fig). We then ran the optimisation procedure with these three constraints, plus the additional constraint that


$$\begin{array}{c} \ell_{1,2}=\ell_{2,1}=\ell_{2,3}=\ell_{3,2}=\ell , \end{array}$$

to attain a parameter set that achieves strong decoupling with significant rate scale constraint (S11B Fig).

We then performed the same sensitivity analysis as described above on these new parameter sets. Here, we computed $\Delta \Delta_{{\overset{―}{mFPT}}^{U_{1}}}$ and $\Delta \Delta_{\overset{―}{SS}}$ over the reduced concentration range, and we also computed the change in ${mFPT}^{U_{1}}$ at *x* = 10^−3^ c.u. due to each parameter perturbation, which we defined as


$$\begin{array}{cc} \Delta {mFPT}^{U_{1}}(x=10^{-3}\;\text{c.u.}) & ={mFPT}^{U_{1}}(x=10^{-3}\text{c.u.};\theta^{\left(i\right)})\\ & \;-{mFPT}^{U_{1}}(x=10^{-3}\;\text{c.u.};\theta^{*}). \end{array}$$ (30)

For the rate scale separation mechanism, we found that only perturbing the slow forward rate, $\ell_{1,2}$, incurs large changes in ${mFPT}^{U_{1}}(x=10^{-3}\;\text{c.u.})$ (S11C Fig, left), which is consistent with the intuition that the corresponding transitions, $U_{1}\to U_{2}$ and $B_{1}\to B_{2}$, are rate-limiting. When the horizontal rates are constrained to be equal, we found that perturbing the one common rate, *ℓ*, incurs similarly large changes in ${mFPT}^{U_{1}}$ (S11D Fig, left).

Meanwhile, we observed largely similar patterns of changes in $\Delta_{{\overset{―}{mFPT}}^{U_{1}}}$ to the corresponding parameter sets in Fig 6A–C and 6G–I, except for a few parameters. First, we found that, for the rate scale separation mechanism, $\Delta_{{\overset{―}{mFPT}}^{U_{1}}}$ is more sensitive to perturbations in $\ell_{1,2}$ and $\ell_{2,3}$ than in the previous analysis (compare Fig 6B with S11C Fig, center). This may be due to the enforcement of a target value for ${mFPT}^{U_{1}}$ (Eq. 29), which yielded an ${mFPT}^{U_{1}}$ value of $1.03\;\delta^{-1}$ for the parameter set in S11A Fig, as opposed to a value of $\sim 1000\;\delta^{-1}$ in the corresponding parameter set obtained without this constraint, in Fig 6A. Second, we found that, for the incoherent mechanism, $\Delta_{{\overset{―}{mFPT}}^{U_{1}}}$ is much less sensitive to perturbations in the regulatory factor, $\gamma_{1,2}$, than in the previous analysis (compare Fig 6H with S11D Fig, center). In S11E Fig, we show that perturbing $\gamma_{1,2}$ actually gives rise to variation in ${\overset{―}{mFPT}}^{U_{1}}$ *outside* the concentration range defined in Eq. 27 (compare with the unperturbed ${\overset{―}{mFPT}}^{U_{1}}$ curve in S11B Fig). We also note that, in S11B Fig, the range of *x* over which $\overset{―}{SS}(x)$ changes does not overlap with the corresponding range for ${\overset{―}{mFPT}}^{U_{1}}$, akin to the optimal parameter sets in Fig 4B (see Fig 4G). These results suggest that this more local form of decoupling is less sensitive to perturbations in $\gamma_{1,2}$. Finally, we observed mostly similar patterns of changes in $\Delta_{\overset{―}{SS}}$ as in the previous analysis (compare Fig 6C and I with S11C Fig, right, and S11D Fig, right, respectively).

In all, these findings suggest that imposing the additional constraints we have introduced in Eqs. 27–29 can alter the sensitivity patterns of decoupling to single-parameter variations, but in a manner such that decoupling is only sensitive to perturbations outside the concentration range of interest. We also find that the strength of decoupling over the reduced concentration range is still less sensitive than other features, such as the activation time itself over the same range.

## Discussion

In this work, we sought to identify regulatory strategies that enable an input ligand to modulate the steady-state level of a molecular readout while maintaining a constant activation time, defined as the time for the first readout molecule to be produced. By systematically analysing Markov process models of an input-output system in which a ligand binds to a single regulatory site, we found two distinct regimes that support such decoupling between steady-state level and activation time. In the first regime, which we call *rate scale separation*, different transitions in the system proceed on different timescales, the system first undergoes slow transitions and then fast transitions, and the ligand does not regulate the slow forward transitions. In this way, the ligand controls the steady-state level whereas the slow, rate-limiting forward transitions dictate the activation time in a ligand-independent manner. In the second regime, the ligand acts as an *incoherent regulator*, exerting mutually opposing effects on different transitions towards the productive state.

The rate scale separation scenario aligns well with the setting of transcriptional regulation, where it is well-known that different regulatory steps—such as chromatin remodelling, transcription initiation, and RNA polymerase pausing—operate on distinct timescales (reviewed in [41]), and TFs may selectively regulate a subset of these processes [27,46,47]. In this way, a TF may regulate the steady-state level without affecting the activation time.

This idea is in line with the data reported in Eck et al. [9] on the regulation of *hunchback* by Bicoid and Zelda in the *Drosophila* blastoderm. As we mentioned in the Introduction, in Eck et al., the authors report that in WT embryos, a *hunchback* reporter exhibits a constant activation time over the antero-posterior axis of the embryo, despite the presence of a Bicoid concentration gradient along this axis, which only affects the reporter transcription levels. However, in embryos lacking the pioneer factor Zelda, which is regarded to be present at a roughly constant level along the antero-posterior axis, both the activation time and the transcription level were found to depend on Bicoid concentration. One plausible interpretation of this data, according to our findings here, is that the activation time is determined by the pioneering activity of Zelda, which would continuously promote the slow process of chromatin opening throughout the embryo. In turn, Bicoid would primarily control the faster process of RNA polymerase loading. In Appendix G in S1 Text and S12 Fig we show an adaptation of the ${𝒟}_{3}$ model to qualitatively account for these data.

In a more recent paper, Alamos et al. [48] reported similar observations of decoupling with a synthetic enhancer that binds the morphogen Dorsal in the *Drosophila* embryo. However, Alamos et al. proposed a different model, namely a model based on the Erlang process [49], to explain these data. This model is equivalent to a version of the chain model, ${𝒞}_{N}$, in which all backward transitions are omitted and all forward transitions have the same rate, which depends on Dorsal concentration. While Alamos et al. showed that this model can recapitulate the observed decoupling on average, their analysis also assumed a short time window (~ 7 min) during which transcription could be activated. Such a time window is naturally imposed by the fast nuclear division times in the early *Drosophila* embryo [50], but this assumption also effectively truncates the distribution of possible activation times in a fashion that causes the mean of this distribution to appear independent of Dorsal concentration (S13A,D Fig). In other words, this distribution may be effectively interpreted as that of the *conditional* FPT to transcriptional activation, given that activation indeed occurs within the 7 min window [21]. Alamos et al. observed that the fraction of nuclei that undergo activation within this observation window *does* increase with Dorsal concentration. It is therefore plausible that extending the observation window could potentially shift this conditional FPT distribution, and its mean, in a manner that depends on Dorsal concentration (S13 Fig). Indeed, we have found that allowing for longer observation windows in Alamos et al.’s model yields an activation time that does depend on Dorsal concentration (Appendix H in S1 Text, S13D Fig). These findings raise the question of whether the decoupling reported by Alamos et al. is an intrinsic feature of that enhancer system, or rather emerges as a byproduct of the fast nuclear cycles within the *Drosophila* embryo [50]. Further experiments that accommodate live imaging of this system over longer observation windows, perhaps outside the context of the *Drosophila* embryo, are required to resolve this question.

In addition to rate scale separation, we have also found that decoupled responses can arise under incoherent regulation. Such regulatory regime is increasingly recognised as a feature of gene regulation [24,51], but a thorough mechanistic understanding of how incoherence arises, and what consequences this regulatory mode harbors for biological function, is still lacking. Our analyses reveal that one such consequence may be decoupling, albeit to a less robust extent than the decoupling obtained under rate scale separation. Our optimisation results in Fig 4 and S6–S8 Fig also suggest that the ability of an incoherent regulator to achieve decoupling may depend on which transitions are regulated. We envision that further work should aim to identify the origins of these dependencies, as well as the differing constraints and tradeoffs that apply to systems under rate scale separation and incoherent regulation.

Among the incoherent parameter sets that exhibit decoupling with respect to the ${\overset{―}{mFPT}}^{U_{1}}$ definition of activation time, we noted that those pertaining to some regulatory regimes have low unbinding rates, *k*_off_ (Fig 4D and S7 Fig). However, in the context of gene regulation, experimental measurements of TF–DNA dissociation rates generally tend to be fast [40,41]. This is more consistent with the results in S8 Fig, which show that when $B_{2}\to B_{1}$ and $B_{3}\to B_{2}$ are regulated, larger values of *k*_off_ can support decoupling. In addition, our analysis of decoupling with respect to ${\overset{―}{mFPT}}^{\langle U\rangle }$ yielded a collection of parameter sets with greater variability in *k*_off_, and we were indeed able to identify parameter sets with larger *k*_off_ values that support decoupling. We suspect that the need for a relatively low *k*_off_ in some regulatory regimes arises from our assumption that regulation occurs only while the ligand is bound. We anticipate that decoupling and fast unbinding can co-occur more consistently in more complex models, for example by including “memory states” that maintain the regulatory effect of the ligand after it has unbound, or by including states of intermediate binding [52].

Our sensitivity analysis shows that decoupling in our models is much more sensitive to some parameter variations than to others. However, our analysis was restricted to single-parameter variations. Simultaneous perturbations in multiple parameters could well reveal additional features. For example, in our analysis of the incoherent regulation mechanism (Fig 6D–F), we found that decoupling is quite sensitive to changes in the horizontal rates, which must be similar to facilitate decoupling via incoherent regulation. However, it is possible—as already suggested by our analysis in Fig 6G–I—that coupled perturbations in all four horizontal rates could preserve the rate scale constraint that is required to facilitate decoupling within this regime. One well-established method for quantifying the effects of multi-parameter perturbations is to calculate the Hessian matrix, with respect to the model parameters, of an appropriately defined objective that quantifies the change in decoupling as a function of the parameters [53–55]. The eigenvalues and eigenvectors of this Hessian, evaluated at our optimal parameter sets (denoted $\theta^{*}$ in Fig 6), can be then used to identify parameter combinations for which the model is sensitive (“stiff”) or robust (“sloppy”) to variations. Such an analysis is likely to yield a more comprehensive understanding of the parametric robustness of decoupling but it lies outside the scope of the present paper.

Our analysis focused on particular choices of models—the chain models, ${𝒞}_{N}$, and the ladder models, ${𝒟}_{N}$—alongside additional assumptions, such as the assumption that the ligand regulates at most two horizontal transitions in ${𝒟}_{N}$. As such, there are several aspects of this analysis that may be generalised in future work. First, examining the effects of different numbers of internal states (*N*), beyond the specific cases of *N* = 2, *N* = 3 and *N* = 6 considered here. Second, our models assume a single ligand-binding site, as is the case in many experimental systems [27,48,56,57]; despite this, we also expect that incorporating multiple binding sites may yet reveal additional mechanisms for decoupling. In the same direction, incorporating multiple ligands would be insightful. Third, in the ladder models, we assumed that ligand unbinding proceeds with a constant rate, *k*_off_, that is independent of the system state. This assumption encodes the simple scenario in which progression towards the productive state does not influence the ligand’s unbinding kinetics. For example, a TF may bind to a regulatory element far from the promoter, but still affect the rate of polymerase assembly at the promoter in a way that does not modulate the TF binding properties. Other scenarios may be best accounted for by considering that ligand unbinding depends on the system state.

Gene regulation is a process that relies on various mechanisms of energy expenditure, especially in eukaryotes, where the genome is packaged into chromatin that must be continually remodelled and modified to facilitate gene regulation [5,9,23,24,58]. As such, in our models, we do not assume that the system relaxes towards a steady state of thermodynamic equilibrium. Yet, the equilibrium assumption has been commonly used in models of gene regulation, and investigating what new regulatory capabilities arise with non-equilibrium mechanisms is an active research area [58,59]. This raises the question of whether and how energy expenditure facilitates decoupling. As an initial examination of whether imposing thermodynamic equilibrium is detrimental for decoupling, we considered in Appendix I in S1 Text and S14 Fig an equilibrium “re-parametrisation” of the ${𝒟}_{3}$ model, in which the *k*_off_ rates depend on the regulatory factors, $\gamma_{1,2}$ and $\gamma_{2,3}$. In this model, the transition rates are defined such that the “regulatory” sub-system described by ${𝒟}_{3}$ relaxes to a state of thermodynamic equilibrium. Employing a simple analysis outlined in Appendix I in S1 Text, we observed no appreciable differences in the responses from those in the original non-equilibrium parametrisation. We leave open the intriguing question of whether this conclusion holds more generally to future work.

Our analyses relied on particular quantitative definitions of readout levels and activation time. We believe that the biological interpretation of the activation time as an mFPT in the underlying Markov process is straightforward. It is also experimentally tractable, as modern techniques now offer access to such timing information, often in the form of FPT distributions, in various molecular systems (e.g., [6,8,15,60,61]). On the other hand, other definitions of activation time may be considered. For example, recent studies in the literature [22,62–65] have considered the time required for the molecular readout to reach a given mean abundance. Notably, this quantity depends on the degradation rate of the molecule, in contrast to our definition (Appendix J in S1 Text, S15 Fig). It will be insightful to examine how decoupling arises for various definitions of activation time.

The relevance of the steady-state level as a measure of readout abundance is less clear, as there are many molecular systems that do not reach a steady state, especially in *in vivo* contexts in which the system is embedded in a highly dynamic environment. For instance, the transcriptional output of a gene rarely reaches a steady state, and RNA is typically produced in transient, stochastic bursts [66]. As such, while steady-state assumptions, or even assumptions of thermodynamic equilibrium, are widespread in theoretical studies of such systems [9,28,67–71], the overall validity of these assumptions are questionable. In our analysis, we focused on parameter regimes in which a flat, or nearly flat, activation time is accompanied by large changes in the steady-state response. This setup ensures that the flat activation time exhibited by our parameter sets is not a trivial consequence of, e.g., non-responsiveness to the ligand, but rather arises from nontrivial regulatory strategies. An interesting question for future work is the extent to which the mechanisms we have described here can achieve decoupling between activation time and a *transient* readout level.

Regarding functional consequences, decoupling between readout abundance and activation time has significant implications for input-output systems in biology. For example, cells across different spatial positions or lineages in a developmental system may need to respond synchronously to different local morphogen concentrations, which could be facilitated by a decoupling mechanism. Conversely, the ability to tune timing while maintaining fixed output levels could support functions that require fine-tuned activation timing regulation. These “reverse decoupling” regimes remain unexplored and merit further investigation. Additionally, applying similar analyses to alternative definitions of activation time—such as time to a threshold readout abundance [65], or to the onset of a burst in readout production—could deepen our understanding of dynamic regulatory mechanisms and improve the mechanistic interpretation of experimental data.

## Materials and methods

### Calculating the steady-state level, activation time, and their dynamic ranges

As described in the section “Modeling approach and mathematical setup,” we used both numerical and analytical approaches for calculating the steady-state level, activation time, and their dynamic ranges, which we defined using the graph-theoretic linear framework [17–20] (Appendix A in S1 Text). For the analytical calculations, we implemented a symbolic version of the Chebotarev–Agaev recurrence (Eq. 14), using the Python package SymPy [72]. Barring additional symbolic simplifications, this approach allows us to obtain exact algebraic expressions for the steady-state level and activation time for any graph, *G*, in *O*(*n*^4^) arithmetic operations, where *n* is the number of vertices in *G*. This is much more efficient than any method based on direct enumeration of the spanning trees and/or forests that feature in Eqs. 9 and 10, which can scale exponentially with *n* [19].

For the numerical calculations, we calculated the steady-state level by obtaining the SVD of ℒ(G), identifying the right singular vector corresponding to the zero singular value, and normalizing appropriately. This right singular vector is unique whenever *G* is strongly connected. The normalised vector, which is the steady-state vector, p^*^, arising from the master equation (Eq. 1), was then used to evaluate $\overset{―}{SS}(x)$, as per Eqs. 4 and 15. On the other hand, we calculated the activation time by obtaining the QR decomposition of the left-hand matrix in Eq. 8, and using the corresponding solution vector to Eq. 8 to evaluate ${\overset{―}{mFPT}}^{i}(x)$, as per Eq. 15. Briefly, a QR decomposition of an invertible matrix is a decomposition, 𝐀=𝐐𝐑, such that **Q** is orthogonal, ${𝐐}^{-1}={𝐐}^{T}$, and **R** is upper-triangular; such decompositions are useful for solving matrix equations of the form 𝐀𝐱=𝐛. While the exact runtime complexities of these decompositions can differ between implementations, we expect that they will be asymptotically faster or comparable to the *O*(*n*^4^) runtime of the Chebotarev–Agaev recurrence, and—more importantly—faster in practice due to the availability of highly optimised implementations.

All calculations were implemented in C++, using multiple-precision floating-point numbers from the Boost.Multiprecision library [73] with a precision of 100 digits, and implementations of SVD and QR, from the Eigen library [74]. Python bindings were implemented to call these C++ functions from Python, so that they could interface with the PyMoo optimisation suite (see below).

We checked that the mFPTs obtained from Eq. 13 agree well with estimates obtained from Gillespie simulations [32,75] on a simple four-vertex graph (S1A Fig). We sampled 100 combinations of values for the rates, each from a log-uniform distribution on the range $\left[10^{-3},10^{3}\right]$, and ran increasing numbers of Gillespie simulations starting from vertex 1 to estimate the mFPT to vertex 4. As expected, we observed that the agreement between these estimates and their corresponding exact values, given by Eq. 13, increases with the number of trajectories (S1B Fig).

To numerically compute the dynamic range for a given parameter set, we calculated $\overset{―}{SS}(x)$ and $\overset{―}{mFPT}(x)$ over a logarithmic concentration range of $\left[10^{-20},10^{20}\right]$, with a logarithmic stepsize of ~0.04004. We chose this wide range to ensure that these calculations capture the complete dynamic range of these outputs. For each output, we then identified the maximum and minimum value over this concentration range, and computed the dynamic ranges according to Eq. 16.

### Minimisation protocols for the decoupling score *f*

In preliminary investigations for this study, we compared various metaheuristic optimisation algorithms to find parameter sets that exhibit decoupling. We found the Particle Swarm Optimisation (PSO) implementation provided by PyMoo [38], a multi-objective optimisation suite, to be the most efficient option, and therefore used it for all the numerical analyses discussed in this work.

The PSO algorithm was first introduced by Kennedy and Eberhart in 1995 [37]. Conceptually, the procedure is based on a swarm of particles, each with an associated position and velocity vector, which are iteratively updated to minimise an objective function, *f*. We denote the position and velocity of the *i*-th particle along the *d*-th dimension at time *t* as $X_{d}^{\left(i\right)}(t)$ and $V_{d}^{\left(i\right)}(t)$, respectively. (Here, *t* is treated as an integer variable.) In our case, each particle corresponds to a parameter set, wi*t*h the particle’s position given by the parameter values. Meanwhile, the velocity of the *i*-th particle along the *d*-th dimension, $V_{d}^{\left(i\right)}(t)$, is determined by:

1. that particle’s position at which *f* attained its lowest value throughout the particle’s trajectory up to time *t*, which we denote by $P_{d}^{\left(i\right)}(t)$; and
2. the position at which *f* attained its lowest value throughout the entire swarm’s history up to time *t*, which we denote by *G*_*d*_(*t*).

More specifically, $V_{d}^{\left(i\right)}$ is given by


$$\begin{array}{c} V_{d}^{\left(i\right)}(t)=\omega V_{d}^{\left(i\right)}(t)+c_{1}r_{1}\left(P_{d}^{\left(i\right)}(t)-X_{d}^{\left(i\right)}(t)\right)+c_{2}r_{2}\left(G_{d}(t)-X_{d}^{\left(i\right)}(t)\right), \end{array}$$

where ω is an inertia factor, $r_{1},r_{2}\in [0,1)$ are noise coefficients representing the level of “craziness” in the optimisation, and *c*_1_ and *c*_2_ balance the contributions from the *i*-th particle’s “personal” behaviour ($P_{d}^{\left(i\right)}$) and the swarm’s global behaviour (*G*_*d*_) [37]. PyMoo dynamically adjusts ω, *c*_1_, and *c*_2_ throughout the optimisation, with initial values of ω=0.9 and $c_{1}=c_{2}=2$, following the prescription outlined in [76]. Finally, the position of each particle is updated as


$$\begin{array}{c} X_{d}^{\left(i\right)}(t+1)=X_{d}^{\left(i\right)}(t)+V_{d}^{\left(i\right)}(t). \end{array}$$

We ran PSO for each of the problems defined in the paper, passing a total of 100 different random seeds to obtain a population of optimal solutions. To sample the initial set of particles, we used Latin hypercube sampling, which is the default choice in PyMoo. To enforce parametric bounds, the parameters that fall outside the defined bounded range are set to the closest bound value during the optimisation.

As the termination criterion for each PSO run, we used either convergence to a score of *f* < 0.1 for more than five consecutive generations, or a computation time exceeding 23 hours. The termination criterion that was used for each PSO run is specified in the corresponding figure caption.

Optimisations were performed on the O2 High Performance Compute Cluster at Harvard Medical School and the Scientific Computing Core Facility of the Department of Medicine and Life Sciences at Pompeu Fabra University.

## Supporting information

S1 Text Supporting text containing Appendix A-J. (PDF)

S1 Fig Agreement between mFPT from the Chebotarev–Agaev recurrence (Eq. 13) and estimates from Gillespie simulations. **(A)** Three-state model with one absorbing state. Each edge label represents an infinitesimal transition rate of the associated Markov process. **(B)** Agreement between the theoretically derived mFPT and estimates using a Python implementation of the Gillespie algorithm, GillesPy2 [75]. Parameter values were sampled from a log-uniform distribution on [10^−3^, 10^3^]. “Max time” refers to the maximum simulation time, “# traj” to the number of simulated trajectories. The red dashed line represents the line where theoretical and simulated values coincide.
(TIF)

S2 FigConvergence of the optimisation results in Fig 4 and Fig 5.**(A)** Evolution of the coupling score, *f*, with the number of optimisation generations for the parameter sets shown in Fig 4 for RSC = 0.005 (with activation time defined as ${\overset{―}{mFPT}}^{U_{1}}$). **(B)** Evolution of *f* with the number of optimisation generations for the parameter sets shown in Fig 5 for RSC = 0.005 (with activation time defined as ${\overset{―}{mFPT}}^{\langle U\rangle }$). In both panels, the red line represents the trajectory of the optimal parameter set with the least *f*. **(C)** Values of the constraint functions *g*_1_, *g*_2_ and *g*_3_ at the end of each optimisation in A (left) and B (right). The *i*-th constraint is given by *g*_*i*_ < 0.(TIF)

S3 FigDiscrepancy between the concentration ranges over which $\overset{―}{SS}(x)$ and ${\overset{―}{mFPT}}^{U_{1}}(x)$ change significantly, for the parameter sets in Fig 4D.**(A)** *Left:* Comparison of $k_{\text{off}}/k_{\text{on}}$ against the concentration, *x*_1/2_, at which $\overset{―}{SS}(x)$ is half-maximal. *Right:* Comparison of $\ell_{1,2}/k_{\text{on}}$ against the concentration, *x*_fast_, at which ${\overset{―}{mFPT}}^{U_{1}}(x)$ is minimised. The red lines represent the loci at which each pair of quantities are equal. **(B)** $\overset{―}{SS}(x)$ and ${\overset{―}{mFPT}}^{U_{1}}(x)$ as functions of *x* (solid lines and dashed lines, respectively), with each parameter set to the value in the best parameter set in Fig 4D save for *k*_off_, which is varied as shown. **(C)** Values of *x*_1/2_ and *x*_fast_ for the best parameter set in Fig 4D, but with different values of *k*_off_. The size of each dot represents the value of $\Delta_{\overset{―}{SS}}$ (green) and $\Delta_{{\overset{―}{mFPT}}^{U_{1}}}$ (purple).(TIF)

S4 FigOptimisation results with $\ell_{1,2}=\ell_{2,3}=r$ and $\ell_{2,1}=\ell_{3,2}$, and with $\gamma_{1,2}$ and $\gamma_{2,3}$ regulated.**(A)** Optimisation results with *f* < 1, each coloured according to the value of *f*. **(B)** Steady-state response and activation time for the best parameter set: $\ell_{1,2}=\ell_{2,3}=r=9.999\delta$; $\ell_{2,1}=\ell_{3,2}=23.93\delta$; $k_{\text{on}}=0.403\delta /(1\;c.u.)$; $k_{\text{off}}=0.0001\delta$. **(C)** Scanning of the regulatory space for the best parameter set. The red area represents the minimum of the coupling score *f*.(TIF)

S5 FigComparison between theoretical and simulated activation times for the best parameter sets in Figs 4D (A) and 5E (B).Simulated mFPTs in both plots were obtained using GillesPy2 [75]. In **A**, each simulation trajectory was initialisede at *U*_1_, and run on the augmented graph, ${𝒟}_{3}^{+}$, for a maximum simulation time approximately 100 times larger than the reciprocal of the slowest horizontal rate in Fig 4D. In **B**, each simulation trajectory was first initialised at *U*_1_, then “pre-equilibrated” on the *non-augmented* graph, ${𝒟}_{3}$, with *x* = 0 for a simulation time approximately 100 times larger than the reciprocal of the slowest horizontal rate in Fig 5E. Then, starting from the last state visited during this pre-equilibration, we ran the simulation trajectory on the augmented graph, ${𝒟}_{3}^{+}$, with *x* > 0 for the same maximum simulation time. 100 simulation trajectories were run for both **A** and **B**. Error bars represent 99% confidence intervals of the mean. The corresponding theoretical values for ${mFPT}^{U_{1}}$ and ${mFPT}^{\langle U\rangle }$ were obtained using Eq. 13.(TIF)

S6 FigOptimisation results with $\ell_{1,2}=\ell_{2,3}=r$ and $\ell_{2,1}=\ell_{3,2}$, and with $\gamma_{2,1}$ and $\gamma_{2,3}$ regulated.**(A)** The optimisation was performed with $\ell_{1,2}=\ell_{2,3}=\ell_{f}$ and $\ell_{2,1}=\ell_{3,2}=\ell_{b}$. **(B)** After 100 optimisation iterations, we found no parameter sets with a decoupling score, *f*, significantly smaller than 1. **(C)** Distributions of parameter values obtained through the optimisation, with the black line denoting the best parameter set. All parameter values are given in units of δ, except for *k*_on_, which is given in units of δ/(1c.u.). **(D)** Steady-state response and activation time for the best parameter set, showing no significant decoupling.(TIF)

S7 FigOptimisation results with $\ell_{1,2}=\ell_{2,3}=r$ and $\ell_{2,1}=\ell_{3,2}$, and with $\gamma_{1,2}$ and $\gamma_{3,2}$ regulated.**(A)** The optimisation was performed with $\ell_{1,2}=\ell_{2,3}=\ell_{f}$ and $\ell_{2,1}=\ell_{3,2}=\ell_{b}$. **(B)** Optimisation results in the $\left(\gamma_{1,2},\gamma_{3,2}\right)$ regulatory space, showing significant decoupling in the incoherent space (red crosses), with $\gamma_{1,2}<1$ (the ligand hinders the forward transition, $B_{1}\to B_{2}$) and $\gamma_{3,2}<1$ (the ligand hinders the backward transition, $B_{3}\to B_{2}$). **(C)** Distributions of parameter values obtained through the optimisation, with the black line denoting the best parameter set. All parameter values are given in units of δ, except for *k*_on_, which is given in units of δ/(1c.u.). **(D)** Steady-state response and activation time for the best parameter set, showing significant decoupling.(TIF)

S8 FigOptimisation results with $\ell_{1,2}=\ell_{2,3}=r$ and $\ell_{2,1}=\ell_{3,2}$, and with $\gamma_{2,1}$ and $\gamma_{3,2}$ regulated.**(A)** The optimisation was performed with $\ell_{1,2}=\ell_{2,3}=\ell_{f}$ and $\ell_{2,1}=\ell_{3,2}=\ell_{b}$. **(B)** Optimisation results in the $\left(\gamma_{2,1},\gamma_{3,2}\right)$ regulatory space, showing significant decoupling in the incoherent space (red crosses), with $\gamma_{2,1}>1$ (the ligand promotes the backward transition, $B_{2}\to B_{1}$) and $\gamma_{3,2}<1$ (the ligand hinders the backward transition, $B_{3}\to B_{2}$). **(C)** Distributions of parameter values obtained through the optimisation, with the black line denoting the best parameter set. All parameter values are given in units of δ, except for *k*_on_, which is given in units of δ/(1c.u.). **(D)** Steady-state response and activation time for the best parameter set, showing significant decoupling.(TIF)

S9 FigResponse decoupling for *N =* 6.**(A–C)** Analytical formulas for $\Delta_{\overset{―}{SS}}$ and $\Delta_{{\overset{―}{mFPT}}^{U_{1}}}$ for each special parametrisation on the left. Each pair of formulas demonstrates that decoupling can be achieved under rate scale separation ($\ell_{2}\gg \ell_{1}$) when the ligand regulates one or both of the fast transitions. **(D)** We extended the best parameter set obtained from the optimisation with RSC = 0.005 from Fig 4D to *N* = 6, by setting the forward transition rates (including *r*) to the average forward transition rate in the ${𝒟}_{3}$ model, $(\ell_{1,2}+\ell_{2,3})/2$, and similarly setting the backward transition rates to the average backward transition rate in the ${𝒟}_{3}$ model, $(\ell_{2,1}+\ell_{3,2})/2$. We preserved the remaining parameter values, *k*_on_ and *k*_off_. Then, assuming that at most two transitions are regulated by the ligand, we ran through each possible pair of regulatory factors and sought to identify the values of these regulatory factors that attained the strongest decoupling. The plot shows the values of $\Delta_{\overset{―}{SS}}$ and $\Delta_{{\overset{―}{mFPT}}^{U_{1}}}$ for the best identified solutions. Decoupling is strongest in the lower right. **(E)** Heatmap of the coupling score, *f*, with respect to $\gamma_{4,5}$ and $\gamma_{5,6}$, which was the pair of regulatory factors with the strongest decoupling (highest $\Delta_{\overset{―}{SS}}$ and lowest $\Delta_{{\overset{―}{mFPT}}^{U_{1}}}$) in **D**. Note that *f* is minimised in the incoherent space. **(F–G)** Same as in **D** and **E**, but with the activation time defined in terms of ${mFPT}^{\langle U\rangle }$. Here, the best parameter set from Fig 5E was extended in the same way as in **D**.(TIF)

S10 FigSensitivity of decoupling via the rate scale separation (A–C) and incoherent regulation (D–I) mechanisms to parameter perturbations, with respect to the ${mFPT}^{\langle U\rangle }$ definition of activation time.**(A)** The best parameter set, $\theta^{*}$, obtained from the optimisation in Fig 5 with RSC = 3 (see also Fig 5B). **(B–C)** Distributions of $\Delta \Delta_{{\overset{―}{mFPT}}^{\langle U\rangle }}$ (**B**) and $\Delta \Delta_{\overset{―}{SS}}$ (**C**) arising from perturbations in each parameter, starting from the choice of $\theta^{*}$ in **A**. The colormap represents the log-ratio of the perturbed parameter value, $\theta_{i}^{\left(i\right)}$, with respect to the optimal parameter value, $\theta_{i}^{*}$. **(D)** The best parameter set $\theta^{*}$, obtained from the optimisation in Fig 5 with RSC = 0.005 (see also Fig 5E, green line). **(E–F)** Distributions of $\Delta \Delta_{{\overset{―}{mFPT}}^{\langle U\rangle }}$ (**E**) and $\Delta \Delta_{\overset{―}{SS}}$ (**F**) arising from perturbations in each parameter, starting from the choice of $\theta^{*}$ in **D**. **(G)** A modified version of the parameter set in **D**, in which all horizontal rates have been set to the same value, $\ell_{1,2}=\ell_{2,1}=\ell_{2,3}=\ell_{3,2}=\ell$, as described in the text. **(H–I)** Distributions of $\Delta \Delta_{{\overset{―}{mFPT}}^{\langle U\rangle }}$ (**H**) and $\Delta \Delta_{\overset{―}{SS}}$ (**I**) arising from perturbations in each parameter, starting from the choice of $\theta^{*}$ in **G**. Here, the horizontal rates were perturbed as a single parameter, *ℓ*, as described in the text.(TIF)

S11 FigSensitivity of decoupling over a finite concentration range and with a prescribed target ${mFPT}^{U_{1}}$.**(A)** The steady-state response and activation time (left) corresponding to the best parameter set, $\theta^{*}$, obtained from optimisation with the additional constraints in Eqs. 27–29 (right). The concentration range over which the additional constraints were enforced, $\left[10^{-3}\;\text{c.u.},1\;\text{c.u.}\right]$, is shown in red. **(B)** The steady-state response and activation time (left) corresponding to the best parameter set, $\theta^{*}$, obtained from optimisation with the additional constraints in Eqs. 27–29, as well as the constraint that all four horizontal rates are equal, as described in the text (right). The concentration range over which the additional constraints were enforced, $\left[10^{-3}\;\text{c.u.},1\;\text{c.u.}\right]$, is shown in red. **(C)** Distributions of $\Delta {mFPT}^{U_{1}}$, $\Delta \Delta_{{\overset{―}{mFPT}}^{U_{1}}}$, and $\Delta \Delta_{\overset{―}{SS}}$ arising from perturbations in each parameter, starting from the choice of $\theta^{*}$ in **A**. The colormap represents the log-ratio of the perturbed parameter value, $\theta_{i}^{\left(i\right)}$, with respect to the optimal parameter value, $\theta_{i}^{*}$. **(D)** Distributions of $\Delta {mFPT}^{U_{1}}$, $\Delta \Delta_{{\overset{―}{mFPT}}^{U_{1}}}$, and $\Delta \Delta_{\overset{―}{SS}}$ arising from perturbations in each parameter, starting from the choice of $\theta^{*}$ in **B**. Here, the horizontal rates were perturbed as a single parameter, *ℓ*, as described in the text. **(E)** Distributions of parameter values upon perturbing the regulator factor, $\gamma_{1,2}$ (left), and the corresponding collections of steady-state responses (center) and activation times (right) that arise from these perturbations. These plots reveal that, while these perturbations do give rise to a loss of decoupling, this predominantly occurs outside the concentration range over which the additional constraints were enforced.(TIF)

S12 FigAdaptation of the ${𝒟}_{3}$ model to account for the data in [9].We assume transtion rates on two different timescales, $\ell_{1}$ and $\ell_{2}$, and we assume rate scale separation with $\ell_{2}\gg \ell_{1}$. We assume that the pioneer factor Zelda, which we do not model explicitly given its constant concentration along the antero-posterior axis of the embryo, acts by increasing the magnitude of the slow transition rate, $\ell_{1}$, via the regulatory factor, $\gamma_{zld}>1$. On the other hand, we model the morphogen Bicoid, whose concentration does vary along the antero-posterior axis, as a ligand that binds and modulates the forward transitions, $B_{1}\to B_{2}$ and $B_{2}\to B_{3}$, via the regulatory factor, $\gamma_{bcd}>1$.(TIF)

S13 FigAnalysis of the Erlang process proposed in [48].FPT distributions to the terminal state in the Erlang process with *N* + 1 states, for N=2,…,5. The common transition rate, *k*([Dl]), was defined as described in Appendix H in S1 Text, with $c=0.55\;{\text{min}}^{-1}$ and $K_{d}=250\;\text{a.u.}$ [48], and different curves correspond to different concentrations of Dl (denoted by TF in the figure). The vertical lines represent the length of the time window during which transcription is active (*t* = 7 min). **(B)** The same FPT distributions as in **A**, but each normalised by the corresponding cumulative probability at *t* = 7. **(C)** Empirical distributions of the *conditional* FPT to the terminal state, given that the terminal state is indeed reached within a window of 7 min, in the Erlang process with *N* + 1 states, for N=2,…,5. The common transition rate was defined in the same way as in **A**, and the distributions were obtained using GillesPy2 [75]. **(D)** Average onset time (black), estimated from the empirical distributions in **C** as described in Appendix H in S1 Text; and the mFPT to the terminal state (red), calculated using Eq. 13, both as functions of Dl concentration. Each curve was normalised by its maximum value.(TIF)

S14 FigDecoupling in an equilibrium “re-parametrisation” of ${𝒟}_{3}$.**(A)** An equilibrium re-parametrisation of ${𝒟}_{3}$, assuming a regulatory regime in which only the second forward rate is regulated, as in Fig 3A. In particular, the off-rate for $B_{3}\to U_{3}$ is set to $k_{\text{off}}/\gamma_{2,3}$, so that the cycle condition is satisfied (Appendix I in S1 Text). **(B)** Steady-state response and activation time for this re-parametrisation, with the parameter sets from Fig 3E. We find that this re-parametrisation does not result in any significant differences from the corresponding curves in Fig 3D. **(C)** An equilibrium re-parametrisation of ${𝒟}_{3}$, assuming a regulatory regime in which both forward rates are regulated, as in Fig 3F. Here, the off-rates for $B_{2}\to U_{2}$ and $B_{3}\to U_{3}$ are set to $k_{\text{off}}/\gamma_{1,2}$ and $k_{\text{off}}/(\gamma_{1,2}\gamma_{2,3})$, respectively, so that the cycle condition is satisfied (Appendix I in S1 Text). **(D)** Steady-state response and activation time for this re-parametrisation, with the parameter sets from Fig 4D. We find that this re-parametrisation does not result in any significant differences from the corresponding curves in Fig 4B.(TIF)

S15 FigAverage time to first produce one readout molecule (mFPT) vs. time to produce an average copy-number of one molecule.**(A)** Analytical solution for the mean, $M(t)=\langle n_{M}\rangle$, of the readout copy-number in the master equation in Eq. S3 for the telegraph model, ${𝒞}_{2}$, with $\ell_{1,2}=a$ and $\ell_{2,1}=b$, with different choices of the degradation rate, δ. All other rates were set to *a* = *b* = *r* = 10 t.u. The time required to reach *M* = 1 increases with δ, as expected. On the contrary, the mFPT (Eq. 13) does not depend on δ. **(B)** mFPT estimates obtained from Gillespie simulations, performed using GillesPy2 [75], on ${𝒞}_{2}$. Error bars correspond to 99% confidence intervals around the mean, each obtained from 500 simulation trajectories.(TIF)

## References

[1] Qiao L, Ghosh P, Rangamani P. Design principles of improving the dose-response alignment in coupled GTPase switches. NPJ Syst Biol Appl. 2023;9(1):3. doi: 10.1038/s41540-023-00266-9 36720885 PMC9889403

[2] Calabrese EJ, Baldwin LA. Hormesis: the dose-response revolution. Annu Rev Pharmacol Toxicol. 2003;43:175–97. doi: 10.1146/annurev.pharmtox.43.100901.140223 12195028

[3] Tallarida RJ, Jacob LS. The dose—response relation in pharmacology. 1979th ed. New York, NY: Springer; 2012.

[4] Yordanov P, Stelling J. Steady-state differential dose response in biological systems. Biophys J. 2018;114(3):723–36. doi: 10.1016/j.bpj.2017.11.3780 29414717 PMC5985043

[5] Estrada J, Wong F, DePace A, Gunawardena J. Information integration and energy expenditure in gene regulation. Cell. 2016;166(1):234–44. 27368104 10.1016/j.cell.2016.06.012 PMC4930556

[6] Rochais F, Vilardaga J-P, Nikolaev VO, Bünemann M, Lohse MJ, Engelhardt S. Real-time optical recording of beta1-adrenergic receptor activation reveals supersensitivity of the Arg389 variant to carvedilol. J Clin Invest. 2007;117(1):229–35. doi: 10.1172/JCI30012 17200720 PMC1751291

[7] Gibbons MM, Chou T, D’Orsogna MR. Diffusion-dependent mechanisms of receptor engagement and viral entry. J Phys Chem B. 2010;114(46):15403–12. doi: 10.1021/jp1080725 21038861

[8] Donovan BT, Huynh A, Ball DA, Patel HP, Poirier MG, Larson DR, et al. Live-cell imaging reveals the interplay between transcription factors, nucleosomes, and bursting. EMBO J. 2019;38(12):e100809. doi: 10.15252/embj.2018100809 31101674 PMC6576174

[9] Eck E, Liu J, Kazemzadeh-Atoufi M, Ghoreishi S, Blythe SA, Garcia HG. Quantitative dissection of transcription in development yields evidence for transcription-factor-driven chromatin accessibility. Elife. 2020;9:e56429. doi: 10.7554/eLife.56429 33074101 PMC7738189

[10] Fernandes G, Tran H, Andrieu M, Diaw Y, Perez Romero C, Fradin C, et al. Synthetic reconstruction of the hunchback promoter specifies the role of Bicoid, Zelda and Hunchback in the dynamics of its transcription. Elife. 2022;11:e74509. doi: 10.7554/eLife.74509 35363606 PMC8975551

[11] Keller SH, Jena SG, Yamazaki Y, Lim B. Regulation of spatiotemporal limits of developmental gene expression via enhancer grammar. Proc Natl Acad Sci U S A. 2020;117(26):15096–103. doi: 10.1073/pnas.1917040117 32541043 PMC7334449

[12] Biswas K, Ghosh A. First passage time in post-transcriptional regulation by multiple small RNAs. Eur Phys J E Soft Matter. 2021;44(2):16. doi: 10.1140/epje/s10189-021-00028-7 33683458

[13] Syed S, Duan Y, Lim B. Modulation of protein-DNA binding reveals mechanisms of spatiotemporal gene control in early Drosophila embryos. Elife. 2023;12:e85997. doi: 10.7554/eLife.85997 37934571 PMC10629816

[14] Alamos S, Reimer A, Niyogi KK, Garcia HG. Quantitative imaging of RNA polymerase II activity in plants reveals the single-cell basis of tissue-wide transcriptional dynamics. Nat Plants. 2021;7(8):1037–49. doi: 10.1038/s41477-021-00976-0 34373604 PMC8616715

[15] Harden TT, Vincent BJ, DePace AH. Transcriptional activators in the early Drosophila embryo perform different kinetic roles. Cell Syst. 2023;14(4):258–272.e4. doi: 10.1016/j.cels.2023.03.006 37080162 PMC10473017

[16] Lucas T, Ferraro T, Roelens B, De Las Heras Chanes J, Walczak AM, Coppey M, et al. Live imaging of bicoid-dependent transcription in Drosophila embryos. Curr Biol. 2013;23(21):2135–9. doi: 10.1016/j.cub.2013.08.053 24139736

[17] Gunawardena J. A linear framework for time-scale separation in nonlinear biochemical systems. PLoS One. 2012;7(5):e36321. doi: 10.1371/journal.pone.0036321 22606254 PMC3351455

[18] Mirzaev I, Gunawardena J. Laplacian dynamics on general graphs. Bull Math Biol. 2013;75(11):2118–49. doi: 10.1007/s11538-013-9884-8 24018536

[19] Nam K-M, Martinez-Corral R, Gunawardena J. The linear framework: using graph theory to reveal the algebra and thermodynamics of biomolecular systems. Interface Focus. 2022;12(4):20220013. doi: 10.1098/rsfs.2022.0013 35860006 PMC9184966

[20] Nam K-M, Gunawardena J. The linear framework II: using graph theory to analyse the transient regime of Markov processes. Front Cell Dev Biol. 2023;11:1233808. doi: 10.3389/fcell.2023.1233808 38020901 PMC10656611

[21] Nam K-M, Gunawardena J. Algebraic formulas for first-passage times of Markov processes in the linear framework. Bull Math Biol. 2025;87(11):161. doi: 10.1007/s11538-025-01524-z 41085571 PMC12521310

[22] Ali MZ, Guharajan S, Parisutham V, Brewster RC. Regulatory properties of transcription factors with diverse mechanistic function. PLoS Comput Biol. 2024;20(6):e1012194. doi: 10.1371/journal.pcbi.1012194 38857275 PMC11192337

[23] Mahdavi SD, Salmon GL, Daghlian P, Garcia HG, Phillips R. Flexibility and sensitivity in gene regulation out of equilibrium. Proc Natl Acad Sci U S A. 2024;121(46):e2411395121. doi: 10.1073/pnas.2411395121 39499638 PMC11573582

[24] Martinez-Corral R, Friedrich D, Frömel R, Velten L, Gunawardena J, DePace AH. Emergence of activation or repression in transcriptional control under a fixed molecular context. Proc Natl Acad Sci U S A. 2025;122(39):e2413715122. doi: 10.1073/pnas.2413715122 40982681 PMC12501156

[25] Biddle JW, Martinez-Corral R, Wong F, Gunawardena J. Allosteric conformational ensembles have unlimited capacity for integrating information. Elife. 2021;10:e65498. doi: 10.7554/eLife.65498 34106049 PMC8189718

[26] Van Kampen NG. Stochastic Processes in Physics and Chemistry. 3rd ed. Amsterdam: Springer; 2007.

[27] Martinez-Corral R, Park M, Biette KM, Friedrich D, Scholes C, Khalil AS, et al. Transcriptional kinetic synergy: a complex landscape revealed by integrating modeling and synthetic biology. Cell Syst. 2023;14(4):324–339.e7. doi: 10.1016/j.cels.2023.02.003 37080164 PMC10472254

[28] Sánchez A, Kondev J. Transcriptional control of noise in gene expression. Proc Natl Acad Sci U S A. 2008;105(13):5081–6. doi: 10.1073/pnas.0707904105 18353986 PMC2278180

[29] Nasser J, Nam KM, Gunawardena J. A mathematical model clarifies the ABC score formula used in enhancer-gene prediction. eLife. 2025.

[30] Beilina L, Karchevskii E, Karchevskii M. Numerical linear algebra: theory and applications. 1st ed. Cham, Switzerland: Springer International Publishing; 2017.

[31] Chebotarev P, Agaev R. Forest matrices around the Laplacian matrix. Linear Algebra Appl. 2002;356(1–3):253–74. doi: 10.1016/s0024-3795(02)00388-9

[32] Gillespie DT. Exact stochastic simulation of coupled chemical reactions. J Phys Chem. 1977;81(25):2340–61.

[33] Meeussen JVW, Lenstra TL. Time will tell: comparing timescales to gain insight into transcriptional bursting. Trends Genet. 2024;40(2):160–74. doi: 10.1016/j.tig.2023.11.003 38216391 PMC10860890

[34] Peccoud J, Ycart B. Markovian modeling of gene-product synthesis. Theor Popul Biol. 1995;48(2):222–34.

[35] Dufourt J, Trullo A, Hunter J, Fernandez C, Lazaro J, Dejean M, et al. Temporal control of gene expression by the pioneer factor Zelda through transient interactions in hubs. Nat Commun. 2018;9(1):5194. doi: 10.1038/s41467-018-07613-z 30518940 PMC6281682

[36] Szczurek AT, Dimitrova E, Kelley JR, Klose RJ. Polycomb sustains promoters in a deep OFF-state by limiting PIC formation to counteract transcription. bioRxiv. 2023;2023:2023.06.13.544762. 10.1038/s41556-024-01493-w PMC11469961 39261718

[37] Kennedy J, Eberhart R. Particle swarm optimization. In: Proceedings of ICNN’95 - International Conference on Neural Networks. vol. 4. IEEE; 2002. pp. 1942–8.

[38] Blank J, Deb K. Pymoo: multi-objective optimization in Python. IEEE Access. 2020;8:89497–509. doi: 10.1109/access.2020.2990567

[39] Lammers NC, Kim YJ, Zhao J, Garcia HG. A matter of time: Using dynamics and theory to uncover mechanisms of transcriptional bursting. Curr Opin Cell Biol. 2020;67:147–57. doi: 10.1016/j.ceb.2020.08.001 33242838 PMC8498946

[40] Meeussen JVW, Lenstra TL. Time will tell: comparing timescales to gain insight into transcriptional bursting. Trends Genet. 2024;40(2):160–74. doi: 10.1016/j.tig.2023.11.003 38216391 PMC10860890

[41] Lammers NC, Kim YJ, Zhao J, Garcia HG. A matter of time: using dynamics and theory to uncover mechanisms of transcriptional bursting. Curr Opin Cell Biol. 2020;67:147–57. doi: 10.1016/j.ceb.2020.08.001 33242838 PMC8498946

[42] Lewis J, Slack JM, Wolpert L. Thresholds in development. J Theor Biol. 1977;65(3):579–90. doi: 10.1016/0022-5193(77)90216-8 859349

[43] Gregor T, Wieschaus EF, McGregor AP, Bialek W, Tank DW. Stability and nuclear dynamics of the bicoid morphogen gradient. Cell. 2007;130(1):141–52. doi: 10.1016/j.cell.2007.05.026 17632061 PMC2253672

[44] Forbes Beadle L, Love JC, Shapovalova Y, Artemev A, Rattray M, Ashe HL. Combined modelling of mRNA decay dynamics and single-molecule imaging in the Drosophila embryo uncovers a role for P-bodies in 5’ to 3’ degradation. PLoS Biol. 2023;21(1):e3001956. doi: 10.1371/journal.pbio.3001956 36649329 PMC9882958

[45] Little SC, Tikhonov M, Gregor T. Precise developmental gene expression arises from globally stochastic transcriptional activity. Cell. 2013;154(4):789–800. doi: 10.1016/j.cell.2013.07.025 23953111 PMC3778922

[46] Blau J, Xiao H, McCracken S, O’Hare P, Greenblatt J, Bentley D. Three functional classes of transcriptional activation domain. Mol Cell Biol. 1996;16(5):2044–55. doi: 10.1128/MCB.16.5.2044 8628270 PMC231191

[47] Rahl PB, Lin CY, Seila AC, Flynn RA, McCuine S, Burge CB, et al. c-Myc regulates transcriptional pause release. Cell. 2010;141(3):432–45. doi: 10.1016/j.cell.2010.03.030 20434984 PMC2864022

[48] Alamos S, Reimer A, Westrum C, Turner MA, Talledo P, Zhao J, et al. Minimal synthetic enhancers reveal control of the probability of transcriptional engagement and its timing by a morphogen gradient. Cell Syst. 2023;14(3):220–236.e3. doi: 10.1016/j.cels.2022.12.008 36696901 PMC10125799

[49] David A, Larry S. The least variable phase type distribution is erlang. Commun Stat. 1987;3(3):467–73.

[50] Desponds J, Tran H, Ferraro T, Lucas T, Perez Romero C, Guillou A, et al. Precision of readout at the hunchback gene: analyzing short transcription time traces in living fly embryos. PLoS Comput Biol. 2016;12(12):e1005256. doi: 10.1371/journal.pcbi.1005256 27942043 PMC5152799

[51] Guharajan S, Chhabra S, Parisutham V, Brewster RC. Quantifying the regulatory role of individual transcription factors in Escherichia coli. Cell Rep. 2021;37(6):109952. doi: 10.1016/j.celrep.2021.109952 34758318 PMC8667592

[52] Schaepe JM, Fries T, Doughty BR, Ramalingam V, Liu BB, Crocker OJ, et al. Thermodynamic principles link in vitro transcription factor affinities to single-molecule chromatin states in cells. Cell. 2026;189(1):307–322.e23. doi: 10.1016/j.cell.2025.11.008 41308636 PMC13397507

[53] Gutenkunst RN, Waterfall JJ, Casey FP, Brown KS, Myers CR, Sethna JP. Universally sloppy parameter sensitivities in systems biology models. PLoS Comput Biol. 2007;3(10):1871–8. doi: 10.1371/journal.pcbi.0030189 17922568 PMC2000971

[54] Bauer M, Bialek W, Goddard C, Holmes CM, Krishnamurthy K, Palmer SE, et al. Optimization and variability can coexist. arXiv. 2025.

[55] Zoller B, Bénichou A, Gregor T, Tkačik G. Invariant non-equilibrium dynamics of transcriptional regulation optimize information flow. arXiv. 2025.

[56] Mafi A, Kim S-K, Goddard WA 3rd. The mechanism for ligand activation of the GPCR-G protein complex. Proc Natl Acad Sci U S A. 2022;119(18):e2110085119. doi: 10.1073/pnas.2110085119 35452328 PMC9170043

[57] Hammar P, Walldén M, Fange D, Persson F, Baltekin O, Ullman G, et al. Direct measurement of transcription factor dissociation excludes a simple operator occupancy model for gene regulation. Nat Genet. 2014;46(4):405–8. doi: 10.1038/ng.2905 24562187 PMC6193529

[58] Wong F, Gunawardena J. Gene regulation in and out of equilibrium. Annu Rev Biophys. 2020;49:199–226. doi: 10.1146/annurev-biophys-121219-081542 32375018

[59] Zoller B, Gregor T, Tkačik G. Eukaryotic gene regulation at equilibrium, or non? Curr Opin Syst Biol. 2022;31:100435. doi: 10.1016/j.coisb.2022.100435 36590072 PMC9802646

[60] Berrocal A, Lammers NC, Garcia HG, Eisen MB. Kinetic sculpting of the seven stripes of the Drosophila even-skipped gene. Elife. 2020;9:e61635. doi: 10.7554/eLife.61635 33300492 PMC7864633

[61] Garcia HG, Tikhonov M, Lin A, Gregor T. Quantitative imaging of transcription in living Drosophila embryos links polymerase activity to patterning. Curr Biol. 2013;23(21):2140–5. doi: 10.1016/j.cub.2013.08.054 24139738 PMC3828032

[62] Ghusinga KR, Dennehy JJ, Singh A. First-passage time approach to controlling noise in the timing of intracellular events. Proc Natl Acad Sci U S A. 2017;114(4):693–8. doi: 10.1073/pnas.1609012114 28069947 PMC5278449

[63] Co AD, Lagomarsino MC, Caselle M, Osella M. Stochastic timing in gene expression for simple regulatory strategies. Nucleic Acids Res. 2017;45(3):1069–78. doi: 10.1093/nar/gkw1235 28180313 PMC5388427

[64] Gupta S, Varennes J, Korswagen HC, Mugler A. Temporal precision of regulated gene expression. PLoS Comput Biol. 2018;14(6):e1006201. doi: 10.1371/journal.pcbi.1006201 29879102 PMC5991653

[65] Ham L, Coomer MA, Öcal K, Grima R, Stumpf MPH. A stochastic vs deterministic perspective on the timing of cellular events. Nat Commun. 2024;15(1):5286. doi: 10.1038/s41467-024-49624-z 38902228 PMC11190182

[66] Rodriguez J, Ren G, Day CR, Zhao K, Chow CC, Larson DR. Intrinsic dynamics of a human gene reveal the basis of expression heterogeneity. Cell. 2019;176(1–2):213–226.e18. doi: 10.1016/j.cell.2018.11.026 30554876 PMC6331006

[67] Bintu L, Buchler NE, Garcia HG, Gerland U, Hwa T, Kondev J, et al. Transcriptional regulation by the numbers: models. Curr Opin Genet Dev. 2005;15(2):116–24. doi: 10.1016/j.gde.2005.02.007 15797194 PMC3482385

[68] Bintu L, Buchler NE, Garcia HG, Gerland U, Hwa T, Kondev J, et al. Transcriptional regulation by the numbers: applications. Curr Opin Genet Dev. 2005;15(2):125–35. doi: 10.1016/j.gde.2005.02.006 15797195 PMC3462814

[69] Garcia HG, Kondev J, Orme N, Theriot JA, Phillips R. Thermodynamics of biological processes. Methods Enzymol. 2011;492:27–59. doi: 10.1016/B978-0-12-381268-1.00014-8 21333788 PMC3264492

[70] Phillips R, Kondev J, Theriot J, Garcia H. Physical biology of the cell. 2nd ed. London, England: Garland Science; 2012.

[71] Landman J, Georgiev RN, Rydenfelt M, Kegel WK. In vivo and in vitro consistency of thermodynamic models for transcription regulation. Phys Rev Res. 2019;1(3):033094.

[72] Meurer A, Smith CP, Paprocki M, Čertík O, Kirpichev SB, Rocklin M, et al. SymPy: symbolic computing in Python. PeerJ Comput Sci. 2017;3:e103. doi: 10.7717/peerj-cs.103

[73] Boost C++ Libraries. [cited 2024 Oct 16]. Avialble from: https://www.boost.org/

[74] Guennebaud G, Jacob B, et al. Eigen v3. 2010. Avilable from: http://eigen.tuxfamily.org

[75] Matthew S, Carter F, Cooper J, Dippel M, Green E, Hodges S, et al. GillesPy2: a biochemical modeling framework for simulation driven biological discovery. Lett Biomath. 2023;10(1):87–103. 37655179 PMC10470263

[76] Zhan Z-H, Zhang J, Li Y, Chung HS-H. Adaptive particle swarm optimization. IEEE Trans Syst Man Cybern B Cybern. 2009;39(6):1362–81. doi: 10.1109/TSMCB.2009.2015956 19362911

