# Supplementary material for: Decoupling between activation time and steady-state level in input-output responses
Source: PLoS Comput Biol. 2026 May 15;22(5):e1014288. doi: 10.1371/journal.pcbi.1014288 (PMC13245878; doi:10.1371/journal.pcbi.1014288)
Supplement: S1 Text — (PDF) [file pcbi.1014288.s001.pdf]

S1 Text. Decoupling between activation time and steady-state level in input-output responses

Giorgio Ravanelli[1,2], Kee-Myoung Nam[3,¤], Jeremy Gunawardena[3,¤b], Rosa Martinez-Corral[1, ¤b,*]

1 CRG (Barcelona Collaboratorium for Modelling and Predictive Biology), C/ Dr Aiguader 88, 08003, Barcelona, Spain.
2 Department of Medicine and Life Sciences, Universitat Pompeu Fabra, Barcelona Biomedical Research Park, Dr Aiguader 88, Barcelona, 08003 Spain.
3 Department of Systems Biology, Harvard Medical School, Boston, MA, USA.
¤Current address: Department of Molecular, Cellular and Developmental Biology, Yale University, New Haven, CT, USA.
¤b Current address: Department of Medicine and Life Sciences, Universitat Pompeu Fabra, Barcelona Biomedical Research Park, Dr Aiguader 88, Barcelona, 08003 Spain.

*rosa.martinez-corral@upf.edu

Appendix A. Derivations of steady-state level and activation time (Eqns. 4, 8, and 10), and the Chebotarev–Agaev recurrence (Eqn. 14)

As described in the section "Modeling approach and mathematical setup," we used the graph-theoretic linear framework [1–4] to formulate and analyze the models that we consider in this paper. Here, we provide brief mathematical derivations for our expressions for the steady-state level (Eqn. 4) and activation time (Eqns. 8 and 10). We also provide a description of the Chebotarev–Agaev recurrence (Eqn. 14), building upon the description in the main text. We will use the notation we have established above in what follows.

Derivation of Eqn. 4. First, we provide a short derivation of Eqn. 4, versions of which have also been provided in previous papers [5, 6]. To do this, we define an extended model that explicitly accounts for the copy-number of the molecular readout, M, produced from the system described by the graph, G, with productive vertices $\mathcal{V}_{\mathrm{prod}}(G) \subset \mathcal{V}(G)$. As described above, this model describes the production of M as proceeding at a constant rate, r, from each productive vertex. On the other hand, we assume that degradation occurs at a first-order rate, δn_M, that is independent of the system state. The corresponding Markov process may be viewed as arising from an infinite "copy-number graph" [6], whose vertices, $(i, n_M) \in \mathcal{V}(G) \times \mathbb{R}_{\geq 0}$, keep track of both the system state, $i \in \mathcal{V}(G)$, and the readout copy-number, n_M. The corresponding master equation is given by

$$\frac{dp_{i,n_M}(t)}{dt} = r_i p_{i,n_M-1}(t) + (n_M+1)\,\delta p_{i,n_M+1}(t) - (r_i + \delta n_M)\,p_{i,n_M}(t) + \sum_{j \in \mathcal{V}(G)} \mathcal{L}(G)_{i,j}\,p_{j,n_M}(t),$$

where we have defined

$$r_i = \begin{cases} r & \text{if } i \in \mathcal{V}_{\mathrm{prod}}(G) \\ 0 & \text{otherwise,} \end{cases}$$

and we appropriately omit the first right-hand term when $n_M = 0$. Now, let us assume that $\mathcal{V}(G) = \{1, \ldots, n\}$, and define the vector, $\mathbf{p}_{n_M}(t) = (p_{1,n_M}(t), \ldots, p_{N,n_M}(t))^{\mathrm{T}}$. Then the above master equation implies a corresponding master equation for $\mathbf{p}_{n_M}(t)$, as

$$\frac{d}{dt}\mathbf{p}_{n_M}(t) = \mathbf{R}\mathbf{p}_{n_M-1}(t) + (n_M+1)\,\delta\mathbf{p}_{n_M+1}(t) - (\mathbf{R} + \delta n_M \mathbf{I} - \mathcal{L}(G))\,\mathbf{p}_{n_M}(t), \tag{S1}$$

where $\mathbf{R}$ is the $n \times n$ diagonal matrix with entries $R_{i,i} = r_i$. We now wish to derive an expression for the mean steady-state value of n_M, which is given by

$$\langle n_M \rangle^* = \sum_{n_M=1}^{\infty} n_M \sum_{i \in \mathcal{V}(G)} p^*_{i,n_M} = \mathbf{1}^{\mathrm{T}} \boldsymbol{\mu}^*, \qquad \text{where} \qquad \boldsymbol{\mu}^* = \sum_{n_M=1}^{\infty} n_M \mathbf{p}^*_{n_M},$$

and $\mathbf{1}$ is the all-ones vector of dimension N.

First, let $q_i(t)$ be the marginal probability of the system state $i \in \mathcal{V}(G)$ at time t,

$$q_i(t) = \sum_{n_M=0}^{\infty} p_{i,n_M}(t),$$

and let $\mathbf{q}(t) = (q_1(t), \ldots, q_N(t))^{\mathrm{T}}$. Then Eqn. S1 tells us that

$$\begin{aligned}
\frac{d}{dt}\mathbf{q}(t) &= \sum_{n_M=0}^{\infty} \frac{d}{dt}\mathbf{p}_{n_M}(t) \\
&= \underbrace{\delta \mathbf{p}_1(t) - \mathbf{R}\mathbf{p}_0(t) + \mathcal{L}(G)\,\mathbf{p}_0(t)}_{n_M=0} + \underbrace{\mathbf{R}\mathbf{p}_0(t) + 2\delta \mathbf{p}_2(t) - \mathbf{R}\mathbf{p}_1(t) - \delta \mathbf{p}_1(t) + \mathcal{L}(G)\,\mathbf{p}_1(t)}_{n_M=1} \\
&\quad + \underbrace{\mathbf{R}\mathbf{p}_1(t) + 3\delta \mathbf{p}_3(t) - \mathbf{R}\mathbf{p}_2(t) - 2\delta \mathbf{p}_2(t) + \mathcal{L}(G)\,\mathbf{p}_2(t)}_{n_M=2} + \cdots,
\end{aligned}$$

which can be easily rearranged as

$$\frac{d}{dt}\mathbf{q}(t) = \mathcal{L}(G)\left(\mathbf{p}_0(t) + \mathbf{p}_1(t) + \mathbf{p}_2(t) + \cdots\right) = \mathcal{L}(G)\,\mathbf{q}(t).$$

Therefore, the steady-state marginal probability vector, $\mathbf{q}^*$, must lie in $\ker \mathcal{L}(G)$, and must in fact equal the vector, $\mathbf{p}^*$, of steady-state probabilities in G (Eqns. 4 and 9).

We are now ready to derive a master equation for the vector,

$$\boldsymbol{\mu}(t) = \sum_{n_M=1}^{\infty} n_M \mathbf{p}_{n_M}(t),$$

whose steady state was defined above. Applying Eqn. S1, we can write

$$\begin{aligned}
\frac{d}{dt}\boldsymbol{\mu}(t) &= \sum_{n_M=1}^{\infty} n_M \frac{d}{dt}\mathbf{p}_{n_M}(t) \\
&= \sum_{n_M=1}^{\infty} n_M \left(\mathbf{R}\mathbf{p}_{n_M-1}(t) + (n_M+1)\,\delta \mathbf{p}_{n_M+1}(t) - (\mathbf{R} + \delta n_M \mathbf{I} - \mathcal{L}(G))\,\mathbf{p}_{n_M}(t)\right),
\end{aligned}$$

which can be reorganized as the sum of three terms,

$$\begin{aligned}
\frac{d}{dt}\boldsymbol{\mu}(t) = \sum_{n_M=1}^{\infty} n_M \mathbf{R}\left(\mathbf{p}_{n_M-1}(t) - \mathbf{p}_{n_M}(t)\right) + \sum_{n_M=1}^{\infty} \delta n_M \left((n_M+1)\,\mathbf{p}_{n_M+1}(t) - n_M \mathbf{p}_{n_M}(t)\right) \\
+ \sum_{n_M=1}^{\infty} n_M \mathcal{L}(G)\,\mathbf{p}_{n_M}(t).
\end{aligned} \tag{S2}$$

We now consider each of the three terms. The first simplifies to

$$\begin{aligned}
\sum_{n_M=1}^{\infty} n_M \mathbf{R}\left(\mathbf{p}_{n_M-1}(t) - \mathbf{p}_{n_M}(t)\right) &= \mathbf{R}\left(\mathbf{p}_0(t) - \mathbf{p}_1(t) + 2\mathbf{p}_1(t) - 2\mathbf{p}_2(t) + 3\mathbf{p}_2(t) - + \cdots\right) \\
&= \mathbf{R}\mathbf{q}(t).
\end{aligned}$$

The second term simplifies to

$$\begin{aligned}
&\sum_{n_M=1}^{\infty} \delta n_M \left((n_M+1)\, \mathbf{p}_{n_M+1}(t) - n_M \mathbf{p}_{n_M}(t) \right) \\
&\quad = \delta \left((2\mathbf{p}_2(t) - \mathbf{p}_1(t)) + 2\left(3\mathbf{p}_3(t) - 2\mathbf{p}_2(t)\right) + \cdots \right) - \delta \left(\mathbf{p}_1(t) + 2\mathbf{p}_2(t) + \cdots \right) \\
&\quad = -\delta \boldsymbol{\mu}(t).
\end{aligned}$$

Finally, the third term is simply

$$\sum_{n_M=1}^{\infty} n_M \mathcal{L}(G)\, \mathbf{p}_{n_M}(t) = \mathcal{L}(G)\, \boldsymbol{\mu}(t).$$

As such, we can rewrite Eqn. S2 as simply

$$\frac{d}{dt}\boldsymbol{\mu}(t) = \mathbf{R}\mathbf{q}(t) - \delta\boldsymbol{\mu}(t) + \mathcal{L}(G)\, \boldsymbol{\mu}(t), \tag{S3}$$

which, at steady state, becomes

$$\delta\boldsymbol{\mu}^* - \mathcal{L}(G)\, \boldsymbol{\mu}^* = \mathbf{R}\mathbf{q}^*.$$

Now, we left-multiply both sides by $\mathbf{1}^{\mathrm{T}}$, to get

$$\delta\mathbf{1}^{\mathrm{T}}\boldsymbol{\mu}^* - \mathbf{1}^{\mathrm{T}}\mathcal{L}(G)\, \boldsymbol{\mu}^* = \mathbf{1}^{\mathrm{T}}\mathbf{R}\mathbf{q}^*.$$

It follows from the definition of $\mathcal{L}(G)$ (Eqn. 2) that the columns of $\mathcal{L}(G)$ sum to zero. Therefore, $\mathbf{1}^{\mathrm{T}}\mathcal{L}(G)$ is the zero vector, and we get

$$\langle n_M \rangle^* = \mathbf{1}^{\mathrm{T}}\boldsymbol{\mu}^* = \frac{\mathbf{1}^{\mathrm{T}}\mathbf{R}\mathbf{q}^*}{\delta}.$$

Now, recalling the definition of $\mathbf{R}$ and the fact that $\mathbf{q}^* = \mathbf{p}^*$, we finally obtain

$$\langle n_M \rangle^* = \frac{1}{\delta} \sum_{i \in \mathcal{V}(G)} r_i p_i^* = \frac{r}{\delta} \sum_{i \in \mathcal{V}_{\mathrm{prod}}(G)} p_i^*,$$

i.e., we recover Eqn. 4.

Derivation of Eqn. 8. We now turn to a derivation of Eqn. 8, which has also been provided in previous work [7]. Recalling the notation introduced above, let G be a strongly connected graph that describes the input-output system of interest, with vertices $\mathcal{V}(G) = \{1, \ldots, n\}$, of which $\mathcal{V}_{\mathrm{prod}}(G) \subset \mathcal{V}(G)$ is the subset of productive vertices; and let G^+ be the graph obtained by adding a new vertex, M, together with edges $j \to M$ for each $j \in \mathcal{V}_{\mathrm{prod}}(G)$. We defined the activation time as the mFPT from a chosen initial vertex, $i \in \mathcal{V}(G)$, to M in the corresponding Markov process, $X^+(t)$, which can be written as

$$\mathrm{mFPT}^i = \mathbb{E}\left[\inf\left\{ t > 0 : X^+(t) = M \right\} \mid X^+(0) = i \right].$$

Now, let $w_i(t)$ denote the probability that a trajectory of $X^+(t)$ that begins at i reaches M by time t,

$$w_i(t) = \Pr\left[X^+(t') = M \text{ for some } 0 \le t' \le t \mid X^+(0) = i \right].$$

Since G is strongly connected, any such trajectory of $X^+(t)$ will eventually reach M with probability one, i.e., $\lim_{t \to 0} w_i(t) = 1$. Now, it can be shown [7] that both $w_i(t)$ and its density, which we denote by $u_i(t) = dw_i/dt$, each satisfies an *adjoint master equation*,

$$\frac{d}{dt}\mathbf{w}(t) = \left(\mathcal{L}(G^+)_{[\overline{\{n+1\}}, \overline{\{n+1\}}]} \right)^{\mathrm{T}} \mathbf{w}(t) \tag{S4}$$

$$\frac{d}{dt}\mathbf{u}(t) = \left(\mathcal{L}(G^+)_{[\overline{\{n+1\}}, \overline{\{n+1\}}]} \right)^{\mathrm{T}} \mathbf{u}(t). \tag{S5}$$

Here, we have defined the vectors $\mathbf{w}(t) = (w_1(t), \ldots, w_n(t))^{\mathrm{T}}$ and $\mathbf{u}(t) = (u_1(t), \ldots, u_n(t))^{\mathrm{T}}$, we have identified $M \equiv n+1$, and $\mathcal{L}(G^+)_{[\overline{\{n+1\}},\overline{\{n+1\}}]}$ is the submatrix of $\mathcal{L}(G^+)$ obtained by removing the row and column corresponding to $M \equiv n+1$.

Now, let $\tilde{u}_i(s)$ be the Laplace transform of $u_i(t)$,

$$\tilde{u}_i(s) = \int_0^\infty e^{-st} u_i(t)\, dt.$$

Taking Laplace transforms of both sides of Eqn. S5, we get

$$s\tilde{\mathbf{u}}(s) - \mathbf{u}(0) = \left(\mathcal{L}(G^+)_{[\overline{\{n+1\}},\overline{\{n+1\}}]}\right)^{\mathrm{T}} \tilde{\mathbf{u}}(s), \tag{S6}$$

where $\tilde{\mathbf{u}}(s) = (\tilde{u}_1(s), \ldots, \tilde{u}_n(s))^{\mathrm{T}}$. To determine the entries in $\mathbf{u}(0)$, we can turn to Eqn. S4, which tells us that

$$u_i(0) = \left.\frac{dw_i}{dt}\right|_{t=0} = \sum_{j \in \mathcal{V}(G)} \ell(i \to j)\, w_j(0) - \left(\sum_{j \in \mathcal{V}(G^+)} \ell(i \to j)\right) w_i(0).$$

Since $w_M(0) = 1$ and $w_j(0) = 0$ for all $j \neq M$, this implies that

$$u_i(0) = \begin{cases} \ell(i \to M) & \text{if } i \in \mathcal{V}_{\text{prod}}(G) \\ 0 & \text{otherwise.} \end{cases}$$

Hence, Eqn. S6 can be rearranged as

$$\left(\mathbf{L}(G^+)_{[\overline{\{n+1\}},\overline{\{n+1\}}]} + s\mathbf{I}\right) \tilde{\mathbf{u}}(s) = \begin{bmatrix} \ell(1 \to M) \\ \vdots \\ \ell(n \to M) \end{bmatrix},$$

or

$$\tilde{\mathbf{u}}(s) = \left(\mathbf{L}(G^+)_{[\overline{\{n+1\}},\overline{\{n+1\}}]} + s\mathbf{I}\right)^{-1} \begin{bmatrix} \ell(1 \to M) \\ \vdots \\ \ell(n \to M) \end{bmatrix}, \tag{S7}$$

where we have defined $\mathbf{L}(G^+) = -\mathcal{L}(G^+)^{\mathrm{T}}$ and we have implicitly set $\ell(i \to M) \equiv 0$ if the edge does not exist.

Now, let $\mu_i^{(r)}$ be the r-th moment of $u_i(t)$,

$$\mu_i^{(r)} = \mathbb{E}\left[\inf\left\{t > 0 : X^+(t) = M\right\}^r \mid X^+(0) = i\right] = \int_0^\infty t^r u_i(t)\, dt.$$

We utilize the simple fact that the moments of $u_i(t)$ are related to $\tilde{u}_i(s)$, as

$$\mu_i^{(r)} = \int_0^\infty t^r u_i(t)\, dt = (-1)^r \frac{d^r}{ds^r} \int_0^\infty e^{-st} u_i(t)\, dt\Big|_{s=0} = (-1)^r \left.\frac{d^r \tilde{u}_i}{ds^r}\right|_{s=0}.$$

Therefore, if we define the vector of moments, $\boldsymbol{\mu}^{(r)} = \left(\mu_1^{(r)}, \ldots, \mu_n^{(r)}\right)^{\mathrm{T}}$, we can rewrite Eqn. S7 as

$$\boldsymbol{\mu}^{(r)} = (-1)^r \frac{d^r}{ds^r} \left(\mathbf{L}(G^+)_{[\overline{\{n+1\}},\overline{\{n+1\}}]} + s\mathbf{I}\right)^{-1}\Bigg|_{s=0} \begin{bmatrix} \ell(1 \to M) \\ \vdots \\ \ell(n \to M) \end{bmatrix},$$

which can be written as [8]

$$\boldsymbol{\mu}^{(r)} = r! \left(\mathbf{L}(G^+)_{[\overline{\{n+1\}},\overline{\{n+1\}}]}\right)^{-(r+1)} \begin{bmatrix} \ell(1 \to M) \\ \vdots \\ \ell(n \to M) \end{bmatrix}.$$

Setting $r = 1$, we finally recover Eqn. 8.

Derivation of Eqn. 10. Finally, we provide a brief derivation of Eqn. 10, again leaving the details to a previous paper [7]. To do this, we turn to the *All-Minors Matrix-Tree theorem* (AMMTT), which relates the *minors* (determinants of submatrices) of $\mathcal{L}(G)$ to the spanning forests of G [9]. It is through this fundamental relationship that the spanning forests of G determine both $\mathrm{SS}(x)$ (Eqns. 4 and 9) and $\mathrm{mFPT}^i(x)$ (Eqn. 10); see [3, 7] for further details.

Let G be a strongly connected graph on vertices $\mathcal{V}(G) = \{1, \ldots, n\}$, and let G^+ be the graph obtained by adding a new vertex, $M \equiv n+1$, as described in the main text. Then the AMMTT tells us that, for any $i, j \in \mathcal{V}(G)$, we have [7]

$$\det \mathcal{L}(G^+)_{[\overline{\{i,n+1\}},\overline{\{j,n+1\}}]} = (-1)^{n-1+i+j}\, w(\Phi_{\{j,n+1\}:i\rightsquigarrow j}(G^+)), \tag{S8}$$

where we have used the weight function, $w(\cdot)$, introduced in the main text; and, in analogy with the notation introduced in Eqn. 8, $\mathcal{L}(G^+)_{[\overline{\{i,n+1\}},\overline{\{j,n+1\}}]}$ is the submatrix of $\mathcal{L}(G^+)$ obtained by removing the rows indexed by $\{i, n+1\}$ and the columns indexed by $\{j, n+1\}$. Meanwhile, the AMMTT also tells us that [2]

$$\det \mathcal{L}(G^+)_{[\overline{\{n+1\}},\overline{\{n+1\}}]} = (-1)^{n}\, w(\Phi_{\{n+1\}}(G^+)). \tag{S9}$$

Now, let us recall Cramer's rule, which states that the inverse of any invertible matrix, $\mathbf{A}$, is given by

$$\mathbf{A}^{-1} = \frac{1}{\det \mathbf{A}} \left(\operatorname{adj} \mathbf{A}\right),$$

where $\operatorname{adj} \mathbf{A}$ is the adjugate matrix of $\mathbf{A}$, whose (i,j)-th entry is given by

$$\left(\operatorname{adj} \mathbf{A}\right)_{i,j} = (-1)^{i+j} \det \mathbf{A}_{[\overline{\{j\}},\overline{\{i\}}]}.$$

Combining this with Eqns. S8 and S9, we can now evaluate the (i,j)-th entry of the matrix $\left(\mathbf{L}(G^+)_{[\overline{\{n+1\}},\overline{\{n+1\}}]}\right)^{-1}$, as

$$\begin{aligned}
\left(\mathbf{L}(G^+)_{[\overline{\{n+1\}},\overline{\{n+1\}}]}\right)^{-1}_{i,j} &= -\left(\mathcal{L}(G^+)_{[\overline{\{n+1\}},\overline{\{n+1\}}]}\right)^{-1}_{j,i} \\
&= -\left(\frac{\operatorname{adj}\left(\mathcal{L}(G^+)_{[\overline{\{n+1\}},\overline{\{n+1\}}]}\right)}{\det \mathcal{L}(G^+)_{[\overline{\{n+1\}},\overline{\{n+1\}}]}}\right)_{j,i} \\
&= -\frac{(-1)^{i+j} \det \mathcal{L}(G^+)_{[\overline{\{i,n+1\}},\overline{\{j,n+1\}}]}}{\det \mathcal{L}(G^+)_{[\overline{\{n+1\}},\overline{\{n+1\}}]}} \\
&= -\frac{(-1)^{n-1+2i+2j}\, w(\Phi_{\{j,n+1\}:i\rightsquigarrow j}(G^+))}{(-1)^{n}\, w(\Phi_{\{n+1\}}(G^+))} \\
&= \frac{w(\Phi_{\{j,n+1\}:i\rightsquigarrow j}(G^+))}{w(\Phi_{\{n+1\}}(G^+))}.
\end{aligned}$$

Now, we combine this with Eqn. 8, to finally obtain

$$\begin{aligned}
\mathrm{mFPT}^i(x) &= \sum_{j=1}^{n} \left(\mathbf{L}(G^+)_{[\overline{\{n+1\}},\overline{\{n+1\}}]}\right)^{-2}_{i,j} \ell(j \to n+1) \\
&= \sum_{j=1}^{n} \left(\mathbf{L}(G^+)_{[\overline{\{n+1\}},\overline{\{n+1\}}]}\right)^{-1}_{i,j} \sum_{k=1}^{n} \left(\mathbf{L}(G^+)_{[\overline{\{n+1\}},\overline{\{n+1\}}]}\right)^{-1}_{j,k} \ell(k \to n+1) \\
&= \sum_{j=1}^{n} \frac{w(\Phi_{\{j,n+1\}:i\rightsquigarrow j}(G^+))}{w(\Phi_{\{n+1\}}(G^+))} \sum_{k=1}^{n} \left(\frac{w(\Phi_{\{k,n+1\}:j\rightsquigarrow k}(G^+))}{w(\Phi_{\{n+1\}}(G^+))}\right) \ell(k \to n+1).
\end{aligned}$$

It is easy to show, using an elementary graph-theoretic argument, that the inner sum evaluates to 1 [7]. This brings us to Eqn. 10.

Chebotarev–Agaev recurrence (Eqn. 14). We conclude this section with a short discussion of the Chebotarev–Agaev recurrence [10]. For any graph Γ with m vertices, let $\mathbf{Q}_k(\Gamma)$ be the $m \times m$ matrix whose (i,j)-th entry, $q_{i,j}^{(k)}(\Gamma)$, is the weight of all spanning forests of Γ in which:

1. there are k edges, or equivalently there are $m-k$ roots;
2. j is a root; and
3. there is a path of edges from i to j.

In our notation, we can denote this as follows. If $i \neq j$, then the (i,j)-th entry of $\mathbf{Q}_k(\Gamma)$ is given by,

$$q_{i,j}^{(k)}(\Gamma) = w\left(\bigcup_{A \subset \mathcal{V}(\Gamma): \#A = m-k,\, i \notin A,\, j \in A} \Phi_{A: i \rightsquigarrow j}(\Gamma) \right), \tag{S10}$$

while the diagonal entries are given by

$$q_{j,j}^{(k)}(\Gamma) = w\left(\bigcup_{A \subset \mathcal{V}(\Gamma): \#A = m-k,\, j \in A} \Phi_{A}(\Gamma) \right). \tag{S11}$$

To build some intuition for what these matrices look like, let us first consider $k = 0$. For both the off-diagonal and diagonal entries, the only subset, A, that could possibly contribute to the union is the entire vertex set, $A = \mathcal{V}(\Gamma) = \{1, \ldots, m\}$. But since $i \in A$ for any vertex $i \in \mathcal{V}(\Gamma)$, this means that the union in Eqn. S10 is in fact empty, and we have

$$q_{i,j}^{(0)}(\Gamma) = 0 \qquad \text{for } i \neq j.$$

On the other hand, A does indeed contribute to the union in Eqn. S11, and we have

$$q_{j,j}^{(0)}(\Gamma) = w(\Phi_A(\Gamma)) = w(\Phi_{\mathcal{V}(\Gamma)}(\Gamma)).$$

The only spanning forest in which every vertex is a root is the edgeless forest. The weight of this forest is the product of an empty set (of edge labels), which by convention evaluates to 1. Therefore, we have

$$q_{j,j}^{(0)}(\Gamma) = 1.$$

Putting these pieces together, we obtain

$$\mathbf{Q}_0(\Gamma) = \mathbf{I}.$$

On the other hand, any spanning forest must have at least one root and at most $m-1$ edges. Therefore, for all $k \geq m$, we have

$$\mathbf{Q}_k(\Gamma) = \mathbf{0}.$$

Now, for all the values of k in between, Chebotarev and Agaev [10] showed that one can calculate $\mathbf{Q}_k(\Gamma)$ via the recurrence relation (Eqn. 14),

$$\mathbf{Q}_{k+1}(\Gamma) = -\mathbf{L}(\Gamma)\,\mathbf{Q}_k(\Gamma) + \left(\frac{\operatorname{tr}\left(\mathbf{L}(\Gamma)\,\mathbf{Q}_k(\Gamma)\right)}{k+1} \right) \mathbf{I},$$

where $\operatorname{tr}(\cdot)$ denotes the trace, and the recurrence is initialized with $\mathbf{Q}_0(\Gamma) = \mathbf{I}$, as discussed above.

To see how this relation helps us derive analytical formulas for $\mathrm{SS}(x)$ and $\mathrm{mFPT}^i(x)$, let us first set $\Gamma = G$, in which case $m = n$. If we take $k = n-1$ in Eqn. S11, we see that the only possible choice of A in the union is $A = \{j\}$. Therefore, we have

$$q_{j,j}^{(n-1)}(G) = w(\Phi_{\{j\}}(G)),$$

which, by Eqn. 9, is equal to ρ_j. Therefore, we can obtain $\mathrm{SS}(x)$ by calculating $\mathbf{Q}_{n-1}(G)$ via the recurrence, extracting its diagonal entries, and normalizing by their sum to get the steady-state probability vector, $\mathbf{p}^*$ (Eqn. 9), from which we can calculate $\mathrm{SS}(x)$ per Eqn. 4.

For the mFPT (Eqn. 10), we require the doubly-rooted spanning forests of $\Gamma = G^+$, whose weights contribute to $\mathbf{Q}_{n-1}(G^+)$. (Recall that G^+ contains $m = n+1$ vertices, due to the inclusion of the terminal vertex M.) In particular, we want the weight,

$$w(\Phi_{\{j,M\}:i\rightsquigarrow j}(G^+)),$$

of all spanning forests rooted at $\{j, M\}$ that contain a path from i to j, for all $j \neq M$. Setting $\Gamma = G^+$ and $k = n-1$ in Eqn. S10, we get

$$q_{i,j}^{(n-1)}(G^+) = w\left(\bigcup_{u\in\mathcal{V}(\Gamma)} \Phi_{\{j,u\}:i\rightsquigarrow j}(G^+)\right).$$

Now, suppose that $j \neq M$. Then, since M is a terminal vertex and has no outgoing edges, every spanning forest of G^+ must have M as a root. Therefore, if $j \neq M$, then the only choice of u for which the set in the above union is nonempty is $u = M$. Therefore, we get

$$q_{i,j}^{(n-1)}(G^+) = w(\Phi_{\{j,M\}:i\rightsquigarrow j}(G^+)),$$

which provides the summands in the right-hand numerator of Eqn. 10. As for the denominator, setting $k = n$ and $j = M \equiv n+1$ in Eqn. S11 gives

$$q_{n+1,n+1}^{(n)}(G^+) = w(\Phi_{\{M\}}(G^+)).$$

Putting these pieces together, we finally obtain

$$\text{mFPT}^i(x) = \sum_{j\in\mathcal{V}(G)} \frac{w(\Phi_{\{j,M\}:i\rightsquigarrow j}(G^+))}{w(\Phi_{\{M\}}(G^+))} = \sum_{j\in\mathcal{V}(G)} \frac{q_{i,j}^{(n-1)}(G^+)}{q_{n+1,n+1}^{(n)}(G^+)},$$

which we state in the main text as Eqn. 13.

Appendix B. Monotonicity of steady-state level and activation time with input concentration under single-rate or coherent regulation

In this section, we outline our strategy for calculating the sign of the derivative of the steady-state level, $\text{SS}(x)$, and activation time, $\text{mFPT}^{U_1}(x)$, with respect to the ligand concentration, x, in the ladder models $\mathcal{D}_2$ and $\mathcal{D}_3$. This will allow us to show that, under certain assumptions, the two outputs are monotonic in x. Due to the complexity of the underlying equations, the analytics is mostly based on symbolic computation using Mathematica and SymPy [11].

In particular, we shall show that, for $\mathcal{D}_2$,

$$\begin{aligned}
\gamma_{1,2} > 1 \quad &\text{and} \quad \gamma_{2,1} = 1 \implies \frac{\partial}{\partial x}\text{SS}(x) > 0 \quad \text{and} \quad \frac{\partial}{\partial x}\text{mFPT}^{U_1}(x) < 0\\
\gamma_{1,2} = 1 \quad &\text{and} \quad \gamma_{2,1} > 1 \implies \frac{\partial}{\partial x}\text{SS}(x) < 0 \quad \text{and} \quad \frac{\partial}{\partial x}\text{mFPT}^{U_1}(x) > 0\\
\gamma_{1,2} < 1 \quad &\text{and} \quad \gamma_{2,1} = 1 \implies \frac{\partial}{\partial x}\text{SS}(x) < 0 \quad \text{and} \quad \frac{\partial}{\partial x}\text{mFPT}^{U_1}(x) > 0\\
\gamma_{1,2} = 1 \quad &\text{and} \quad \gamma_{2,1} < 1 \implies \frac{\partial}{\partial x}\text{SS}(x) > 0 \quad \text{and} \quad \frac{\partial}{\partial x}\text{mFPT}^{U_1}(x) < 0,
\end{aligned}$$

for all $x > 0$. To prove this, we must consider the spanning trees and forests of $\mathcal{D}_N$, which can be used to generate analytical expressions for $\text{SS}(x)$ (Eqns. 4 and 9) and $\text{mFPT}^{U_1}(x)$ (Eqn. 10), as described in the main text and in Appendix A. In particular, Eqn. 9 tells us that the vector, $\boldsymbol{\rho}(G)$, whose corresponding unit vector is the vector of steady-state probabilities, $\mathbf{p}^*(G)$, can be obtained by summing the products of edge labels of all spanning trees rooted at j, for each vertex $j \in \mathcal{V}(G)$. If we set $G = \mathcal{D}_N$, any such spanning tree can contain at most N ligand-binding edges, labeled $k_{\text{on}}x$, and therefore we have, for any $j \in \mathcal{V}(G)$,

$$\rho_j(\mathcal{D}_N) = a_0 + a_1 x + \cdots + a_N x^N,$$

where $a_0, \ldots, a_N$ are coefficients that do not depend on x. Subsequently normalizing by the coordinate sum of $\boldsymbol{\rho}(\mathcal{D}_N)$ (Eqn. 9) and applying Eqn. 4, it is easy to see that $\mathrm{SS}(x)$ must be a rational function of the form,

$$\mathrm{SS}(x) = \frac{a_{0,\mathrm{SS}} + a_{1,\mathrm{SS}}\, x + \cdots + a_{N,\mathrm{SS}}\, x^N}{b_{0,\mathrm{SS}} + b_{1,\mathrm{SS}}\, x + \cdots + b_{N,\mathrm{SS}}\, x^N}.$$

A similar argument can be made for $\mathrm{mFPT}^{U_1}(x)$, by appealing to Eqn. 10. Here, Eqn. 10 tells us that $\mathrm{mFPT}^{U_1}(x)$ can be calculated as a rational function in the edge labels of $\mathcal{D}_N^+$, where the numerator is the sum of edge label products of all spanning forests of $\mathcal{D}_N^+$ rooted at $\{j, M\}$, for each $j \in \mathcal{V}(\mathcal{D}_N)$, in which there is a path from U_1 to j; and the denominator is the sum of edge label products of all spanning trees of $\mathcal{D}_N^+$ rooted at M. Again, any such spanning forest and spanning tree must contain at most N ligand-binding edges, and therefore we have,

$$\mathrm{mFPT}^{U_1}(x) = \frac{a_{0,\mathrm{mFPT}} + a_{1,\mathrm{mFPT}}\, x + \cdots + a_{N,\mathrm{mFPT}}\, x^N}{b_{0,\mathrm{mFPT}} + b_{1,\mathrm{mFPT}}\, x + \cdots + b_{N,\mathrm{mFPT}}\, x^N}.$$

Now, let $R(x)$ denote either of the two outputs, $\mathrm{SS}(x)$ or $\mathrm{mFPT}^{U_1}(x)$. Then differentiating R with respect to x yields,

$$\frac{\partial R}{\partial x} = \frac{\left(\sum_{i=1}^N a_{i,R}\, i x^{i-1}\right)\left(\sum_{i=0}^N b_{i,R}\, x^i\right) - \left(\sum_{i=0}^N a_{i,R}\, x^i\right)\left(\sum_{i=1}^N b_{i,R}\, i x^{i-1}\right)}{\left(\sum_{i=0}^N b_{i,R}\, x^i\right)^2}.$$

If we set $N = 2$, then this becomes

$$\frac{\partial R}{\partial x} = \frac{F(x)}{\left(b_{0,R} + b_{1,R}x + b_{2,R}x^2\right)^2},$$

where

$$F(x) = a_{1,R}b_{0,R} - a_{0,R}b_{1,R} + \left(2a_{2,R}b_{0,R} - 2a_{0,R}b_{2,R}\right)x + \left(a_{2,R}b_{1,R} - a_{1,R}b_{2,R}\right)x^2.$$

Since the denominator and x are both positive, the sign of this derivative is determined by the signs of the coefficients of $F(x)$. For $N = 2$, these coefficients are easy to calculate explicitly. Assuming for now that $\gamma_{2,1} = 1$, we can write the coefficients of $R(x) = \mathrm{SS}(x)$ as,

$$\begin{aligned}
a_{1,\mathrm{SS}}b_{0,\mathrm{SS}} - a_{0,\mathrm{SS}}b_{1,\mathrm{SS}} &= k_{\mathrm{off}}k_{\mathrm{on}}\ell_{1,2}\ell_{2,1}^3\left(\gamma_{1,2} - 1\right) + k_{\mathrm{off}}^3k_{\mathrm{on}}\ell_{1,2}\ell_{2,1}\left(\gamma_{1,2} - 1\right) + k_{\mathrm{off}}k_{\mathrm{on}}\ell_{1,2}^3\ell_{2,1}\left(\gamma_{1,2}^2 - \gamma_{1,2}\right)\\
&\quad + k_{\mathrm{off}}k_{\mathrm{on}}\ell_{1,2}^2\ell_{2,1}^2\left(\gamma_{1,2}^2 - 1\right) + k_{\mathrm{off}}^2k_{\mathrm{on}}\ell_{1,2}^2\ell_{2,1}\left(\gamma_{1,2}^2 - 1\right) + 2k_{\mathrm{off}}^2k_{\mathrm{on}}\ell_{1,2}\ell_{2,1}^2\left(\gamma_{1,2} - 1\right)\\
a_{2,\mathrm{SS}}b_{0,\mathrm{SS}} - a_{0,\mathrm{SS}}b_{2,\mathrm{SS}} &= k_{\mathrm{off}}k_{\mathrm{on}}^2\ell_{1,2}\ell_{2,1}^2\left(\gamma_{1,2} - 1\right) + k_{\mathrm{off}}^2k_{\mathrm{on}}^2\ell_{1,2}\ell_{2,1}\left(\gamma_{1,2} - 1\right) + k_{\mathrm{off}}k_{\mathrm{on}}^2\ell_{1,2}^2\ell_{2,1}\left(\gamma_{1,2}^2 - \gamma_{1,2}\right)\\
a_{2,\mathrm{SS}}b_{1,\mathrm{SS}} - a_{1,\mathrm{SS}}b_{2,\mathrm{SS}} &= k_{\mathrm{off}}k_{\mathrm{on}}^3\ell_{1,2}\ell_{2,1}\left(\gamma_{1,2} - 1\right).
\end{aligned}$$

Analogously, for $R(x) = \mathrm{mFPT}^{U_1}(x)$, we have

$$\begin{aligned}
a_{1,\mathrm{mFPT}}b_{0,\mathrm{mFPT}} - a_{0,\mathrm{mFPT}}b_{1,\mathrm{mFPT}} &= \ell_{1,2}k_{\mathrm{off}}k_{\mathrm{on}}r^4\left(1-\gamma_{1,2}\right) + \ell_{1,2}k_{\mathrm{off}}^3k_{\mathrm{on}}r^2\left(1-\gamma_{1,2}\right) + \ell_{1,2}^2k_{\mathrm{on}}r^4\left(\gamma_{1,2}-\gamma_{1,2}^2\right)\\
&+ 2\ell_{1,2}k_{\mathrm{off}}^2k_{\mathrm{on}}r^3\left(1-\gamma_{1,2}\right) + \ell_{1,2}\ell_{2,1}k_{\mathrm{off}}^3k_{\mathrm{on}}r\left(1-\gamma_{1,2}\right) + \ell_{1,2}\ell_{2,1}^3k_{\mathrm{off}}k_{\mathrm{on}}r\left(1-\gamma_{1,2}\right)\\
&+ \ell_{1,2}^3\ell_{2,1}k_{\mathrm{on}}r^2\left(\gamma_{1,2}-\gamma_{1,2}^2\right) + 3\ell_{1,2}^2\ell_{2,1}k_{\mathrm{off}}k_{\mathrm{on}}r^2\left(1-\gamma_{1,2}^2\right)\\
&+ \ell_{1,2}^2\ell_{2,1}k_{\mathrm{off}}^2k_{\mathrm{on}}r\left(1-\gamma_{1,2}^2\right) + \ell_{1,2}^2\ell_{2,1}^2k_{\mathrm{on}}r^2\left(\gamma_{1,2}-\gamma_{1,2}^2\right)\\
&+ \ell_{1,2}^2k_{\mathrm{off}}^2k_{\mathrm{on}}r^2\left(\gamma_{1,2}-\gamma_{1,2}^2\right) + \ell_{1,2}^2\ell_{2,1}^2k_{\mathrm{off}}k_{\mathrm{on}}r\left(1-\gamma_{1,2}^2\right)\\
&+ 2\ell_{1,2}\ell_{2,1}^2k_{\mathrm{off}}^2k_{\mathrm{on}}r\left(1-\gamma_{1,2}\right) + 2\ell_{1,2}^2\ell_{2,1}k_{\mathrm{on}}r^3\left(\gamma_{1,2}-\gamma_{1,2}^2\right)\\
&+ 2\ell_{1,2}^2k_{\mathrm{off}}k_{\mathrm{on}}r^3\left(\gamma_{1,2}-\gamma_{1,2}^2\right) + 3\ell_{1,2}\ell_{2,1}k_{\mathrm{off}}k_{\mathrm{on}}r^3\left(1-\gamma_{1,2}\right)\\
&+ 3\ell_{1,2}\ell_{2,1}^2k_{\mathrm{off}}k_{\mathrm{on}}r^2\left(1-\gamma_{1,2}\right) + 4\ell_{1,2}\ell_{2,1}k_{\mathrm{off}}^2k_{\mathrm{on}}r^2\left(1-\gamma_{1,2}\right)\\
&+ \ell_{1,2}^3\ell_{2,1}k_{\mathrm{off}}k_{\mathrm{on}}r\left(\gamma_{1,2}-\gamma_{1,2}^2\right)\\
a_{2,\mathrm{mFPT}}b_{0,\mathrm{mFPT}} - a_{0,\mathrm{mFPT}}b_{2,\mathrm{mFPT}} &= 2\ell_{1,2}k_{\mathrm{off}}k_{\mathrm{on}}^2r^3\left(1-\gamma_{1,2}\right) + 2\ell_{1,2}k_{\mathrm{off}}^2k_{\mathrm{on}}^2r^2\left(1-\gamma_{1,2}\right) + 2\ell_{1,2}^2k_{\mathrm{on}}^2r^3\left(\gamma_{1,2}-\gamma_{1,2}^2\right)\\
&+ 2\ell_{1,2}\ell_{2,1}k_{\mathrm{off}}^2k_{\mathrm{on}}^2r\left(1-\gamma_{1,2}\right) + 2\ell_{1,2}\ell_{2,1}^2k_{\mathrm{off}}k_{\mathrm{on}}^2r\left(1-\gamma_{1,2}\right)\\
&+ 2\ell_{1,2}^2\ell_{2,1}k_{\mathrm{on}}^2r^2\left(\gamma_{1,2}-\gamma_{1,2}^2\right) + 2\ell_{1,2}^2k_{\mathrm{off}}k_{\mathrm{on}}^2r^2\left(\gamma_{1,2}-\gamma_{1,2}^2\right)\\
&+ 4\ell_{1,2}\ell_{2,1}k_{\mathrm{off}}k_{\mathrm{on}}^2r^2\left(1-\gamma_{1,2}\right) + 2\ell_{1,2}^2\ell_{2,1}k_{\mathrm{off}}k_{\mathrm{on}}^2r\left(\gamma_{1,2}-\gamma_{1,2}^2\right)\\
a_{2,\mathrm{mFPT}}b_{1,\mathrm{mFPT}} - a_{1,\mathrm{mFPT}}b_{2,\mathrm{mFPT}} &= \ell_{1,2}k_{\mathrm{off}}k_{\mathrm{on}}^3r^2\left(1-\gamma_{1,2}\right) + \ell_{1,2}^2k_{\mathrm{on}}^3r^2\left(\gamma_{1,2}-\gamma_{1,2}^2\right) + \ell_{1,2}\ell_{2,1}k_{\mathrm{off}}k_{\mathrm{on}}^3r\left(1-\gamma_{1,2}\right).
\end{aligned}$$

Since all edge labels are positive, these expressions clearly show that signs of $(\partial/\partial x)\,\mathrm{SS}$ and $(\partial/\partial x)\,\mathrm{mFPT}^{U_1}$ are determined by the value of $\gamma_{1,2}$. Namely, if $\gamma_{1,2} > 1$, then all three coefficients of $(\partial/\partial x)\,\mathrm{SS}$ must be positive, whereas the coefficients of $(\partial/\partial x)\,\mathrm{mFPT}^{U_1}$ must be negative. Similarly, if $\gamma_{1,2} < 1$, then all three coefficients of $(\partial/\partial x)\,\mathrm{SS}$ are negative, whereas the coefficients of $(\partial/\partial x)\,\mathrm{mFPT}^{U_1}$ are positive. This proves the desired claim for $N = 2$. An analogous argument can be made for $\gamma_{2,1}$.

For $N = 3$, a similar set of arguments can be used to show that, when the ligand regulates at most two of the ligand-bound transitions ($B_1 \to B_2$, $B_2 \to B_1$, $B_2 \to B_3$, and $B_3 \to B_2$), then the signs of $(\partial/\partial x)\,\mathrm{SS}(x)$ and $(\partial/\partial x)\,\mathrm{mFPT}^{U_1}(x)$ follow the signs given in Table 1. (Here, the "or" in each condition should be interpreted as inclusive, e.g., the first condition requires that either $\gamma_{1,2} > 1$ and $\gamma_{2,3} = 1$, or $\gamma_{1,2} = 1$ and $\gamma_{2,3} > 1$, or $\gamma_{1,2}, \gamma_{2,3} > 1$.) We used Mathematica to calculate the formulas for the coefficients in $\mathrm{SS}(x)$ and $\mathrm{mFPT}^{U_1}(x)$, which are much more complicated than for the $N = 2$ case; given this complexity, we do not provide them here, but they can be found in ċsv files in the GitHub repository: `https://github.com/theobiolab/FPT_paper.git`.

**Condition**	**Sign of** $(\partial/\partial x)\,\mathrm{SS}(x)$	**Sign of** $(\partial/\partial x)\,\mathrm{mFPT}^{U_1}(x)$
$\gamma_{1,2} > 1$ or $\gamma_{2,3} > 1$, $\gamma_{2,1} = \gamma_{3,2} = 1$	+	−
$\gamma_{2,1} > 1$ or $\gamma_{3,2} > 1$, $\gamma_{1,2} = \gamma_{2,3} = 1$	−	+
$\gamma_{1,2} < 1$ or $\gamma_{2,3} < 1$, $\gamma_{2,1} = \gamma_{3,2} = 1$	−	+
$\gamma_{2,1} < 1$ or $\gamma_{3,2} < 1$, $\gamma_{1,2} = \gamma_{2,3} = 1$	+	−
$\gamma_{1,2} > 1$ or $\gamma_{3,2} < 1$, $\gamma_{2,1} = \gamma_{2,3} = 1$	+	−
$\gamma_{1,2} < 1$ or $\gamma_{3,2} > 1$, $\gamma_{2,1} = \gamma_{2,3} = 1$	−	+
$\gamma_{2,1} < 1$ or $\gamma_{2,3} > 1$, $\gamma_{1,2} = \gamma_{3,2} = 1$	+	−
$\gamma_{2,1} > 1$ or $\gamma_{2,3} < 1$, $\gamma_{1,2} = \gamma_{3,2} = 1$	−	+

Table 1. Signs of $(\partial/\partial x)\,\mathrm{SS}(x)$ and $(\partial/\partial x)\,\mathrm{mFPT}^{U_1}(x)$ in the ladder model $\mathcal{D}_3$, for each of the specified regulatory regimes, for all $x > 0$.

Appendix C. Consequences of $\ell_{1,2} = \ell_{2,1}$ and $\ell_{2,3} = \ell_{3,2}$ on $\Delta_{\overline{SS}}$

We show here why $\ell_{3,2} > \ell_{2,3}$ *strengthens* decoupling, by increasing the steady-state dynamic range, $\Delta_{\overline{\mathrm{SS}}}$. A regulatory regime in case 1.III that satisfies $\ell_{1,2} = \ell_{2,1}$ and $\ell_{2,3} = \ell_{3,2}$ has a steady-state dynamic range of,

$$\Delta_{\overline{\mathrm{SS}}} = \frac{2(\gamma_{2,3} - 1)}{3(\gamma_{2,3} + 2)},$$

which, in the limit of large $\gamma_{2,3}$, converges to a maximum value of 2/3. This is because the steady-state level of the readout is nonzero, even when ligand is absent. In particular, if $\ell_{1,2} = \ell_{2,1}$ and $\ell_{2,3} = \ell_{3,2}$, then it is easy to apply Eqn. 9 to find that the steady-state probabilities of the six states at zero ligand concentration are given by,

$$\begin{aligned} p^*_{U_1}(x=0) = p^*_{U_2}(x=0) = p^*_{U_3}(x=0) &= \frac{1}{3} \\ p^*_{B_1}(x=0) = p^*_{B_2}(x=0) = p^*_{B_3}(x=0) &= 0, \end{aligned}$$

whereas, in the limit of large ligand concentration, we instead have,

$$\begin{aligned} \lim_{x\to\infty} p^*_{U_1}(x) = \lim_{x\to\infty} p^*_{U_2}(x) = \lim_{x\to\infty} p^*_{U_3}(x) &= 0 \\ \lim_{x\to\infty} p^*_{B_1}(x) = \lim_{x\to\infty} p^*_{B_2}(x) &= \frac{1}{2+\gamma_{2,3}} \\ \lim_{x\to\infty} p^*_{B_3}(x) &= \frac{\gamma_{2,3}}{2+\gamma_{2,3}}. \end{aligned}$$

Therefore, if $\gamma_{2,3} \gg 1$, then we have,

$$\overline{\mathrm{SS}}(x=0) = p^*_{U_3}(x=0) = \frac{1}{3} \qquad \text{and} \qquad \lim_{x\to\infty} \overline{\mathrm{SS}}(x) = \lim_{x\to\infty} p^*_{B_3}(x) = 1. \tag{S12}$$

Since $\overline{\mathrm{SS}}(x)$ is monotonic in x in this case, this means that $\Delta_{\overline{\mathrm{SS}}} = 2/3$. On the other hand, allowing for $\ell_{3,2} > \ell_{2,3}$ decreases the steady-state probability of U_3 relative to those of U_1 and U_2 at $x = 0$, and therefore decreases the steady-state level of the readout. As a result, this increases $\Delta_{\overline{SS}}$.

Appendix D. Impossibility of decoupling with coherent regulation and equal transition rates in $\mathcal{D}_3$

Here, we show that decoupling is not achievable in the ladder model, $\mathcal{D}_3$, with any coherent regulatory regime in which

$$\gamma_{1,2} \geq 1, \qquad \gamma_{2,3} \geq 1, \qquad \gamma_{2,1} = \gamma_{3,2} = 1, \tag{S13}$$

when we are subject to the constraint,

$$\ell_{1,2} = \ell_{2,1} = \ell_{2,3} = \ell_{3,2}. \tag{S14}$$

In particular, we shall show that, in this regime,

$$\Delta_{\overline{\mathrm{SS}}} < \Delta_{\overline{\mathrm{mFPT}}^{U_1}}. \tag{S15}$$

Using symbolic calculations to evaluate the two dynamic ranges with $\ell_{1,2} = \ell_{2,1}$ and $\ell_{2,3} = \ell_{3,2}$ (Table 2, case 2.I), taking the difference, and additionally setting $\alpha = \ell_{2,3}/\ell_{1,2} = 1$, we get

$$\Delta_{\overline{\mathrm{SS}}} - \Delta_{\overline{\mathrm{mFPT}}^{U_1}} = \frac{2\gamma_{1,2}\gamma_{2,3} - \gamma_{1,2} - 1}{3(\gamma_{1,2}\gamma_{2,3} + \gamma_{1,2} + 1)} - \frac{3\gamma_{1,2}\gamma_{2,3}\beta + 2\gamma_{1,2}\gamma_{2,3} - \gamma_{1,2}\beta - \gamma_{1,2} - \gamma_{2,3}\beta - \beta - 1}{3\gamma_{1,2}\gamma_{2,3}(\beta+1)} = \frac{P}{Q},$$

where

$$\begin{aligned} P = &-\gamma_{1,2}^2\gamma_{2,3}^2\beta - 3\gamma_{1,2}^2\gamma_{2,3}\beta - 2\gamma_{1,2}^2\gamma_{2,3} + \gamma_{1,2}^2\beta + \gamma_{1,2}^2 + \gamma_{1,2}\gamma_{2,3}^2\beta - 2\gamma_{1,2}\gamma_{2,3}\beta - 2\gamma_{1,2}\gamma_{2,3} + 2\gamma_{1,2}\beta \\ &+ 2\gamma_{1,2} + \gamma_{2,3}\beta + \beta + 1 \\ Q = &\, 3\left(\gamma_{1,2}^2\gamma_{2,3}^2\beta + \gamma_{1,2}^2\gamma_{2,3}^2 + \gamma_{1,2}^2\gamma_{2,3}\beta + \gamma_{1,2}^2\gamma_{2,3} + \gamma_{1,2}\gamma_{2,3}\beta + \gamma_{1,2}\gamma_{2,3}\right). \end{aligned}$$

We want to show that this difference is negative whenever $\gamma_{1,2}, \gamma_{2,3} \geq 1$. Since the denominator, Q, is positive, we focus on the numerator, P. If we define $\varepsilon = \gamma_{2,3} - 1 \geq 0$, we can rewrite P as

$$P = -\gamma_{1,2}^2\beta\varepsilon^2 - 5\gamma_{1,2}^2\beta\varepsilon - 3\gamma_{1,2}^2\beta - 2\gamma_{1,2}^2\varepsilon - \gamma_{1,2}^2 + \gamma_{1,2}\beta\varepsilon^2 + \gamma_{1,2}\beta - 2\gamma_{1,2}\varepsilon + \beta\varepsilon + 2\beta + 1, \tag{S16}$$

which we can rearrange further as

$$P = \gamma_{1,2}\beta\varepsilon^2\left(1 - \gamma_{1,2}\right) + \beta\varepsilon\left(1 - 5\gamma_{1,2}^2\right) + \beta\left(\gamma_{1,2} + 2 - 3\gamma_{1,2}^2\right) + \left(1 - \gamma_{1,2}^2\right) - 2\varepsilon\gamma_{1,2}\left(1 + \gamma_{1,2}\right).$$

From here, it is easy to see that, since $\gamma_{1,2} \geq 1$, the first, third, and fourth terms are less than or equal to zero, and the second and fifth terms must be less than zero. In particular, we have

$$\begin{aligned} P &\leq \beta\varepsilon\left(1 - 5\gamma_{1,2}^2\right) - 2\varepsilon\gamma_{1,2}\left(1 + \gamma_{1,2}\right) \\ &\leq -4\beta\varepsilon - 4\varepsilon \\ &< 0, \end{aligned}$$

from which Eqn. S15 follows.

Appendix E. Decoupling via regulation of backward transitions in $\mathcal{D}_3$

Here, we describe the optimisation procedure for regulatory regimes in which the ligand regulates one or both of the backward transitions, $B_2 \to B_1$ and $B_3 \to B_2$, in the ladder model, $\mathcal{D}_3$. The corresponding results are given in S6, S7, and S8 Fig.

We assumed that the ligand regulates one of each pair of reversible transitions, i.e., that it regulates either $B_1 \to B_2$ or $B_2 \to B_1$, and that it regulates either $B_2 \to B_3$ or $B_3 \to B_2$. As such, we considered three regulatory regimes:

1. $\gamma_{2,1}, \gamma_{2,3} \neq 1$ and $\gamma_{1,2} = \gamma_{3,2} = 1$ (S6 Fig);
2. $\gamma_{1,2}, \gamma_{3,2} \neq 1$ and $\gamma_{2,1} = \gamma_{2,3} = 1$ (S7 Fig); and
3. $\gamma_{2,1}, \gamma_{3,2} \neq 1$ and $\gamma_{1,2} = \gamma_{2,3} = 1$ (S8 Fig),

to complement the analyses of the case in which $B_1 \to B_2$ and $B_2 \to B_3$ are regulated ($\gamma_{1,2}, \gamma_{2,3} \neq 1$, $\gamma_{2,1} = \gamma_{3,2} = 1$) on which we have focused for the majority of this paper. To simplify the analysis, we additionally set (S6A Fig, S7A Fig, and S8A Fig)

$$\ell_f = \ell_{1,2} = \ell_{2,3} = r \qquad \text{and} \qquad \ell_b = \ell_{2,1} = \ell_{3,2}.$$

We then used PSO as previously described to solve the following optimisation problem:

$$\begin{aligned} \text{minimize} \quad & f = 1 - \left(\Delta_{\overline{\text{SS}}} - \Delta_{\overline{\text{mFPT}^{U_1}}}\right) \\ \text{subject to} \quad & g_1 = \left|\log_{10}\gamma_{i,j}\right| - k \leq 0 \\ & g_2 = \left|\log_{10}\gamma_{i',j'}\right| - k \leq 0, \end{aligned}$$

where $\gamma_{i,j}$ and $\gamma_{i',j'}$ are the two nontrivial regulatory factors in each regime, and the rates ℓ_f, ℓ_b, k_{off}, and k_{on} were restricted to lie in the range $\left[10^{-4}, 10^4\right]$ in the appropriate units (units of δ for ℓ_f, ℓ_b, and k_{off}, and units of $\delta/\left(1\,\text{c.u.}\right)$ for k_{on}). The optimizations in S6-S7-S8 Figs are performed setting $k = 3$.

Appendix F. Derivation of the activation time $\text{mFPT}^{\langle U \rangle}$ (Eqn. 25)

Here, we provide a derivation of the activation time, $\text{mFPT}^{\langle U \rangle}$, from an equilibrium of initial states (Eqn. 25). Using the notation and terminology established in the main text and in Appendix A, we define this activation time as the mFPT to produce one *additional* molecule of the readout, M, averaged over all possible initial system states

(i.e., the graph vertices) and readout copy-numbers. If we denote by $X(t)$ and $n_M(t)$ the system state and readout copy-number at time t, respectively, we may write this quantity as

$$\mathrm{mFPT}^{\langle U\rangle}(x) = \sum_{n=0}^{\infty} \sum_{i \in \mathcal{V}(G)} p^*_{i,n}(x=0) \cdot \mathbb{E}\left[\inf\{t > 0 : n_M(t) = n+1\} \mid X(0) = i \text{ and } n_M(0) = n\right]. \quad \text{(S17)}$$

Now, in defining the infinite "copy-number graph" that describes this composite Markov process (Appendix A) [6], we impose the assumption that the transition rates between two system states, $i, j \in \mathcal{V}(G)$, does not depend on the readout copy-number. This implies that the mFPT in the above right-hand sum does not depend on $n_M(0) = n$, and is in fact merely given by

$$\mathbb{E}\left[\inf\{t > 0 : n_M(t) = n+1\} \mid X(0) = i \text{ and } n_M(0) = n\right] = \mathrm{mFPT}^i(x).$$

Therefore, we can rewrite Eqn. S17 as

$$\mathrm{mFPT}^{\langle U\rangle} = \sum_{i \in \mathcal{V}(G)} \mathrm{mFPT}^i(x) \sum_{n=0}^{\infty} p^*_{i,n}(x=0).$$

Now, we recall from Appendix A that

$$\sum_{n=0}^{\infty} p^*_{i,n} = q^*_i = p^*_i,$$

so that we may simply write

$$\mathrm{mFPT}^{\langle U\rangle} = \sum_{i \in \mathcal{V}(G)} \left(p^*_i(x=0) \cdot \mathrm{mFPT}^i(x)\right).$$

Now, setting $G = \mathcal{D}_N$, we note that the probability of every ligand-bound vertex is zero in the absence of ligand, i.e., $p^*_{B_i}(x=0) = 0$ for all $i = 1, \ldots, N$. In this case, we get

$$\mathrm{mFPT}^{\langle U\rangle} = \sum_{i=1}^{N} \left(p^*_{U_i}(x=0) \cdot \mathrm{mFPT}^{U_i}(x)\right),$$

i.e., we recover Eqn. 25.

Appendix G. A simple model for *hunchback* regulation by Bicoid and Zelda, related to [12]

In [12], Eck *et al.* examined the effects of the TFs Bicoid (Bcd) and Zelda (Zld) on the transcription of a reporter driven by the well-known *hunchback* minimal enhancer P2 in the *Drosophila* blastoderm. In this system, Bcd exhibits a concentration gradient over the antero-posterior axis of the embryo, whereas Zld concentration is essentially constant [12]. The authors examined the activation time and levels of the reporter. In WT embryos, the activation time was constant throughout the embryo while the transcription levels varied (thus exhibiting decoupling). In Zld null embryos both quantities became coupled. Here, we provide a highly simplified model for this system, in which Zld is modelled implicitly and Bcd is described to simply bind to one regulatory site. The goal is to show how our formalism can be easily adapted to account for specific experimental systems and data, although the construction of a detailed model, with multiple TFs and binding sites and appropriate parameterization, is outside the scope of the present paper.

We use a variation of the ladder model $\mathcal{D}_3$, as shown in S12 Fig, in which Bcd serves as the ligand, and the system transits through three internal regulatory states before producing the molecular readout, which is the reporter mRNA. The first set of transitions, $U_1 \rightleftharpoons U_2$ and $B_1 \rightleftharpoons B_2$, represent chromatin dynamics, whereas the second set of transitions, $U_2 \rightleftharpoons U_3$ and $B_2 \rightleftharpoons B_3$, represent RNA polymerase recruitment and initiation. For simplicity, we assume that each of these sets of transitions is governed by a characteristic timescale, namely the

rates ℓ_1 for the first set and ℓ_2 for the second (S12 Fig), and assume that $\ell_2 \gg \ell_1$. To model Zld's role as a pioneer factor that opens inaccessible chromatin and enables other TFs, including Bcd, to access DNA [12, 13], we assume that it contributes to regulating both $U_1 \to U_2$ and $B_1 \to B_2$, via the regulatory factor $\gamma_{zld} \geq 1$. To model Bcd's role as a TF that activates *hunchback* transcription, we assume that it contributes to regulating both $B_1 \to B_2$ and $B_2 \to B_3$, via the regulatory factor $\gamma_{bcd} > 1$. Finally, we assume that the regulatory effects of Zld and Bcd on $B_1 \to B_2$ are additive, so that its transition rate is given by $(\gamma_{zld} + \gamma_{bcd})\,\ell_1$.

We first consider the case where Zld is absent, so that $\gamma_{zld} = 1$. In this case, the model reduces to an instance of case 2.I (Table 2), but with $\gamma_{1,2} = \gamma_{2,3} = \gamma_{bcd}$. As implied by our results in Fig 3F–J, in this regime, the steady-state level and activation time of the gene are coupled. This can also be observed by directly appealing to the corresponding formula for $\Delta_{\overline{\text{mFPT}}^{U_1}}$ in Table 2. Setting $\gamma_{1,2} = \gamma_{2,3} = \gamma_{bcd}$ and $\alpha = \beta = \ell_2/\ell_1$, we obtain,

$$\Delta_{\overline{\text{mFPT}}^{U_1}} = -\frac{\alpha^2\gamma_{bcd}\left(\gamma_{bcd} - 1\right) + 2\alpha\left(2\gamma_{bcd}^2 - \gamma_{bcd} - 1\right)}{\gamma_{bcd}^2\left(\alpha^2 + 5\alpha\right)}.$$

Since $\gamma_{bcd} > 1$, both terms in the numerator must be positive; therefore, $\Delta_{\overline{\text{mFPT}}^{U_1}} < 0$. Indeed, this is what is found by Eck *et al.* in a *zelda*$^-$ mutant background.

We now turn to the case where Zld is present, so that $\gamma_{zld} \neq 1$. In this case, we obtain the following dynamic ranges for the steady-state and activation time for $\ell_2 \gg \ell_1$:

$$\Delta_{\overline{\text{SS}}} = \frac{\gamma_{zld} + 1 + \dfrac{\gamma_{zld}^2}{\gamma_{bcd}} - \dfrac{\gamma_{zld}^2}{\gamma_{bcd}^2} - \dfrac{\gamma_{zld}}{\gamma_{bcd}^2}}{2\gamma_{zld} + 1 + \dfrac{2\gamma_{zld}^2}{\gamma_{bcd}} + \dfrac{3\gamma_{zld}}{\gamma_{bcd}} + \dfrac{1}{\gamma_{bcd}} + \dfrac{2\gamma_{zld}^2}{\gamma_{bcd}^2} + \dfrac{3\gamma_{zld}}{\gamma_{bcd}^2} + \dfrac{1}{\gamma_{bcd}^2}}$$

$$\Delta_{\overline{\text{mFPT}}^{U_1}} = -\frac{1}{1 + \dfrac{\gamma_{zld}}{\gamma_{bcd}}}.$$

These equations show that, in the limit where the regulatory effect provided by Zld is much greater than that of Bcd ($\gamma_{zld} \gg \gamma_{bcd}$) and the first set of transitions is much slower than the second ($\ell_2 \gg \ell_1$), we obtain decoupling: $\Delta_{\overline{\text{mFPT}}^{U_1}}$ goes to zero while $\Delta_{\overline{\text{SS}}}$ tends to a finite, nonzero value, again in line with the observations of [12].

Appendix H. Notes on the Erlang process, related to [14]

In [14], Alamos *et al.* employed a simple "kinetic barrier" model to describe the onset of transcription after mitosis in early *Drosophila* development. In this model, a synthetic enhancer that harbors one binding site for the morphogen Dorsal (Dl) regulates transcription of a reporter gene, by irreversibly transitioning through a sequence of transcriptionally inactive states, $\text{OFF}_1, \ldots, \text{OFF}_n$, to a terminal, transcriptionally active state, ON, with each transition proceeding with the same rate, $k([\text{Dl}])$, which is given by

$$k([\text{Dl}]) = c\left(\frac{[\text{Dl}]/K_d}{1 + [\text{Dl}]/K_d}\right),$$

where c is a basal rate and K_d is the Dl–DNA dissociation constant. Once in the ON state, the steady-state transcription level is defined in terms of a separate "thermodynamic model" that incorporates Dl and RNA polymerase binding. The mFPT from OFF_1 to ON was used as a measure of the activation time.

Interestingly, Alamos *et al.* used this model to explain decoupling between transcription level and activation time with respect to Dl concentration, but without rate scale separation among the transition rates. This appears to be at odds with our results, which suggest that a system in which a TF coherently promotes production of the readout can exhibit decoupling only when there is rate scale separation (Fig 3). Here, we provide one possible explanation for this apparent discrepancy, as arising from the fact that Alamos *et al.* used a short time window to estimate the mFPT to the ON state. This contrasts with our definition of the mFPT, which is not subject to such truncation.

Alamos *et al.*'s model is an instance of the well-characterised Erlang process, which is a Markov process on a set of $N + 1$ states, $0, \ldots, N$, with a linear sequence of irreversible transitions, $i \to i + 1$ for $i = 0, \ldots, N - 1$, each with

the same transition rate, k. Alamos *et al.* defined the rate k as being time-dependent, because the Dl concentration is dynamic over the observation window. However, for computations of input-output responses, Alamos *et al.* used Dl concentration measurements from a single timepoint, around 7 minutes into nuclear cycle 13 [14, Fig. S8B]. This model can be schematically depicted using the following graph:

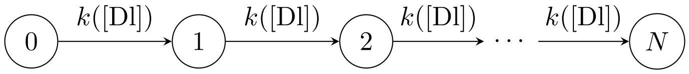

Alamos *et al.* defined the "mean transcription onset time," which corresponds to what we call the activation time, as

$$\mathbb{E}[\text{onset}] = \sum_{i=1}^{m} t_i p_i, \tag{S18}$$

where t_i is one of a set of m possible onset times, $t_1, \ldots, t_m = T$, and p_i is the probability of observing the onset time t_i. (Note that Alamos *et al.* modeled time as a discrete variable, which advances in units of a small timestep dt.) Crucially, Alamos *et al.* assumed that the onset time is capped at some maximal time $t_m = T = 7\,\text{min}$, which represents the time window over which transcriptional activation at the reporter gene was observed in each nucleus. As such, the probabilities p_i are normalized over this window, as

$$\sum_{i=1}^{m} p_i = 1. \tag{S19}$$

Now, the FPT from state 0 to state N in this process is well-known to follow the probability distribution function,

$$f(t) = \frac{k^N t^{N-1} e^{-kt}}{(N-1)!},$$

where we have dropped the dependence of k on [Dl] for clarity. The corresponding cumulative distribution function is given by

$$F(t') = \int_0^{t'} f(t)\,dt = 1 - \sum_{n=0}^{N-1} \frac{(kt')^n\, e^{-kt'}}{n!};$$

this function describes the fraction of nuclei in which transcription has been activated by time t. Note that, since $f(t)$ is a probability distribution on $[0, \infty)$,

$$F(T) = \int_0^T f(t)\,dt < 1.$$

To follow Alamos *et al.*'s assumption and truncate the FPT distribution onto the domain $[0, T]$, we must normalise $f(t)$ by $F(T)$, to get

$$f^{\text{trunc}}(t; T) = \frac{f(t)}{F(T)}.$$

From here, we can define the onset time, as per Alamos *et al.*, as the integral,

$$\mathbb{E}[\text{onset}] = \int_0^T t f^{\text{trunc}}(t; T)\,dt = \frac{1}{F(T)} \int_0^T t \left(\frac{k^N t^{N-1} e^{-kt}}{(N-1)!} \right) dt.$$

Integrating by parts, this can be evaluated as

$$\begin{aligned}\mathbb{E}[\text{onset}] &= \frac{k^N}{F(T)\,(N-1)!}\int_0^T t^N e^{-kt}dt \\ &= \frac{k^N}{F(T)\,(N-1)!}\left(\frac{N!}{k^{N+1}} - \sum_{n=0}^{N}\left(\frac{N!}{k^{n+1}\,(N-n)!}\right)T^{N-n}e^{-kT}\right) \\ &= \frac{k^N}{F(T)\,(N-1)!}\left(\frac{N!}{k^{N+1}}\right)\left(1-\sum_{n=0}^{N}\frac{k^{N-n}T^{N-n}e^{-kT}}{(N-n)!}\right) \\ &= \frac{N}{kF(T)}\left(1-\sum_{n=0}^{N}\frac{(kT)^n\,e^{-kT}}{n!}\right).\end{aligned} \tag{S20}$$

This is distinct from the mean of the untruncated FPT distribution, which is given by

$$\int_0^\infty tf(t)\,dt = \frac{k^N}{(N-1)!}\int_0^\infty t^N e^{-kt}\,dt = \frac{N}{k}. \tag{S21}$$

Now, we sought to compare the untruncated and truncated FPT distributions, $f(t)$ and $f^{\text{trunc}}(t;T)$, and their means, given in Eqns. S21 and S20, respectively (S13A Fig). To follow Alamos *et al.*'s analysis as closely as possible, we assumed that $T = 7\,\text{min}$, and set the coefficients in $k([\text{Dl}])$ to $c = 0.55\,\text{min}^{-1}$ and $K_d = 250\,\text{a.u.}$ [14]. We observed that the untruncated FPT distribution exhibits a strong dependence on the Dl concentration for various choices of N (S13A Fig). In contrast, the truncated FPT distribution shows a much weaker dependence on the Dl concentration, especially for $N = 5$ (S13B–C Fig). This distinction is also visible between the means, as shown in S13D Fig: the untruncated mean decays sharply with Dl concentration for various values of N, whereas the truncated mean—which corresponds to Alamos *et al.*'s definition of onset time—is largely independent of the Dl concentration, especially for $N = 5$. As we describe in the Discussion, these results suggest that Alamos *et al.*'s choice of model only captures the observed decoupling if it enforces a finite time window during which transcription can occur.

Appendix I. Decoupling in an equilibrium re-parametrisation of $\mathcal{D}_3$

In this section, we address the effect of assuming an "equilibrium re-parametrisation" of the $\mathcal{D}_3$ model, in which the input-output system is assumed to reach a steady state of thermodynamic equilibrium. Thermodynamic equilibrium is a special type of steady state in which the system satisfies detailed balance, i.e., no net probability fluxes are observed between any pair of states within the system. A system may only reach thermodynamic equilibrium in the absence of external driving forces, which, in biochemical input-output systems, usually involve ATP hydrolysis [15]. In our graph-based models, a system may reach thermodynamic equilibrium if, and only if, the transition rates are balanced such that each cycle in the graph obeys the *cycle condition*, which states that the product of the edges along a direction in the cycle equals the product along the opposite direction (see [16–18] and recent applications in [1–3, 12, 15, 19]).

Throughout the main text, we assumed that the unbinding rate from each B_i state in the $\mathcal{D}_N$ model is simply $\ell(B_i \to U_i) = k_{\text{off}}$, for all $i = 1, \ldots, N$. One consequence of this is that any such model reaches a steady state *away* from thermodynamic equilibrium, as long as at least one regulatory factor is distinct from 1. For example, in $\mathcal{D}_3$, if the forward transitions $B_1 \to B_2$ and $B_2 \to B_3$ are regulated by the ligand ($\gamma_{1,2}, \gamma_{2,3} \neq 1$), then the product of transition rates along each four-vertex cycle in one direction must be distinct from the corresponding product of transition rates in the opposite direction. For the first cycle, traversing the edges counterclockwise yields the product,

$$\ell(U_1 \to B_1)\,\ell(B_1 \to B_2)\,\ell(B_2 \to U_2)\,\ell(U_2 \to U_1) = (k_{\text{on}}x)\,(\gamma_{1,2}\ell_{1,2})\,k_{\text{off}}\ell_{2,1},$$

whereas traversing the edges clockwise yields the product,

$$\ell(U_1 \to U_2)\,\ell(U_2 \to B_2)\,\ell(B_2 \to B_1)\,\ell(B_1 \to U_1) = \ell_{1,2}\,(k_{\text{on}}x)\,\ell_{2,1}k_{\text{off}}.$$

These two products are distinct, since $\gamma_{1,2} \neq 1$; therefore, the first cycle violates the cycle condition. Similarly, the transition rates in the second cycle also satisfy the inequality,

$$\ell(U_2 \to B_2)\,\ell(B_2 \to B_3)\,\ell(B_3 \to U_3)\,\ell(U_3 \to U_2) \neq \ell(U_2 \to U_3)\,\ell(U_3 \to B_3)\,\ell(B_3 \to B_2)\,\ell(B_2 \to U_2),$$

since $\gamma_{2,3} \neq 1$.

We note that this argument applies to $\mathcal{D}_N$, which lacks the readout production edges, $U_N \to M$ and $B_N \to M$. The actual production of the readout involves coupling to external pools of molecular resources, some of which may not be sufficiently abundant to be assumed to be effectively unchanging while interacting with the system (i.e., the pool is not a "reservoir" [3]), as well as enzyme-catalyzed chemical reactions that often require energy expenditure. As such, it is natural to assume that readout production should occur away from thermodynamic equilibrium. Our point here is that our models *additionally* assume departure from thermodynamic equilibrium within the "regulatory" sub-system represented by $\mathcal{D}_N$, wherein the internal features of the input-output system, together with the bound ligand, expend energy prior to readout production. In the context of gene regulation, examples of such energy-expending mechanisms include chromatin remodeling and histone post-translational modifications [20].

As a first attempt to understand the consequences of assuming this departure from thermodynamic equilibrium, we constructed two "equilibrium re-parametrisations" of $\mathcal{D}_3$, first assuming that only $\gamma_{2,3} \neq 1$, as in Fig 3A–E, then assuming that both $\gamma_{1,2} \neq 1$ and $\gamma_{2,3} \neq 1$, as in Figs 3F–J and 4. In the first re-parametrisation, we redefined the unbinding rate from B_3 as (S14A Fig)

$$\ell(B_3 \to U_3) = \frac{k_{\text{off}}}{\gamma_{2,3}};$$

and in the second re-parametrisation, we redefined the unbinding rates from B_2 and B_3 as (S14C Fig)

$$\ell(B_2 \to U_2) = \frac{k_{\text{off}}}{\gamma_{1,2}} \qquad \text{and} \qquad \ell(B_3 \to U_3) = \frac{k_{\text{off}}}{\gamma_{1,2}\gamma_{2,3}}.$$

It is easy to check that, with either re-parametrization, the model satisfies the cycle condition:

$$\ell(U_1 \to B_1)\,\ell(B_1 \to B_2)\,\ell(B_2 \to U_2)\,\ell(U_2 \to U_1) = \ell(U_1 \to U_2)\,\ell(U_2 \to B_2)\,\ell(B_2 \to B_1)\,\ell(B_1 \to U_1)$$
$$\ell(U_2 \to B_2)\,\ell(B_2 \to B_3)\,\ell(B_3 \to U_3)\,\ell(U_3 \to U_2) = \ell(U_2 \to U_3)\,\ell(U_3 \to B_3)\,\ell(B_3 \to B_2)\,\ell(B_2 \to U_2),$$

and therefore reaches a steady state of thermodynamic equilibrium.

We then took all parameter sets giving rise to the responses in Fig 3D, as well as the parameter sets giving rise to the responses in Fig 4B, and calculated $\overline{\text{SS}}(x)$ and $\overline{\text{mFPT}}^{U_1}(x)$ for their corresponding equilibrium re-parametrizations. For instance, we took from each parameter set giving rise to the responses in Fig 3D the values of k_{off} and $\gamma_{2,3}$, set $\ell(B_3 \to U_3) = k_{\text{off}}/\gamma_{2,3}$, and re-calculated $\overline{\text{SS}}(x)$ and $\overline{\text{mFPT}}^{U_1}(x)$ with this new value for $\ell(B_3 \to U_3)$. Comparing S14B Fig with Fig 3D and S14D Fig with Fig 4B, we found no salient differences in decoupling that arise from introducing these equilibrium re-parametrisations. We leave a more systematic analysis of the consequences of energy expenditure on decoupling to future work.

Appendix J. Definition of activation time according to the master equation

As described in Appendix A, the copy-number n_M of the molecular readout M can be viewed as a random variable that is governed by its own master equation (Eqn. S3). In some other works in the literature, the activation time is defined as the time required for the mean copy-number, $\langle n_M \rangle$, to reach a given threshold value [21, 22]. Here, we describe how this definition differs from the mFPT that we have used throughout this paper.

Let us consider the random telegraph model, $\mathcal{C}_2$, in Fig 1E, with $\ell_{1,2}(x) = a(x)$ and $\ell_{2,1}(x) = b(x)$ for ease of notation. As described in the main text, the mFPT from 1 to M in the augmented graph, $\mathcal{C}_2^+$, which measures the mFPT to the production of one molecule of M, is given by (Eqn. 17)

$$\text{mFPT}^1(x) = \frac{a + b + r}{ar}. \tag{S22}$$

Meanwhile, the master equation for the mean copy-number, $\langle n_M \rangle$, is given by

$$\frac{d}{dt}\langle n_M \rangle = \frac{d}{dt}\sum_{i=1}^{2} \mu_i(t),$$

where $\boldsymbol{\mu}(t) = (\mu_1(t), \mu_2(t))^{\mathrm{T}}$ is the vector defined in Appendix A. Using Eqn. S3, we can write a master equation for this vector, as

$$\begin{aligned}\frac{d}{dt}\begin{bmatrix}\mu_1(t)\\ \mu_2(t)\end{bmatrix} &= \underbrace{\begin{bmatrix}0 & 0\\ 0 & r\end{bmatrix}}_{=\mathbf{R}}\underbrace{\begin{bmatrix}q_1(t)\\ q_2(t)\end{bmatrix}}_{=\mathbf{q}(t)} - \delta\begin{bmatrix}\mu_1(t)\\ \mu_2(t)\end{bmatrix} + \underbrace{\begin{bmatrix}-a & b\\ a & -b\end{bmatrix}}_{=\mathcal{L}(\mathcal{C}_2)}\begin{bmatrix}\mu_1(t)\\ \mu_2(t)\end{bmatrix}\\ &= \begin{bmatrix}0\\ rq_2(t)\end{bmatrix} + \begin{bmatrix}-\delta - a & b\\ a & -\delta - b\end{bmatrix}\begin{bmatrix}\mu_1(t)\\ \mu_2(t)\end{bmatrix},\end{aligned}$$

where $q_1(t)$ and $q_2(t)$ are the time-dependent probabilities of vertices 1 and 2 in $\mathcal{C}_2$, respectively. From here, we get

$$\begin{aligned}\frac{d\langle n_M \rangle}{dt} &= \frac{d\mu_1}{dt} + \frac{d\mu_2}{dt}\\ &= rq_2(t) - \delta\left(\mu_1(t) + \mu_2(t)\right)\\ &= rq_2(t) - \delta\langle n_M \rangle.\end{aligned} \tag{S23}$$

In turn, $q_2(t)$ satisfies the master equation,

$$\frac{d}{dt}\begin{bmatrix}q_1(t)\\ q_2(t)\end{bmatrix} = \underbrace{\begin{bmatrix}-a & b\\ a & -b\end{bmatrix}}_{=\mathcal{L}(\mathcal{C}_2)}\begin{bmatrix}q_1(t)\\ q_2(t)\end{bmatrix}. \tag{S24}$$

Now, assuming initial values of $q_1(0) = 1$ and $q_2(0) = 0$, it is straightforward to solve for the time-dependent solution for Eqn. S24 from the eigenvalues and eigenvectors of $\mathcal{L}(\mathcal{C}_2)$, as

$$\begin{bmatrix}q_1(t)\\ q_2(t)\end{bmatrix} = \left(\frac{1}{a+b}\right)\begin{bmatrix}b\\ a\end{bmatrix} + \left(\frac{1}{a+b}\right)e^{-(a+b)t}\begin{bmatrix}a\\ -a\end{bmatrix},$$

which we can substitute into Eqn. S23 to get

$$\frac{d\langle n_M \rangle}{dt} = \left(\frac{ra}{a+b}\right)\left(1 - e^{-(a+b)t}\right) - \delta\langle n_M \rangle.$$

This can be rearranged as

$$\frac{d\langle n_M \rangle}{dt} + \delta\langle n_M \rangle = \left(\frac{ra}{a+b}\right)\left(1 - e^{-(a+b)t}\right),$$

which is a first-order differential equation that can be solved by way of an integrating factor of $e^{\delta t}$. This yields the general solution,

$$\begin{aligned}\langle n_M \rangle &= e^{-\delta t}\left(\int e^{\delta t}\left(\frac{ra}{a+b}\right)\left(1 - e^{-(a+b)t}\right)dt + C\right)\\ &= e^{-\delta t}\left(\left(\frac{ra}{a+b}\right)\left(\frac{e^{\delta t}}{\delta} - \frac{e^{-(a+b-\delta)t}}{\delta - a - b}\right) + C\right)\\ &= \left(\frac{ra}{a+b}\right)\left(\frac{1}{\delta} - \frac{e^{-(a+b)t}}{\delta - a - b}\right) + Ce^{-\delta t}.\end{aligned}$$

Assuming an initial value of $\langle n_M \rangle(0) = 0$, we obtain the specific solution,

$$\langle n_M \rangle = \left(\frac{ra}{a+b}\right)\left(\frac{1}{\delta} - \frac{e^{-(a+b)t}}{\delta - a - b}\right) - \left(\frac{ra}{a+b}\right)\left(\frac{1}{\delta} - \frac{1}{\delta - a - b}\right)e^{-\delta t}. \tag{S25}$$

We can then define the activation time as the time by which $\langle n_M \rangle$ first reaches a threshold value, say, $\langle n_M \rangle = 1$.

This solution makes clear what was already suggested in Eqn. S23: this definition of activation time depends on the degradation rate, δ (S15A Fig). Meanwhile, the mFPT in Eqn. S22 does not depend on δ (S15B Fig). This is because the two measures treat degradation in fundamentally different ways. The former measure, based on $\langle n_M \rangle$, simply quantifies the time to which the copy-number of M reaches 1 on average. Since M is dynamically produced and degraded over time, this measure naturally depends on δ. The latter measure, based on $\text{mFPT}^1(x)$, quantifies the time to which the *regulatory system* produces one copy of M, regardless of how many copies of M are present in the environment. As such, this measure merely depends on the internal dynamics of the regulatory system and its ligand-binding state, while ignoring the degradation of M as an entirely separate process that takes place elsewhere. As how these internal dynamics can give rise to decoupling constitutes our main topic of interest in this paper, we chose to focus on the mFPT-based measure; however, we believe that both measures represent reasonable interpretations of activation time (Discussion).

References

1. Gunawardena J. A linear framework for time-scale separation in nonlinear biochemical systems. PLoS One. 2012 May;7(5):e36321.

2. Mirzaev I, Gunawardena J. Laplacian dynamics on general graphs. Bull Math Biol. 2013 Nov;75(11):2118-49.

3. Nam KM, Martinez-Corral R, Gunawardena J. The linear framework: using graph theory to reveal the algebra and thermodynamics of biomolecular systems. Interface Focus. 2022 Aug;12(4):20220013.

4. Nam KM, Gunawardena J. The linear framework II: using graph theory to analyse the transient regime of Markov processes. Frontiers in Cell and Developmental Biology. 2023;11.

5. Sánchez A, Kondev J. Transcriptional control of noise in gene expression. Proc Natl Acad Sci U S A. 2008 Apr;105(13):5081-6.

6. Nasser J, Nam KM, Gunawardena J. A mathematical model clarifies the ABC Score formula used in enhancer-gene prediction. eLife. 2025 Apr.

7. Nam KM, Gunawardena J. Algebraic formulas for first-passage times of Markov processes in the linear framework. Bull Math Biol. 2025;87(161).

8. Bernstein DS. Matrix mathematics: Theory, facts, and formulas - second edition. 2nd ed. Princeton, NJ: Princeton University Press; 2009.

9. Chaiken S. A combinatorial proof of the all minors matrix tree theorem. SIAM J Algebr Discrete Methods. 1982 Sep;3(3):319-29.

10. Chebotarev P, Agaev R. Forest matrices around the Laplacian matrix. Linear Algebra Appl. 2002 Nov;356(1):253-74.

11. Meurer A, Smith CP, Paprocki M, Čertík O, Kirpichev SB, Rocklin M, et al. SymPy: symbolic computing in Python. PeerJ Computer Science. 2017 Jan;3:e103. Available from: https://doi.org/10.7717/peerj-cs.103. doi:10.7717/peerj-cs.103.

12. Eck E, Liu J, Kazemzadeh-Atoufi M, Ghoreishi S, Blythe SA, Garcia HG. Quantitative dissection of transcription in development yields evidence for transcription-factor-driven chromatin accessibility. Elife. 2020 Oct;9.

13. Dufourt J, Trullo A, Hunter J, Fernandez C, Lazaro J, Dejean M, et al. Temporal control of gene expression by the pioneer factor Zelda through transient interactions in hubs. Nat Commun. 2018 Dec;9(1):5194.

14. Alamos S, Reimer A, Westrum C, Turner MA, Talledo P, Zhao J, et al. Minimal synthetic enhancers reveal control of the probability of transcriptional engagement and its timing by a morphogen gradient. Cell Syst. 2023 Mar;14(3):220-36.e3.

15. Estrada J, Wong F, DePace A, Gunawardena J. Information Integration and Energy Expenditure in Gene Regulation. Cell. 2016 Jun;166(1):234-44.

16. Hill TL. Studies in irreversible thermodynamics. IV. Diagrammatic representation of steady state fluxes for unimolecular systems. J Theor Biol. 1966 Apr;10(3):442-59.

17. Schnakenberg J. Network theory of microscopic and macroscopic behavior of Master Equation system. Reviews of Modern Physics. 1976;48(4).

18. Onsager L. Reciprocal relations in irreversible processes. I. Phys Rev. 1931 Feb;37(4):405-26.

19. Biddle JW, Martinez-Corral R, Wong F, Gunawardena J. Allosteric conformational ensembles have unlimited capacity for integrating information. Elife. 2021 Jun;10.

20. Wong F, Gunawardena J. Gene Regulation in and out of Equilibrium. Annu Rev Biophys. 2020 May;49:199-226.

21. Ali MZ, Guharajan S, Parisutham V, Brewster RC. Regulatory properties of transcription factors with diverse mechanistic function. PLoS Comput Biol. 2024 Jun;20(6):e1012194.

22. Ham L, Coomer MA, Öcal K, Grima R, Stumpf MPH. A stochastic vs deterministic perspective on the timing of cellular events. Nature Communications. 2024 Jun;15(1):1-10.
